# Compilation and Network Analyses of Cambrian Food Webs

**DOI:** 10.1371/journal.pbio.0060102

**Published:** 2008-04-29

**Authors:** Jennifer A Dunne, Richard J Williams, Neo D Martinez, Rachel A Wood, Douglas H Erwin

**Affiliations:** 1 Santa Fe Institute, Santa Fe, New Mexico, United States of America; 2 Microsoft Research Limited, Cambridge, United Kingdom; 3 Pacific Ecoinformatics and Computational Ecology Lab, Berkeley, California, United States of America; 4 National Center for Ecological Analysis and Synthesis, Santa Barbara, California, United States of America; 5 Grant Institute, School of GeoSciences, University of Edinburgh, Edinburgh, United Kingdom; 6 Edinburgh Collaborative of Subsurface Science and Engineering, University of Edinburgh, Edinburgh, United Kingdom; 7 Department of Paleobiology, National Museum of Natural History, Washington, D.C., United States of America; Princeton University, United States of America

## Abstract

A rich body of empirically grounded theory has developed about food webs—the networks of feeding relationships among species within habitats. However, detailed food-web data and analyses are lacking for ancient ecosystems, largely because of the low resolution of taxa coupled with uncertain and incomplete information about feeding interactions. These impediments appear insurmountable for most fossil assemblages; however, a few assemblages with excellent soft-body preservation across trophic levels are candidates for food-web data compilation and topological analysis. Here we present plausible, detailed food webs for the Chengjiang and Burgess Shale assemblages from the Cambrian Period. Analyses of degree distributions and other structural network properties, including sensitivity analyses of the effects of uncertainty associated with Cambrian diet designations, suggest that these early Paleozoic communities share remarkably similar topology with modern food webs. Observed regularities reflect a systematic dependence of structure on the numbers of taxa and links in a web. Most aspects of Cambrian food-web structure are well-characterized by a simple “niche model,” which was developed for modern food webs and takes into account this scale dependence. However, a few aspects of topology differ between the ancient and recent webs: longer path lengths between species and more species in feeding loops in the earlier Chengjiang web, and higher variability in the number of links per species for both Cambrian webs. Our results are relatively insensitive to the exclusion of low-certainty or random links. The many similarities between Cambrian and recent food webs point toward surprisingly strong and enduring constraints on the organization of complex feeding interactions among metazoan species. The few differences could reflect a transition to more strongly integrated and constrained trophic organization within ecosystems following the rapid diversification of species, body plans, and trophic roles during the Cambrian radiation. More research is needed to explore the generality of food-web structure through deep time and across habitats, especially to investigate potential mechanisms that could give rise to similar structure, as well as any differences.

## Author Summary

Food webs, which depict the networks of feeding interactions among co-occurring species, display many regularities in their structure. For example, the distributions of links to prey and links from predators, the percentages of omnivores and herbivores, and the mean trophic level of species change systematically with the number of taxa and feeding links in a web. Such “scale-dependent” regularities are formalized by network models based on a few simple link distribution rules that successfully predict the network structure of complex food webs from a variety of habitats. To explore how long such regularities may have persisted, we compiled and analyzed detailed food-web data for two ancient fossil assemblages from the early Paleozoic, when rapid diversification of multicellular species, body plans, and trophic roles occurred. Our analyses show that for most aspects of network structure, the Early Cambrian Chengjiang Shale and Middle Cambrian Burgess Shale food webs are very similar to modern webs. This suggests that there are strong and enduring constraints on the organization of feeding interactions in ecosystems. However, a few differences, particularly in the Chengjiang Shale web, suggest that some aspects of network structure were still in flux during early phases of de novo ecosystem construction.

## Abstract 3

Analyses of Chengjiang and Burgess Shale food-web data suggest that most, but not all, aspects of the trophic structure of modern ecosystems were in place over a half-billion years ago.

## Introduction

Perhaps the most fundamental property of life is its ability to use energy and materials to maintain and reproduce itself, in turn providing energy and materials to support more life. This generation and consumption of biomass enabled the evolution of biological diversity and concomitant trophic structure among early metazoan ecosystems as documented in Cambrian fossil assemblages of the early Paleozoic [1–3]. Whereas virtually all phylum-level body plans first appeared and rapidly diversified by the Middle Cambrian [4,5], several researchers have suggested that shifts in dominant taxa with different functional forms across the Phanerozoic reflect fundamental differences in trophic structure between ancient and more recent ecosystems. For example, dominant marine fauna shifted from trilobites and inarticulate brachiopods in the Cambrian, to articulate brachiopods, bryozoans, and stalked echinoderms in the post-Cambrian Paleozoic, to molluscs in the post-Palaeozoic [6–8]. The ratio of motile to nonmotile animal genera across the Phanerozoic suggests that the prevalence of taxa with different trophic roles was relatively stable at different levels over four long intervals interspersed by rapid transition periods, with higher proportions of nonmotile genera in the early Paleozoic compared with the Cenozoic [9]. More specific to this study, it has been suggested that Cambrian marine communities may have lacked secondary and higher-order predators that are present in modern ecosystems [1]. In contrast, other researchers have hypothesized that modern trophic structure including higher-order predators may have emerged in the Early Cambrian, as diversification of phytoplankton created new opportunities for the evolution and diversification of zooplankton and larger invertebrates, driving rapid expansion of ecological interactions, particularly those related to feeding [10]. We explored whether trophic organization as characterized by food-web structure appears to have undergone substantial change from the early to the recent Phanerozoic. We present well-resolved data on trophic interactions within Cambrian assemblages and analyze these data in the context of current food-web data and theory, and with regard to uncertainty in diet designations.

Recent food-web data from diverse aquatic and terrestrial habitats [11–14] have supported the development of simple models that formalize and successfully predict food-web structure [15–18], comparative analyses of empirical food-web structure [19–25], and models that explain variability in basic food-web properties such as connectance [26]. Extending such approaches to ancient ecosystems has been hampered by assumptions that the fossil record is either too incomplete or lacking in evidence of trophic interactions to generate detailed, species-level food-web data of comparable resolution to modern webs [27–29]. However, increasingly comprehensive taxonomic and autecological analyses of select fossil assemblages with excellent soft-body preservation across a wide range of taxa present new opportunities for compiling well-resolved ancient food-web data. Such data, coupled with careful assessments of uncertainty, can provide the basis for quantitative paleo food-web analyses that extend beyond prior guild-based probabilistic approaches [29], particularly for analyses that are not dependent on abundance information or fine-scale temporal or spatial resolution.

We compiled detailed information on taxa and the likely trophic relationships among them for the Lower Cambrian Chengjiang Shale (∼520 million years ago (Ma)) of eastern Yunnan Province, China, and the Middle Cambrian Burgess Shale (505 Ma) of British Columbia, Canada. Each of these “Conservation Lagerstätten” contains extensive soft-bodied preservation of benthic and nektonic marine invertebrates and some early chordates in the Chengjiang. Preservation is exquisite, allowing details of soft-part anatomy to be examined to the level of individual setae on polychaete annelids and gill filaments on arthropods. The morphology of the Cambrian taxa suggests that they occupy multiple trophic levels, and the record contains many types of evidence to infer consumer–resource relationships [10]. Although systematic preservational biases do exist in each assemblage [30], these are not obviously different than methodological biases affecting recent food-web data. Modern datasets, collected by different researchers for various purposes, always exclude some taxa—particularly those that are cryptic, rare, or small—and most webs have uneven resolution of included taxa, with a tendency towards aggregation of lower trophic level taxa [31]. The observation of shared patterns and trends in network structure across modern webs [15–21] suggests that many aspects of food-web structure produce a strong enough signal to rise above some degree of noise, incompleteness, and bias in the data. These types of studies, and the one presented here, seek to elucidate and compare the basic architecture, or “the most universal, high-level, persistent elements of organization” [32] of ecological trophic networks.

The Cambrian data are necessarily cumulative over time and space, because fine temporal and spatial resolution is not achievable. However, both assemblages were deposited in relatively brief stratigraphic intervals and represent species that could potentially interact due to their likely coexistence within particular pelagic and benthic marine habitats. Modern food-web data range from cumulative [11,33] to finely resolved in time (e.g., seasonal webs) [34] and/or space (e.g., patch-scale webs) [35]. The generally implicit assumption underlying cumulative food-web data is that the set of species in question coexist within a habitat, and representatives of those species have the opportunity over some span of ecological time to interact directly. To the degree possible, such webs provide a cumulative documentation of who eats whom among species within a habitat over multiple years, including interactions that are low frequency or represent a small proportion of consumption. Such cumulative webs are used widely for comparative research to look at whether there are regularities in food-web structure [15–26,31]. More narrowly defined webs at finer temporal or spatial scales or that use strict evidence standards (e.g., identifying trophic interactions via gut contents only) have been useful for characterizing how such constraints influence perceived structure within habitats [35,36] but are generally not used to look for cross-system regularities in network structure. The Cambrian web data assembled here appear comparable to modern cumulative web data and are thus appropriate for analysis of cross-system regularities, even given the somewhat broader temporal scales of the Cambrian data.

Two recent studies used a network modeling approach to explore the potential for secondary extinctions in response to perturbations in ancient food webs [28,29]. The second study incorporated an empirical component by generating sets of possible Permian and Triassic food webs from “metanetwork” data based on relatively coarse assignments of trophic interactions among guilds of species [29]. Specific food webs were stochastically drawn from those metanetworks using a range of link distributions observed in modern food webs [21,29]. In contrast, the present study uses more highly resolved diet designations coupled with explicit quantification of uncertainty associated with those designations and makes no assumption that central aspects of food-web structure such as link distributions are similar between ancient and modern webs. Instead, we test the validity of such assumptions for early Phanerozoic communities, with an explicit focus on key methodological issues including whether the Cambrian food-web data presented have comparable levels of resolution to modern food-web data, and whether the observed Cambrian network structure and associated comparisons with recent web structure are sensitive to uncertainty and possible errors in the data.

## Results

### Cambrian Food-Web Data

**Figure 1:**
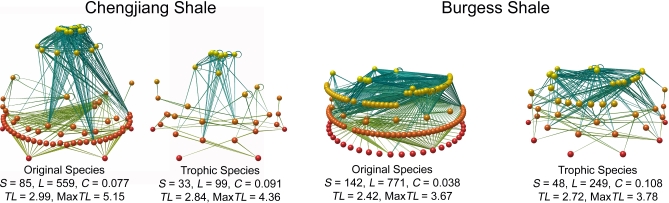
Original- and Trophic-Species Versions of the Chengjiang and Burgess Shale Food Webs Spheres represent taxa, elongated cones represent feeding links. Position of the taxa vertically corresponds to their trophic level (TL), calculated using the short-weighted trophic level algorithm [45], with basal taxa (primary producers and detritus) shown at the bottom of the network in red, and highest trophic level taxa at the top in yellow. S: number of taxa (nodes) in the webs. L: number of trophic links. C: connectance; L/S^2^. MaxTL: maximum trophic level of a species in the web. Images produced with Network3D software written by R. J. Williams; contact ricw@microsoft.com for more detail.

Based on available taxonomic and trophic information (Tables S1–S5), we assembled a food web with 85 taxa for the Chengjiang Shale and one with 142 taxa for the Burgess Shale (Figure 1 and Tables S6 and S7). We refer to these as “original-species webs.” All but five taxa in each original-species web were identified to the species level (Tables S6 and S7). When species with identical consumers and resources within each original-species web are aggregated into trophic species [37], the resulting “trophic-species webs” have 33 taxa (Chengjiang) and 48 taxa (Burgess) (Figure 1, Table 1, and Tables S8–S10). Trophic species are used in comparative food-web studies to reduce methodological and statistical variation due to uneven resolution [37] and insufficient sampling [38] of taxa within and among food webs, and to focus comparative analyses on functionally distinct components of food webs [15]. Following convention [15–26], we focused our analyses on trophic-species webs. The number of taxa in a food web, denoted by S, is a simple measure of diversity sometimes referred to as species richness. Connectance, denoted by C, is a simple measure of food-web complexity calculated as L/S^2^ [11], which quantifies the proportion of possible feeding links L among S taxa that are actually realized. C = 0.091 and 0.108 for the Chengjiang and Burgess Shale trophic-species food webs, respectively (Table 1). Subsequently, we refer to these trophic-species food webs as the Chengjiang web and the Burgess web, or collectively as the Cambrian webs.

**Table 1:**
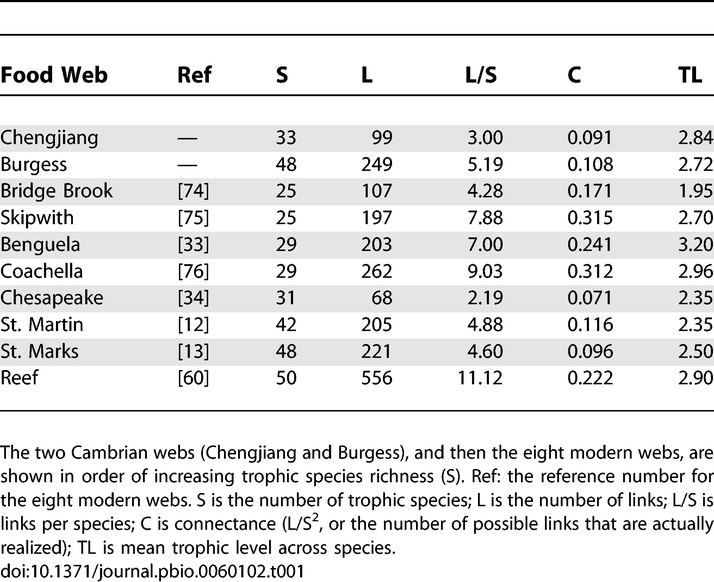
Basic Properties of Two Cambrian and Eight Modern Food Webs

The diversity, complexity, and resolution of the Cambrian webs fall within what is observed for modern webs (Table 1) [31]. The number of taxa in modern trophic-species webs used in recent comparative analyses ranges from ∼25 to 170 [31,39]. The eight modern webs used in this study were selected to represent similar S to the Cambrian webs, with a range of 25–50 taxa. The connectance of the Cambrian webs falls within the range of those webs (0.071–0.315) and is similar to mean C across larger sets of modern webs (∼0.10–0.15) [39]. Mean trophic levels (TLs) for the Chengjiang and Burgess webs (2.84 and 2.72, respectively) fall in the middle of the range for the eight modern webs (1.95–3.20) (Table 1). With over 90% of taxa identified to the species level, the Cambrian original-species webs have higher taxonomic resolution than many modern webs. The Cambrian trophic-species webs have 39% (Chengjiang) and 34% (Burgess) the number of taxa in the original-species webs, which is comparable to aggregation levels for similarly well-resolved modern webs [31]. Poorly resolved original-species webs with coarse taxonomic or trophic categories undergo less trophic-species aggregation, because similar taxa are already grouped.

Each trophic link in the Cambrian webs represents a hypothesis about a feeding relationship based on one or more lines of evidence (Tables S1, S2, S6, and S7). Based on the quality of the evidence (see Materials and Methods section Determination of trophic roles of Cambrian taxa for examples), we assigned a certainty level of 1 (possible), 2 (probable), or 3 (certain) to each link. Of 559 links among 85 taxa in the original-species Chengjiang web, 4.7% are considered certain, 28.3% probable, and 67.1% possible. Of 771 links among 142 taxa in the original-species Burgess web, <1% are considered certain, 53.2% probable, and 46.7% possible. When the original taxa are aggregated into trophic species, certainty is calculated as the average of the certainty of the aggregated links (Table S10). As a result, fewer links are “low-certainty,” which we defined as <1.5 for our analyses. Of 99 and 249 links in the trophic-species versions of the Chengjiang and Burgess webs, 59.6% and 37.3% are low certainty, compared with 67.0% and 46.7% of links in the original-species webs.

**Figure 2:**
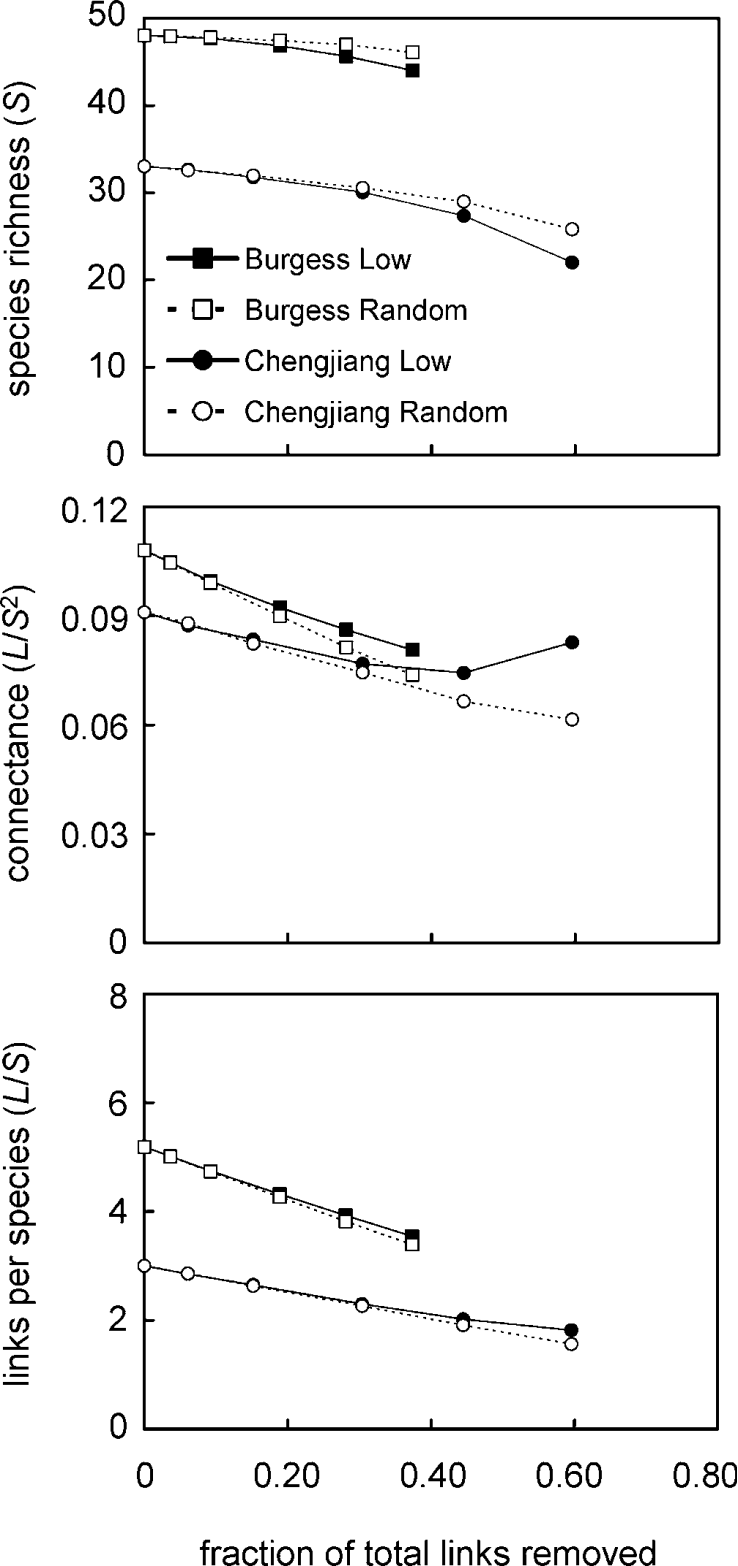
The Response of Species Richness (S), Connectance (L/S^2^) and Links per Species (L/S) to Link Removal in Two Cambrian Food Webs Each data point shows the mean value across 100 webs for that level of link removal (except for 100% removal of low-certainty links and 0% removals, which show single values). Burgess Low and Chengjiang Low show data for removal of low-certainty links, and Burgess Random and Chengjiang Random show data for removal of random links.

**Figure 3:**
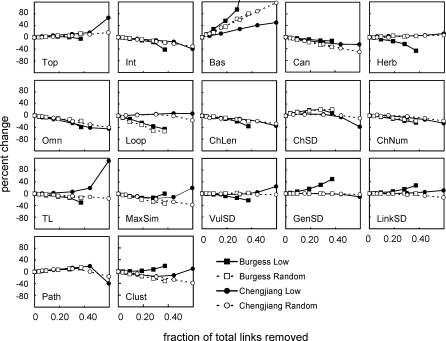
The Response of 17 Structural Properties to Link Removal in Two Cambrian Food Webs The data are shown in terms of percent change, or the difference between each new mean property value and the original property value, divided by the original property value, multiplied by 100. Mean property values are averaged across 100 webs for that level of link removal (except for 100% removal of low-certainty links and 0% removals, which show single values). Burgess Low and Chengjiang Low show data for removal of low-certainty links, and Burgess Random and Chengjiang Random show data for removal of random links.

We tested for systematic bias in the distribution of uncertain links, one source of likely errors in the Cambrian food-web data, by conducting a sensitivity analysis to explore whether the exclusion of low-certainty links and comparable numbers of random links affects Cambrian food-web structure. As increasing proportions of low-certainty or random links were removed, S, C, and L/S declined slowly (Figure 2). Differences in responses to random versus low-certainty link removals were generally small, particularly at less extreme removal levels. In both webs, removal of 30% of total links resulted in the loss of ∼2–3 species, a drop in C of ∼0.02, and a drop in L/S of <1 (Chengjiang) and ∼1 (Burgess). Most of the 17 other topological properties, described in more detail in the Materials and Methods section Network structure properties, also changed relatively little (i.e., percent change < ±20%) with the removal of up to 30% of links, regardless of the web or type of link removal (Figure 3). It was not until extensive removals of more than a third of total links that more than half of the properties exhibited changes greater than ±20%. We feel that it is unlikely that all or even most of the low-certainty links are incorrect; therefore, we suggest that the salient feature is the relative insensitivity of Cambrian network structure to removal of up to ∼30% of total links, equivalent to ∼80% of low-certainty links in the Burgess web and ∼50% of such links in the Chengjiang web.

### Network Structure Comparisons

#### Degree distributions.

Early food-web studies based on poorly resolved data suggested that properties such as the percentage of taxa in a food web that are basal, intermediate, or top are “scale-invariant”—their values are fixed regardless of how many taxa (S) are in the food web [37]. However, subsequent studies based on more highly resolved data indicated that these and other food-web properties are “scale-dependent,” changing systematically with S [40]. More recent studies further suggest that properties can also change systematically with connectance (C), which is based on both S and L [41]. Thus, although the raw values of food-web properties across webs are not invariant, they appear to show other kinds of regularities that depend on the numbers of species and links in the system.

**Figure 4:**
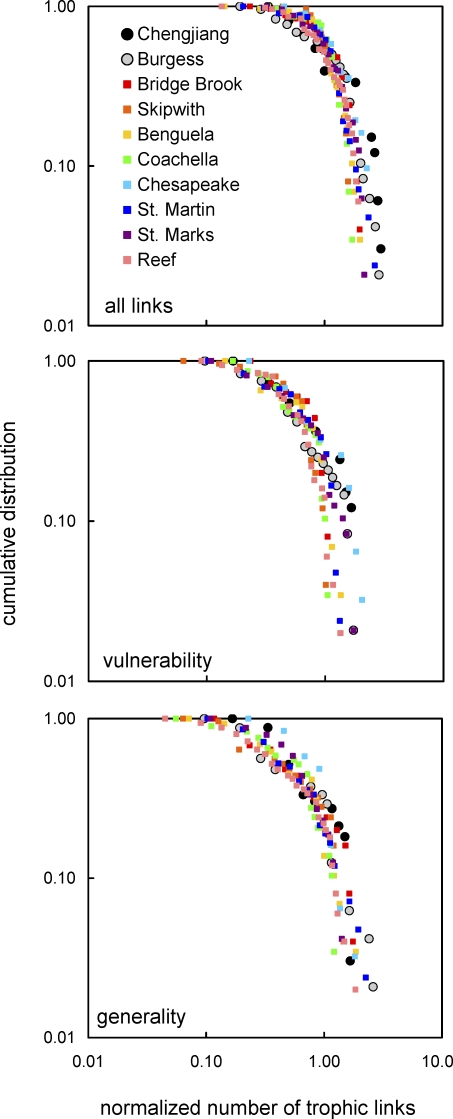
Cumulative Link Distributions for Cambrian and Modern Food Webs The data are presented in log-log format. Cambrian web data are shown with black (Chengjiang) and gray (Burgess) circles; the data for eight modern webs are shown with smaller colored squares. The link data are normalized (divided) by the average number of links per species in each web (i.e., 2L/S).

We used two complementary approaches that normalize for diversity and complexity of individual webs to compare whether Cambrian and modern food webs share scale-dependent regularities in their network structure: (1) a direct comparison of normalized link (“degree”) distributions and (2) an indirect model-based analysis of additional food-web properties. We compared cumulative degree distributions (i.e., the cumulative distributions of the number of feeding links per species) by normalizing each web's link counts by the average number of links per species in that web (2L/S), and overlaying the data from the two Cambrian webs and eight modern webs (Figure 4). Normalized degree distributions of modern food webs, which do not follow a power law [19,21], tend to overlap in a fairly constrained region with some variability [21], which may reveal a universal functional form [17,19]. The similarity of normalized degree distributions of modern food webs suggests that they have at least one central aspect of scale-dependent network structure that is shared across different ecosystems [17,19,21].

The distributions of all links, links from consumers (vulnerability), and links to resources (generality) of the two Cambrian webs generally fall within the variability seen across the eight modern webs, and follow similar non–power-law curves (Figure 4). There are a few Cambrian data points that fall slightly outside the variation seen across the modern webs. In particular, some of the most highly connected species in the Chengjiang web have slightly more links than seen for high-link species in modern webs. Also, some of the least connected species in the Burgess web have slightly fewer links than seen for low-link species in modern webs. Finally, the two species with the most resources in the Burgess web have slightly more resources than seen for high-resource species in modern webs. These slight deviations are not consistently associated with either high or low percentages of uncertain links (Table S11), suggesting that the deviations are not obviously attributable to an overabundance of uncertain links, but are also not highly certain.

#### The niche model.

The use of degree distributions to characterize and compare network structure provides a limited view of topology, because networks that have similar degree distributions can have other properties that differ quite dramatically [18,42]. A number of important properties beyond degree distributions have been calculated for food webs such as percentages of different types of taxa (e.g., basal species, omnivores, cannibals) [15], overall web statistics (e.g., mean trophic level, standard deviation of chain lengths, variability in the numbers of consumers or resources per species) [15], “small-world” properties (mean shortest path length, clustering coefficient) [21], the occurrence of small motifs [20,43], and the scaling of minimum spanning trees [22,24]. A simple network model called the “niche model” [15] successfully reproduces many of these features of empirical food-web structure [15,18,25,31,41,43], in addition to degree distributions [17,19]. The niche model uses S and C as input parameters to set the number of taxa and feeding links to match that of an empirical web. It then uses three rules with stochastic elements to distribute links among nodes, creating a model food web. The use of S and C as inputs takes into account the systematic sensitivity of food-web structure to diversity and complexity. This provides a normalization that allows evaluation of the structural similarity of different empirical webs in terms of how well the niche model predicts their various properties, as quantified by model error (ME). An ME that falls within ±1 indicates good estimation of a property value by the niche model (see Materials and Methods section Niche model analyses). A positive ME indicates overestimation of a property value by the niche model, and a negative ME indicates underestimation.

By generating networks that closely match the structure of modern food webs from a variety of habitats, the niche model has revealed fundamental scale-dependent similarities in their topologies [15,17–19,25,31,41,43]. The relatively good fit of the niche model and recent variants [16,17] to empirical food-web structure compared to the earlier “cascade model” [44] appears associated with their generation of beta link distributions [17], similar to exponential distributions seen in many food webs [19,21]. The niche model, unlike its variants, also assumes trophic intervality (i.e., taxa are arranged along a single-dimension interval and have contiguous feeding ranges represented as segments of the interval), which closely approximates observed trends in empirical data [18,25]. The beta link distribution and intervality constraints at the heart of the niche model, combined with near-hierarchical feeding in contrast to the cascade model's assumption of strict hierarchal feeding, appear to provide much of the niche model's success in predicting empirical food-web degree distributions and other structural properties [17,18,25,43].

We focus here on a subset of 17 single-number properties (see Materials and Methods section Network structure properties, Figure 3) that have been reported in several comparative, model-based studies of food-web structure [15–18,31]. The niche model estimated the central tendency of the 17 structural properties of both Cambrian webs and all eight modern webs well, as indicated by mean MEs over all properties falling well within ±1 for each web, with SDs ranging from 0.626–1.921 (Table 2). Overall, the niche model tended to underestimate property values in the Cambrian webs (mean ME = −0.632 and −0.295 for Chengjiang and Burgess, respectively), while it more closely matched or slightly overestimated property values for modern webs (mean MEs = −0.031 to 0.349). Systematically removing low-certainty or random links had little effect on the overall performance of the niche model. Regardless of type or level of link removal, Cambrian mean MEs fell between 0 and −1, with low-certainty link removals marginally worsening, and random link removals marginally improving, the average niche model fit (Table 3).

**Table 2:**
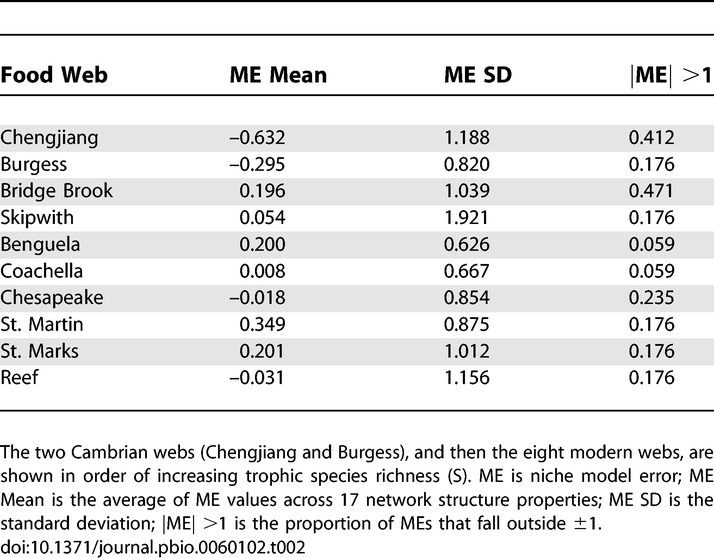
Overall Niche Model Performance for Two Cambrian and Eight Modern Food Webs

**Table 3:**
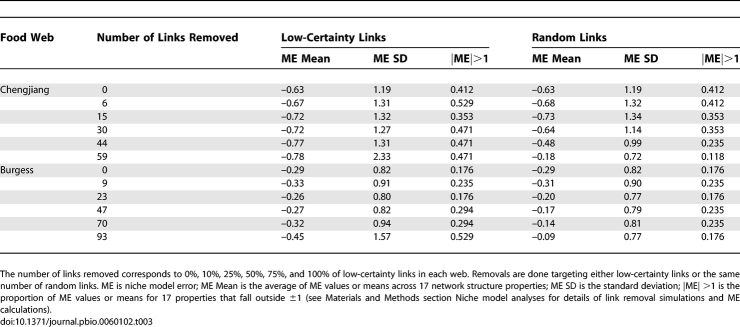
Overall Performance of the Niche Model for Two Cambrian Food Webs with Links Removed

**Figure 5:**
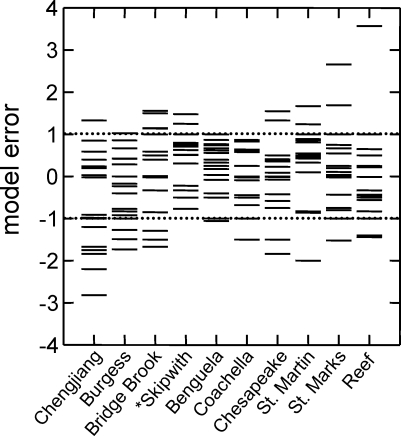
Distribution of Niche Model Errors for Cambrian and Modern Food Webs Each column of stacked horizontal lines shows the MEs for 17 properties for a particular food web. The MEs for the Cambrian webs are shown in the first two columns, and the MEs for eight modern webs, in order of increasing species richness, are shown in the following columns. The dotted horizontal lines show ME = ±1. MEs within this range are considered to show a good fit of the model to the data. One very large ME, Herb = −7.00 for Skipwith, is not shown.

Because of covariances among some properties [17,18] (Materials and Methods section Network structure properties), statistical assessments of model fit are only made at the level of individual properties. The niche model produced good fits for most Cambrian web properties, particularly for the Burgess web. The model errors for 10 of 17 Chengjiang properties fell within ±1 ME (Table 4). The niche model significantly underestimated Path, LinkSD, VulSD, Loop, Can, and Top in order of decreasing absolute ME, and overestimated Bas. For the Burgess web, 14 of 17 MEs fell within ±1. The niche model significantly underestimated Burgess VulSD, LinkSD, and Path. The number of Cambrian web MEs within ±1 falls within the range observed for the modern webs, which spans nine properties for the Bridge Brook web to 16 for the Benguela and Coachella webs (Figure 5, Table 1, and Table S12). The most extreme MEs for the Chengjiang (−2.82, Path) and Burgess (−1.62, VulSD) webs were smaller than those for the Skipwith (−7.00, Herb) and Reef (3.57, MaxSim) webs, with the Burgess extreme ME also smaller than those from the St. Marks, St. Martin, Chesapeake, and Bridge Brook webs (Figure 5, Table 4, and Table S12).

**Table 4:**
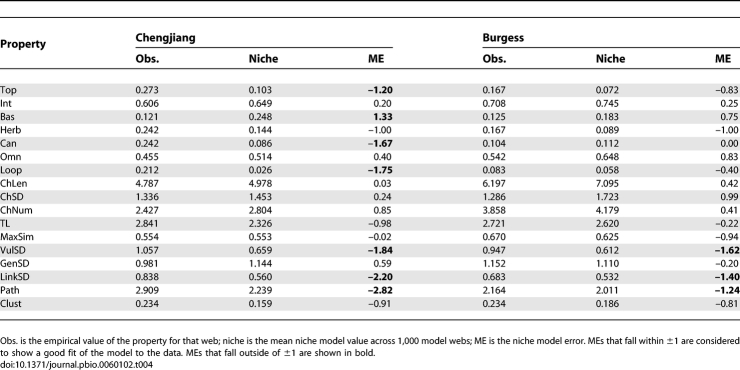
Niche Model Results for 17 Structural Properties of Two Cambrian Food Webs

We conducted two ancillary analyses to demonstrate (1) the fit of the niche model to modern data with known biases and (2) the fit of a simple null model to the Cambrian data (see Materials and Methods section Niche model analyses). In the first analysis, the Ythan and Broom webs had five model errors each that fell outside ±2 including several very large MEs (Ythan: −3.12, −4.70; Broom: −3.81, 4.40, −5.25, −6.86), in addition to 4–5 MEs between 1 and 2 (Table S12). In contrast, the two Cambrian and eight modern webs had few MEs that fell outside of ±2: one property each for Skipwith, St. Marks, Reef, and two properties for Chengjiang (both <|3|) (Table 4 and Table S12). In the second analysis, a null “random-beta model” based on the beta distribution of the niche model, without its near-hierarchical feeding and intervality constraints, did a poor job of estimating Cambrian web structure, with only 2 MEs falling within ±1 for each web, and five (Chengjiang) and nine (Burgess) MEs falling outside of ±2 (Table S13).

**Figure 6:**
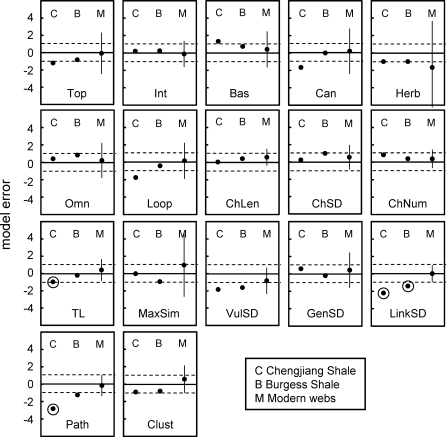
Comparison of Niche Model Errors for Cambrian and Modern Food Webs for 17 Structural Properties For each property, individual ME values are shown for two Cambrian webs, followed by confidence intervals (CI) for eight modern webs, indicated by mean ME ±2.365 standard deviations (SD). Circles around four Cambrian data points indicate Cambrian web MEs that fall outside modern web CIs. 2.365 is the critical value from the *t*-distribution for seven degrees of freedom at a quantile of 97.5% for a two-tailed test. Dashed horizontal lines show ±1 ME boundaries, solid lines show ME = 0. MEs that fall within ±1 are considered to show a good fit of the model to the data.

The most rigorous assessment of how Cambrian web structure compares to modern web structure is presented in Figure 6, which shows MEs for each of the 17 properties for the Cambrian webs and the mean and confidence intervals (CI) of MEs for each property across the eight modern webs. The Cambrian webs' property values fell within the 95% CI for the distributions of property values across the eight modern webs in 30 out of 34 possible cases. The exceptions were Path, LinkSD, and TL of the Chengjiang web and LinkSD of the Burgess web, which fell 1.49, 1.27, 0.16, and 0.47 MEs below the modern webs' lower CIs, respectively. Thus, both Cambrian webs exhibit significantly higher variability than expected in how many links each species has (LinkSD) compared to modern webs. Also, the Chengjiang web has a much longer mean shortest path length (Path) and a marginally higher mean TL than seen in modern webs. The seven other Cambrian property MEs that fell outside of ±1, none of which fell outside of ±2 (Table 4), occurred within the CIs for the modern webs. Link removals had little impact on how Cambrian web structure compares to modern web structure for most properties. Only one property of the Chengjiang web definitively changed its comparative status with link removal. Contrary to the results for the full web, Loop fell well outside modern web CI (Table 5) for all levels of low-certainty link removal and most levels of random removal. In the Burgess web, low-certainty link removals resulted in Path and Clust falling slightly outside of modern web CI at all levels of removal, a pattern that was not seen in the full web or with random link removals (Table 6).

**Table 5:**
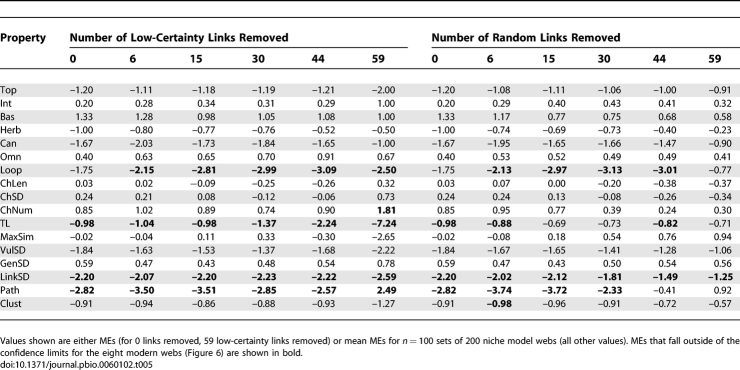
Effect of Link Removal on Niche Model Errors for the Chengjiang Shale Food Web

**Table 6:**
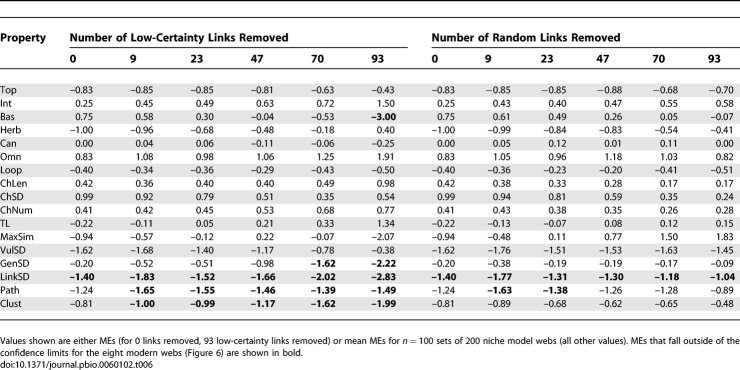
Effect of Link Removal on Niche Model Errors for the Burgess Shale Food Web

To summarize, we observed a few significant differences in the 17 single-number properties between the two Cambrian and eight modern webs. Taking into account both scale-dependence as formalized by the niche model and the uncertainty analysis, both Cambrian webs displayed higher variability in species' total links (LinkSD) and longer mean shortest path lengths (Path), particularly in the Chengjiang web. In addition, the Chengjiang web displayed higher proportions of species in loops (Loop) and higher mean TL than the modern webs, and the Burgess web displayed slightly higher clustering (Clust). Some of these differences may be less meaningful than others. In particular, the expectation that a few of the 34 Cambrian web properties are expected by chance to fall outside modern web CIs suggests that the slightly lower-than-modern MEs (indicating slightly higher-than-modern property values) for Path and Clust for the Burgess web may not reflect real differences. Also, TL and Loop have a dependency due to inclusion of an exact analytical solution for a recursive looping algorithm in the TL calculation, to account for the small fraction of energy passing through a loop passing through it again recursively [45]. Thus, the slightly higher TL in the Chengjiang web may be driven by high levels of Loop. These considerations lead us to suggest that only four of 34 properties display potentially meaningful differences between Cambrian and modern food-web structure: higher LinkSD in both Cambrian webs, and higher Loop and Path in the Chengjiang web. To be clear, these properties appear higher than expected not only in terms of what the niche model predicts for the full and reduced versions of the Cambrian webs, they are higher than expected given the variability we observed across the normalized structures of modern webs, the more critical criterion. If the niche model consistently underestimated those four properties for modern webs, the underestimation of the Cambrian web properties would likely not fall outside the CI for modern webs, and thus the properties would not be judged different between the ancient and modern webs.

#### LinkSD, degree distributions, and the niche model.

**Figure 7:**
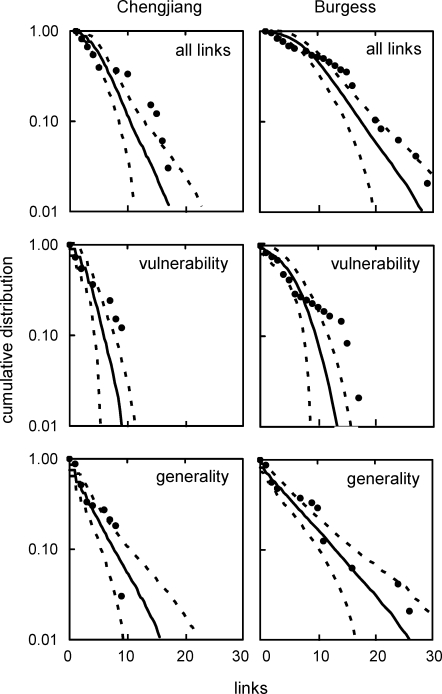
Cumulative Link Distributions for Two Cambrian Food Webs Compared to Niche Model Predictions The data are presented in semilogarithmic format. “All links” graphs show distributions of consumer plus resource links, “vulnerability” graphs show distributions of numbers of consumer species per species (i.e., number of links from consumers), and “generality” graphs show distributions of numbers of resource species per species (i.e., number of links to resources). Filled circles show empirical data. Solid lines show mean niche model simulation results for webs with the same S and C as the two Cambrian webs (*n* = 500), with 95% confidence intervals (±1.96 SD) shown by dashed lines.

The one single-number property that differed significantly and strongly for both Cambrian webs compared to modern webs is LinkSD, the variability in species' total number of links to both resource and consumer taxa. The niche model significantly underestimated LinkSD for the Chengjiang and Burgess webs (ME = −2.20 and −1.40, respectively), but not the modern webs (ME = −0.83 to 0.63). This underestimation appears mostly driven by higher-than-expected VulSD, variability in the number of consumers per species. The niche model more accurately estimated the other LinkSD component, GenSD, which represents variability in the number of resources per species, for the Cambrian webs (Table 4), as illustrated in more detail in the webs' cumulative link distributions compared to niche model predictions (Figure 7). The generality distributions for the Cambrian webs generally fell within or close to the niche model predictions (Figure 7), similar to what has been observed for the eight modern webs (see Figure 6 in [17]). In contrast, the last several points in the vulnerability distributions for both Cambrian webs fell well outside the 95% CIs for niche model simulations, suggesting that the Cambrian taxa with the highest numbers of consumers have more consumers than expected. However, the tendency for taxa in the tails of the vulnerability distributions to have more consumers than expected from the niche model is also seen in many of the eight modern food webs, but with fewer instances of points falling outside niche model CIs (see Figure D1 in [17]). Distributions of all links in the Cambrian webs were more variable and fell further from the mean niche model predictions than link distributions in the eight modern webs (see Figure D2 in [17]), with taxa in the tails of both Cambrian distributions having more links than expected, and taxa near the beginning of the distributions having fewer links than expected (Figure 7), resulting in the high LinksSD. The Cambrian links associated with distribution data points that fell outside the niche model CIs are not associated with consistently high or low levels of uncertainty (Table S11).

## Discussion

The Ediacaran and Early Cambrian have been considered a period of “de novo ecosystem construction” [46], with recently diversified metazoans engaging in novel biotic interactions, thereby constructing new ecological niches [47]. Analyses of Chengjiang and Burgess Shale food-web data suggest that most features of modern ecological network structure were in place by the Early Cambrian. In previous research, comparisons of normalized degree distributions [17,19,21] and other properties using simple network models [15–18,25,31,43] have provided strong evidence supporting three interrelated hypotheses about modern food webs: (1) food-web structure systematically changes with the number of taxa and trophic links in the web, (2) many aspects of food-web structure are well-predicted by the niche model, and (3) food webs from a variety of habitats share many aspects of scale-dependent network structure. Our analyses integrate the two comparative approaches with a novel sensitivity analysis to account for effects of link uncertainty, and our results extend the support for the three hypotheses to two Cambrian food webs. In short, for many aspects of commonly analyzed network structure, the Chengjiang and Burgess Shale food webs share remarkably similar scale-dependent topology with each other and with modern webs, a topology that in most cases is well-characterized by the niche model.

We have presented several lines of evidence supporting this view. The normalized cumulative degree distributions of the Cambrian webs fall within or close to the range of variability seen across the degree distributions for eight modern webs (Figure 4). Niche model analyses indicate that additional aspects of network structure, characterized by various properties of ecological interest, are also similar between the ancient and recent food webs once the effects of differing S and C are accounted for. In both Cambrian and all eight modern webs, the mean niche MEs for 17 properties fall within ±1 (Tables 2 and 3), indicating that the niche model fits the central tendency of the network structure of all ten webs well, consistent with prior results comparing the structure of modern webs from different habitats [15,18,31]. The numbers of MEs that fall outside ±1 for the Cambrian webs are within the range observed for modern webs, and the niche model estimates several modern web properties more poorly than it estimates some Cambrian web properties, particularly with regard to the Burgess web (Table 2 and Figure 5). The niche model also fits the Cambrian web data better than data for two modern webs that are not included in the main analyses and are known to have strong empirical biases (Table 4 and Table S12). The most rigorous comparative model analysis, including results from excluding low-certainty links, shows that only four of 34 property MEs for the two Cambrian webs appear different from modern web property MEs. Thus, multiple ways of looking at the model error results suggest not only that the niche model has similar predictive success for the two Cambrian webs compared with modern webs, but that as a normalization tool it reveals that most Cambrian food-web properties, given scale dependence on S and C, fall within the variability observed across modern web properties. These niche model results reinforce and expand on results from the more direct comparisons of normalized cumulative degree distributions.

The central role of inference in compiling Cambrian food-web data motivated the assignment of certainty levels to trophic links and the subsequent analyses of the impact of excluding 10%–100% of low-certainty links, and equivalent numbers of random links, on observed network structure and comparisons to modern web structure. The results reveal several interesting things about how the exclusion of links affects, or does not affect, our understanding of food-web structure. In most ways, the results were relatively insensitive to link exclusion. Removal of up to ∼30% of total links had only small impacts on the raw values of most structural properties in both Cambrian webs, with some properties showing larger changes at higher link removal levels (Figures 2 and 3). However, even the most extreme link exclusions (i.e., 60% and 37% of total links in the Chengjiang and Burgess webs, respectively) did not affect the overall assessment that the niche model predicts the central tendency of the structure of Cambrian food webs well (Table 3). Together, these findings suggest that even when link exclusions do affect the raw values of some properties, those property values generally change in ways that are consistent with niche model structure and how it scales with C and S, which themselves change slowly with link removal. The relative insensitivity of observed structure to link removals was also noted in a study on network motifs that included analysis of seven food webs and used a model error approach like the one used here. Milo and colleagues reported that “our approach is not sensitive to data errors; for example, the sets of significant network motifs do not change in any of the networks upon addition, removal, or rearrangement of 20% of the edges at random” [20]. We did not systematically add or rearrange links in the Cambrian datasets, because doing so would require making assumptions about link distributions that would drive the outcome in artifactual ways. However, we did analyze an earlier version of the Burgess web with two fewer trophic species that differed from the current version in at least 10% of its links, including 22 fewer links and other rewirings, producing results nearly indistinguishable from the results reported here (unpublished data).

In a few instances, removing links did augment or alter our interpretation of the data. For example, the removal of low-certainty links, especially at higher removal levels, did sometimes affect structure (Figures 2 and 3) and niche model results (Tables 3, 5, and 6) differently than random link removals did, indicating that low-certainty links are not distributed randomly. However, in most cases, the differences were small or did not change comparisons between ancient and modern web properties, and in general did not appear to indicate strong, systematic biases in the distribution of low-certainty links with regard to most aspects of structure examined (see also Table S11). Removal of low-certainty links helped interpretation of which of the few Cambrian property MEs that fell outside modern web confidence intervals most likely indicate meaningful differences, altering initial conclusions about TL and Loop in the Chengjiang web. Sensitivity analyses based on uncertainty of links or taxa would be useful for analysis of the structure of modern cumulative food webs, many of which include inference as a major component of data compilation. Such analyses would complement prior methodological approaches that examined the impact of resolution [11] and sampling effort [38] on food-web structure (see review in [39]).

While degree distribution and model-based analyses suggest that the Cambrian webs generally have similar, scale-dependent network structure to modern webs, in line with prior analyses focused on cross-habitat comparisons [15–19,31,41], there are a few potentially meaningful differences worth brief speculation. In particular, there is significantly higher variability in the distribution of links across species in both Cambrian webs compared to modern webs, as reflected both in LinkSD and related degree distribution analyses (Figures 4, 7 and Table S11). This appears partly driven by taxa with the most consumers having more consumers than expected. The rapid expansion of taxa into novel trophic roles in the early Paleozoic [10,47] may have resulted in a large number of vulnerable taxa that had yet to develop effective predator defenses. A subsequent reduction of very vulnerable taxa could result from their extinction or the development of better defenses in response to the strong selective pressure of having many predators. These ongoing pressures on highly vulnerable taxa could constrain the upper bound of vulnerability to what is observed in modern webs, thus reducing overall variability in both vulnerability and total links.

Two other aspects of structure in the Chengjiang web differed from the later Burgess and modern webs. Mean shortest path length across pairs of taxa in the Chengjiang web is 2.9, much higher than the “two degrees of separation” typically seen in modern webs [41] and the path length of 2.2 observed in the Burgess web. Empirical studies suggest that species separated by more than three links are effectively functionally isolated from each other [41], and a value of Path close to three suggests that many taxa within the Chengjiang web had minimal influence on each other. Mean path length systematically decreases with increased connectance [41], and several structural [48] and dynamical [49–51] studies have suggested that various aspects of food-web stability correlate positively with connectance. This may suggest a trend toward increasing system integration and stability from the Early Cambrian to later food webs. In addition, a higher-than-expected percentage of Chengjiang taxa are found in feeding loops (e.g., where A eats B eats C eats A) than in the later webs, suggesting that trophic organization was less strongly hierarchical in the Chengjiang web. A number of recent studies have shown that hierarchical features of food webs related to consumer-resource body-size ratios are important promoters of stability and persistence [52–55], suggesting that less-hierarchical arrangements are unlikely to persist over long time scales. Thus, the few significant differences between Cambrian, particularly Early Cambrian, and modern food webs may reflect a transition to more strongly constrained, integrated, hierarchical and stable trophic organization within ecosystems following the rapid “Cambrian explosion” diversification of species, body plans, and trophic roles. Analyses of other aspects of network structure not examined here, such as motifs [20,43] or intervality [25], may reveal further similarities or differences between Cambrian and modern food webs.

### Conclusion

The abundance distributions of taxa in marine ecosystems across the Phanerozoic suggest that after the end-Permian extinction, the occurrence of complex species assemblages increased, i.e., assemblages with elevated diversities of mobile and infaunal taxa compared to simpler assemblages dominated by sessile epifaunal suspension feeders [56]. From this point of view, the Chengjiang and Burgess assemblages appear to be early examples of complex metazoan communities. While the occurrence of such complex multi-trophic level communities may have significantly increased in the post-Paleozoic, our results suggest that most aspects of their basic trophic structure were largely in place by the early Paleozoic, despite major changes in the relative dominance of various trophic habits and functional roles [6–9]. The results presented here and elsewhere [15–19,31] suggest that food-web structure, the way that species are organized in terms of their feeding interactions, appears largely independent of the particular identities, morphologies, trophic habits, evolutionary histories, and environmental contexts of species in ecosystems. The question of why food webs across habitats and deep time appear to share a similar architecture that scales systematically with the numbers of species and links in the webs remains open, and may relate to thermodynamic, dynamical stability, and/or evolutionary constraints. The patterns and potential mechanisms of both similarities and differences deserve further research.

The notion that “familiar types of community structure” emerged in the Cambrian was suggested in the mid twentieth century by the eminent ecologist G. Evelyn Hutchinson [57]. Our research indicates that quantitative analysis of the structure of trophic interactions throughout the Phanerozoic is possible and can help in the study of important macroevolutionary questions [28,29], although it requires thoughtful treatment of data scale-dependence, uncertainty, resolution, and incompleteness. For example, carefully selected datasets could indicate whether mass extinctions break patterns of incumbency in trophic complexity and force the construction of new community structures, and whether those new structures converge on the apparently conserved patterns of species interactions suggested by the current and related analyses. However, the strong, scale-dependent similarity of most aspects of network structure between Cambrian and modern webs suggests that such changes over deep time may be subtle and may not exceed variation already documented across modern webs. Convincing demonstration of significant and systematic changes will require careful compilation and analysis of high-quality datasets. Nevertheless, quantitative analysis of ancient food webs promises to open novel areas of research that synthesize studies of structure, function, dynamics, and constraint at the intersection of ecology, evolution, and thermodynamics.

## Materials and Methods

### Determination of trophic roles of Cambrian taxa.

Data relating to species and feeding links are found in Tables S1–S10. We compiled lists of 138 taxa for the Chengjiang Shale [58] and 171 taxa for the Burgess Shale [59] (Tables S1–S3). In addition to species found explicitly in the fossil record, we assumed that five taxa variously used in many modern estuarine and marine food-web datasets [34,60] were present in both Cambrian ecosystems [61,62]: phytoplankton, bacterioplankton, suspended organic matter, benthic detritus, and zooplankton. We refer generically to parasite, predator, herbivore, and detritivore taxa as “consumers,” and anything that they feed on as “resources.” We present information regarding the ecology of Cambrian taxa in Tables S1 and S2 in the columns labeled “Trophic Role,” “Position,” and “Evidence for Trophic Role.” This information was used to assign feeding links among taxa and to assign certainty levels to those links (Tables S6 and S7). Each feeding link was assigned a certainty level of 1 (possible), 2 (probable), or 3 (certain), based on a subjective estimate of the strength of the lines of evidence.

We discuss some general aspects and give a few specific examples of determination of trophic roles and certainty levels here. Direct evidence for food preferences is preserved in a few taxa and is described in original species descriptions as cited (Tables S1 and S2). For example, some fossils of the priapulid worm Ottoia prolifica have preserved gut contents, allowing assignation of a link to Haplophrentis carinatus with the highest level certainty (certainty = 3). However, in the majority of cases, characterization of feeding strategies is inferential. In some cases, the assignment of trophic roles was based on examination of particular morphologic attributes, particularly mouthparts, limb morphology and such predatory features as large eyes. Thus, trilobite feeding roles were assigned based on knowledge of the appendages and the structure of the hypostome, with certainty values of 1 and occasionally 2. We assumed that most predators could feed upon smaller prey available in the same habitat zone. In other cases, particularly for detritus feeding for infaunal forms, the inference is based on eliminating other possibilities, such as predation, based on an absence of apparent predatory features such as grasping spines. For the groups algae, porifera, cnidaria, ctenophora, and brachiopoda, trophic roles are based on knowledge of the characteristics of descendents of the same clade and an assumption of phylogenetic conservation or modern analogs where strongly plausible. Those inferences, assigned certainty levels of 1 or 2, are relevant for only five taxa in the trophic-species versions of each Cambrian web, and account for 11% and 3% of the links in the Chengjiang and Burgess webs, respectively.

As mentioned, algae are assumed to have been photosynthetic based on analogy to modern algae. It has been argued persuasively that herbivory was unlikely in early Palaeozoic ecosystems [63], and despite the abundance of algae, the only possible herbivore we recognized in the Burgess Shale was *Wiwaxia*. Modern sponges, although likely polyphyletic, feed on bacteria and dissolved organic matter. Based on the phylogenetic similarity to extant clades of Chengjiang and Burgess Shale sponges, it seems safe to assume a similar ecology (certainty = 2). Similarly, modern cnidarians and ctenophores are micro-carnivores, and the morphology of the fossil clades provides no basis for arguing that this was not also true of their Cambrian predecessors. Modern brachiopods are largely suspension feeders, and while some may be selective in the categories of food they remove from the water column [64], the modern clades date to the Cambrian, suggesting this ecology is a synapomorphy for the clade (certainty = 2). For the onycophorans *Aysheaia* and *Hallucigenia*, we accepted previous claims that they fed on sponges based on the ecological associations, but since this may not indicate a trophic relationship, we assigned these links a certainty value of 1. Inferring the trophic roles of the annelids is more difficult, but is based on head morphology and the presence or absence of a sediment-filled gut. The last is frankly a tricky criterion. In the past the presence of a sediment-filled gut has been taken as evidence of deposit feeding [59] and its absence as evidence of a more selective food gathering. However the validity of this inference is unclear [62,65].

Arthropods form the bulk of both faunas, both numerically and taxonomically. Fortunately, arthropod limb morphology is often a very useful indicator of trophic role, although it is not always unambiguous. As noted in Tables S1 and S2, we used gut morphology [62]; appendage morphology, particularly the presence of diagnostic structures such as spines or grasping appendages; and mouthpart morphology such as that of *Anomalocaris* or the hypostome of trilobites. *Brachiocaris* evidently lacks eyes, was epibenthic, and has apparent claws on the anterior appendages; from this we infer that it was a scavenger or feeding on sessile animals. The trophic role of the trilobites was based upon the analysis of Fortey and Owen [66] with the assistance of Hughes [67]. For many of the predatory arthropods, we assumed they could feed on smaller arthropods, unless highly specialized appendages were present (certainty = 1). For the larger arthropods, we also assumed that they would be feeding on the younger forms of other large arthropods. Thus *Sancticaris* is shown as feeding on *Anomalocaris* and *Laggania*, not because it was likely able to eat the larger adult forms, but because it could feed on the young. The trophic relationships of *Tuzoia* and *Hurdia* are sparser than for *Anomalocaris*, *Laggania*, and some of the other large arthropods because their morphology, particularly their feeding attributes, is more poorly known, although they are currently under study.

Priapulid worms are found in both assemblages. The predatory feeding role of some can be inferred from the preservation of grasping spines in the introvert (*Ottoia*), while a sediment-filled gut suggests deposit feeding (e.g., *Acosmia*), although as noted above this is a problematic assignment. The predatory role of the chaetognaths has recently been discussed [68], but the function of some of the vetulicolia and the problematica is less certain. Here we have relied on morphology and habitat and as noted sometimes on the elimination of other possibilities. Thus the morphology and semi-sessile habitat of *Vetulocystis* suggests a filter-feeding mode of life [69].

Finally, as indicated in Tables S1 and S2 by cells left blank, there are several taxa for which the data are too imprecise to allow determination of trophic role and/or position.

### Food-web data.

To create Cambrian food-web datasets, we excluded taxa with critically incomplete trophic information, as well as links to those taxa (Tables S4 and S5). Similar to modern food webs, the remaining species and trophic link information for each biota comprises an ancient food web characterized by a single connected network where every animal taxon has at least one food chain leading to a basal taxon and each basal taxon has at least one consumer (Tables S6 and S7). Trophic-species versions of the two original-species Cambrian webs were generated by aggregating taxa within each web that share the same set of consumers and resources (Tables S8–S10 and Figure 1). Certainty levels for links between trophic species were calculated by averaging across certainty levels for all associated aggregated links, resulting in fractional certainty levels in some cases (Table S10).

Trophic-species versions of the two Cambrian food webs (Figure 1 and Tables S8–S10) were compared to eight modern trophic-species food webs from a variety of habitats (two each marine, estuary, lake/pond, and terrestrial; Table 1). We limited analysis to webs with 20 < S < 60 because the two Cambrian webs have S within this range, and there are known scale-dependence issues with analysis of very small webs (i.e., S < 20) [11] and in the use of models to analyze larger webs, which tend to have larger model errors [18]. This represents a strong test for assessing the similarity of Cambrian to modern food-web structure, as the inclusion of smaller or larger webs would increase variability seen in modern web structure, making it more likely that Cambrian structure would fall within modern web variability.

### Network structure properties.

For each food web, we generated cumulative degree distributions for all links, the links from consumers (vulnerability), and the links to resources (generality). In addition, 17 network structure properties [15,18,21] were calculated: Top, Int, and Bas, the fraction of species that are top (without consumers), intermediate (with both consumers and resources), or basal (without resources); Can, Herb, Omn, and Loop, the fraction of species that are cannibals, herbivores (feeding only on basal species), omnivores (species that consume two or more species with different trophic levels), or found in loops (food chains that contain the same species twice, apart from cannibalism); ChLen, ChSD, and ChNum, the mean length, standard deviation of length, and log number of food chains; TL, the mean trophic level of all species computed using the short-weighted trophic level algorithm [18,45]; MaxSim, the mean of the maximum trophic similarity of each species [15,18]; VulSD, GenSD, and LinkSD, the normalized standard deviations of vulnerability, generality, and total links, which measure relative variation in the number of consumers, resources, and consumers plus resources across species [15,18]; and Path, the mean shortest food-chain length between all pairs of species, and Clust, the mean clustering coefficient, the probability that two species linked to the same species are also linked [21,70]. We sometimes refer to these 17 metrics as “single-number properties,” as they quantify different aspects of structure with single numbers, unlike degree distributions. While a few of these properties have analytic forms derivable from generality distributions at the limit of S >> 1 and C << 1 [17], most of them depend on details of food-web structure not captured by link distributions [18]. Although some of these properties are clearly not independent, there is still information to be gained in reporting them separately. For example, Top, Int, and Bas sum to 1, so there are only two degrees of freedom among three properties. While this means that knowing one of the properties places a constraint on the sum of the other two, it does not determine how species are divided among the other two categories. A thorough investigation of the correlation structure among commonly reported food-web properties would be a useful topic for future research.

To quantify the effects of excluding low-certainty or random links on our understanding of the network structure of the Chengjiang and Burgess Shale trophic-species food webs, we conducted a link-removal analysis. We removed the numbers of links that correspond to removal of 10%, 25%, 50%, 75%, and 100% of low-certainty links (i.e., certainty < 1.5) in each web. This corresponds to 6, 15, 30, 44, and 59 links in the Chengjiang web, and 9, 23, 47, 70, and 93 links in the Burgess web. Removals were first done targeting low–certainty links, and in a separate analysis, targeting the same number of random links. One hundred random draws of eligible links were conducted for the 10%, 25%, 50%, and 75% low–certainty link removals (there is only one way to remove 100% of low-certainty links) and at all five levels for random link removals. After each link removal, consumers without resources, consumers without a chain to a basal taxon, and disconnected taxa were removed along with their links, so that the resulting web is a single connected component with an ecologically tenable topology.

To look at overall trends in Cambrian network structure in response to link removal, property values were averaged across the 100 webs resulting from a particular level of link removal. We examined the response of S, C, and L/S to link removals and the percent change in the value of the 17 other properties for the two webs and the two different types of link removal for each level of removal with reference to the original property value.

### Niche model analyses.

To compare empirical food-web structure with structure produced by the niche model, described in detail elsewhere [15,18], we generated ten sets of 1,000 niche-model webs with the same S and C as the ten empirical food webs. For the 17 network structure properties for each of the 1,000 niche model webs, we calculated ME to determine whether the value of a property in an empirical food web differs significantly from the model's distribution of values for that property. A property's ME is calculated as the normalized difference between the model's median property value and the empirical value. Depending on whether the empirical property is higher or lower than the model's median property, the difference is respectively normalized by (i.e., divided by) the difference between the model's median property value and the property value at the upper or lower bound of the central 95% of the 1,000 model values of the property. If the property distribution is one-tailed, the difference is normalized by the difference between the median value and the value at the upper or lower 95% boundary of the distribution. An ME whose absolute value is greater than 1 indicates that the empirical property value does not fall within the most likely 95% of model property values, which we consider indicates that the empirical value significantly differs from the model prediction. This procedure [18] makes no assumptions about the shape of the model's distribution of properties, eliminating statistical errors associated with assumptions of normal distributions of model property values [15,20,31,71].

We checked for confounding scale-dependence in model errors by testing for significant relationships between mean niche model ME (Table 2) and S, L/S, or C across the eight modern webs. No significant linear relationships were found, reinforcing our decision to limit analysis to the selected modern webs (linear regression: *n* = 8: ME = 0.0014S + 0.0705, r^2^ = 0.011, *p* = 0.802; ME = −0.019L/S + 0.241, r^2^ = 0.165, *p* = 0.317; ME = −0.5127C + 0.2188, r^2^ = 0.127, *p* = 0.386). We also checked for the adequacy of *n* = 1,000 for niche model analyses by running five additional sets of *n* = 1,000 niche model webs corresponding to the Chengjiang and Burgess webs, and found that mean niche model values showed little sensitivity to different runs of 1,000, as reflected by low coefficients of variation (<3.6% in all cases; <1% in 27 out of 34 cases) (Table S14).

We also ran basic niche model analyses (*n* = 1,000) on two additional modern food webs with known biases likely to be poorly fit by the niche model. One, a parasite-free version of the Ythan estuary web (“Ythan”) with an overemphasis on birds as top predators [72], is known to be poorly described by the niche model in terms of both single-number properties [15] and degree distribution [17]. The other, a source web of the herbivores, parasitoids, predators, and pathogens associated with the shrub Scotch broom (“Broom”) [73], represents a particular subset of a broader food web, and has a broad-scale link distribution that differs from the single-scale link distributions typical of most food webs [21]. In addition, while it has been shown previously that a null model that distributes links randomly does a very poor job of predicting empirical food-web structure [15], we considered an alternate, more plausible null model that we refer to as the “random-beta model.” This model distributes links using a beta distribution, reproducing one of the central constraints of the niche model [15] and its variants [16,17], but it does not include any other constraints (e.g., near-hierarchical feeding, diet contiguity). We generated two sets of 1,000 random-beta model networks with the same S and C as the two Cambrian webs, and calculated niche model means and MEs for each of the 17 properties for the two webs.

To quantify the effects of excluding low-certainty or random links on our understanding of how the network structure of the Cambrian webs compares to modern web structure, we used the sets of webs generated by systematically removing uncertain or random links to conduct a comparative niche model analysis. For each of the reduced webs (i.e., 100 webs for each level and type of link removal in the two Cambrian webs, with the exception of a single web resulting from 100% removal of low-certainty links in each web), a set of 200 niche model webs at the appropriate S and C was generated, and MEs were calculated for each of the 17 properties. MEs for a particular property were then averaged across the 100 webs generated for each level and type of link removal.

In addition to niche model analyses focused on the 17 single-number properties, we calculated the mean cumulative link distributions (all links, vulnerability, generality) and 95% CIs for separate sets of 500 niche model webs for the two Cambrian webs.

## Supporting Information

Table S1 Master Taxa List for the Chengjiang Shale (138 Taxa) (225 KB DOC)

Table S2 Master Taxa List for the Burgess Shale (171 Taxa) (289 KB DOC)

Table S3 Synonyms for Chengjiang Shale Taxa (73 KB DOC)

Table S4 Chengjiang Shale Taxa Not Included in the Chengjiang Food-Web Dataset (84 KB DOC)

Table S5 Burgess Shale Taxa Not Included in the Burgess Food-Web Dataset (59 KB DOC)

Table S6 Chengjiang Shale Food-Web Data (805 KB DOC)

Table S7 Burgess Shale Food-Web Data (1.07 MB DOC)

Table S8 Chengjiang Shale Trophic Species Groupings (124 KB DOC)

Table S9 Burgess Shale Trophic Species Groupings (194 KB DOC)

Table S10 Trophic Species Food-Web Data for the Chengjiang and Burgess Shales (201 KB DOC)

Table S11 Amount of Low-Certainty Links at Different Degree Levels in Two Cambrian Food Webs (91 KB DOC)

Table S12 Niche Model Errors for Ten Modern Webs (62 KB DOC)

Table S13 Random-Beta Model Analysis Results for Two Cambrian Food Webs (52 KB DOC)

Table S14 Variability of Niche Model Mean Values and Model Errors across Six Runs of the Niche Model for Two Cambrian Food Webs (59 KB DOC)

## Abbreviations

C: connectance
CI: confidence interval
L: number of links in a food web
Ma: million years ago
ME: model error
S: number of species, taxa, or nodes in a food web
TL: trophic level
