# Supplementary material for: Compilation and Network Analyses of Cambrian Food Webs
Source: PLoS Biol. 2008 Apr 29;6(4):e102. doi: 10.1371/journal.pbio.0060102 (PMC2689700; doi:10.1371/journal.pbio.0060102)
Supplement: Table S1 — (225 KB DOC) [file pbio.0060102.st001.doc]

**Table S1.** Master taxa list for the Chengjiang Shale (138 taxa)

Taxa with potential synonyms indicated by * (see Table S3). Position is based on morphology and discussion in Hou *et al.* 2004 (3). References in “Evidence for Trophic Role” column denoted by numbers in parantheses as follows:

1. Brusca RC, Brusca GJ (1990) *Invertebrates* (Sinauer Press, Massachusetts).

2. Erwin DH, expert assessment.

3. Hou XG, Xiao M, Zhao J, Bergstrom J (2004) *Lethaia* 37:235-244.

4. Butterfield NJ (2002) *Paleobiology* 28:155-171.

5. Vannier J, Chen JY (2002) *Lethaia* 35:107-120.

6. Zhang XL, Han J, Zhang ZF, Liu HQ, Shu DG (2003) *Palaecology* 46:447-465.

7. Hou XG, Bergstrom J (1997) *Fossils and Strata*, 116 pp.

8. Briggs DEK, Erwin DH, Collier JF (1994) *The Fossils of the Burgess Shale* (Smithsonian Inst. Press, Washington).

9. Hou XG, Bergstrom J, Ahlberg PE (1995) *GFF* 117:163-183.

10. Chen JY, Waloszek D, Maas A (2004) *Lethaia* 37:3-20.

11. Shu D, Morris SC, Zhang ZF, Liu JN, Han J, Chen L, Zhang XL, Yasui K, Li Y (2003) *Science* 299:1380-1384.

12. Chen JY, Huang DY, Peng QQ, Chi HM, Want XQ, Feng M (2003) *Proc Nat Acad Sci USA* 100:8314-8318.

13. Shu DG, Conway Morris S, Han J, Zhang ZF, Liu JN (2002) *Nature* 430:422-428.

14. Bengston S, Hou XG (2001) *Acta Palaeon Polon* 46:1-22.

15. Zhang XL, Shu DG, Li Y, Han J (2001) *J Geol Soc Lon* 158:211-218.

16. Mallatt J, Chen JY (2003) *J Morph* 258:1-31.

| **Group** | **#** | **Taxon** | **Trophic Role** | **Position** | **Evidence for Trophic Role** |
| --- | --- | --- | --- | --- | --- |
| misc basal | 1 | phytoplankton | photosynthetic | pelagic, epifaunal | inferential |
|  | 2 | bacterioplankton | bacterial | pelagic, epifaunal | inferential |
|  | 3 | suspended organic matter | detrital | pelagic, epifaunal | inferential |
|  | 4 | benthic detritus | detrital | benthic, infaunal | inferential |
| Algae | 5 | Megaspirella houi | photosynthetic | epifaunal | phylogenetic conservation |
|  | 6 | Sinocylindra yunnanensis | photosynthetic | epifaunal | phylogenetic conservation |
|  | 7 | Yuknessia sp. | photosynthetic | epifaunal | phylogenetic conservation |
|  | 8 | Fuxianospira gyrata | photosynthetic | epifaunal | phylogenetic conservation |
| zooplankton | 9 | zooplankton | micro-planktivores | pelagic, epifaunal | inferential |
| Porifera | 10 | Allantospongia mica | filter-feeder | epifaunal, sessile | phylogenetic conservation: modern sponges largely feed on bacteria (1) |
|  | 11 | Choia xiaolantianensis | filter-feeder | epifaunal, infaunal?, sessile | as above |
|  | 12 | Choiaella radiata | filter-feeder | infaunal?, sessile | as above |
|  | 13 | Leptomitella confusa | filter-feeder | epifaunal, sessile | as above |
|  | 14 | Leptomitella conica | filter-feeder | epifaunal, sessile | as above |
|  | 15 | Leptomitus teretiusculus | filter-feeder | epifaunal, sessile | as above |
|  | 16 | Paraleptomitella dictyodroma | filter-feeder | epifaunal, sessile | as above |
|  | 17 | Paraleptomitella globosa | filter-feeder | epifaunal, sessile | as above |
|  | 18 | Quadrolaminella crassa | filter-feeder | epifaunal, sessile | as above |
|  | 19 | Sinoflabrum antiquum | filter-feeder | epifaunal, sessile | as above |
|  | 20 | Saetaspongia densa | filter-feeder | epifaunal, sessile | as above |
|  | 21 | Triticispongia diagonata | filter-feeder | epifaunal, sessile | as above |
| Cnidaria | 22 | Priscapennamaria angusta | micro-carnivores | epifaunal, sessile | phylogenetic conservation: true of all extant cnidarians (1) |
|  | 23 | Xianguangia sinica | micro-carnivores | epifaunal, sessile | as above |
| Ctenophora | 24 | Maotianoascus octonarius | carniverous | pelagic | phylogenetic conservation (1) |
|  | 25 | Sinoascus papillatus | carniverous | pelagic | phylogenetic conservation (1) |
| Hyolitha/Mollusca | 26 | Ambrolinevitus maximus | suspension-feeder | benthic | by analogy to *Haplophrentis* from Burgess Shale (2) |
|  | 27 | Ambrolinevitus ventricosus | suspension-feeder | benthic | by analogy to *Haplophrentis* (2) |
|  | 28 | Burithes yunanensis* | suspension-feeder | benthic | by analogy to *Haplophrentis* (2) |
|  | 29 | Linevitus optimus | suspension-feeder | benthic | by analogy to *Haplophrentis* (2) |
| Annelida ? | 30 | Polychaete? |  |  |  |
| Phoronida | 31 | Lotuba chengjiangensis |  |  |  |
| Brachiopoda | 32 | Heliomedusa orienta | suspension-feeder | epifaunal | phylogenetic conservation: all modern brachiopods are suspension feeders (1) |
|  | 33 | Diandongia pista | suspension-feeder | epifaunal | phylogenetic conservation (1) |
|  | 34 | Lingulella chengjiangensis | suspension-feeder | infaunal | phylogenetic conservation (1) |
|  | 35 | Lingulellotreta malongensis | suspension-feeder | infaunal | phylogenetic conservation (1) |
|  | 36 | Longtancunella chengiangensis | filter-feeder | epifaunal | phylogenetic conservation (3) |
| Onycophora/  Lobopoida | 37 | Cardiodictyon catenulum | predator | epibenthic | analogy to *Hallucigenia* |
|  | 38 | Hallucigenia fortis | predator (on sponges?) | epibenthic | ecological association (3) |
|  | 39 | Luolishania longicruris | predator (on sponges?) | epibenthic | ecological association (3) |
|  | 40 | Microdictyon sinicum | carnivore or microphagous | epibenthic |  |
|  | 41 | Onychodictyon ferox |  | epibenthic |  |
|  | 42 | Paucipodia inermis | predator | epibenthic | morphology (3) |
| Arthropoda | 43 | Acanthomeridion serratum |  | benthic | (3) |
|  | 44 | Almenia spinosa |  |  |  |
|  | 45 | Brachiocaris? yunnanensis |  |  | (3) |
|  | 46 | Canadaspis laevigata* | predator | epibenthic | gut morphology (4); (3) suggest deposit feeding |
|  | 47 | Chengjiangocaris longiformis | carnivore | epibenthic | morphological similarity to *Fuxianhuia* (3) |
|  | 48 | Cindarella eucalla | not a carnivore | benthic | (3) |
|  | 49 | Clypecaris pteroidea* | deposit-feeder | epibenthic/nektonic | sediment-filled gut (3) |
|  | 50 | Combinivalvula chengjiangensis |  |  |  |
|  | 51 | Comptalulata inflata |  |  |  |
|  | 52 | Comptalulata leshanensis |  |  |  |
|  | 53 | Dongshanocaris foliiformis |  |  |  |
|  | 54 | Ercaia minuscula |  |  |  |
|  | 55 | Forfexicaris valida | predator |  | appendage morphology (3) |
|  | 56 | Fortiforceps foliosa | carnivore | swimmer | appendage morphology (3) |
|  | 57 | Fuxianhuia protensa | carnivore | benthic | small grasping appendages (3) |
|  | 58 | Isoxys auritus |  | pelagic swimmer | appendages unknown (5) |
|  | 59 | Isoxys curviostratus |  | pelagic swimmer | as above |
|  | 60 | Isoxys paradoxus* |  | pelagic swimmer | as above |
|  | 61 | Jinagfengia multisegmentalis | predator | swimmer ? | similar morphology of ‘great appendage’ to *Yohoia* |
|  | 62 | Jiucunella paulula |  |  |  |
|  | 63 | Kuamaia lata | carnivore | benthic |  |
|  | 64 | Kuamaia muricata | carnivore? |  | limb morphology (3) |
|  | 65 | Kunmingella douvillei | uncertain | epibenthic/nektonic | (3) |
|  | 66 | Kunyuangella cheni |  |  |  |
|  | 67 | Leanchoilia illecebrosa* | predator/scavenger | epibenthic/nektonic | appendage morphology and structure of gut (3,4) |
|  | 68 | Liangshanella liangshanensis |  |  |  |
|  | 69 | Naraoia longicaudata | predator/scavenger | epibenthic | appendage morphology (5) |
|  | 70 | Naraoia sponosa | predator/scavenger | epibenthic | appendage morphology (5) |
|  | 71 | Occacaris oviformis | predator/scavenger? |  | appendage morphology (3) |
|  | 72 | Odaraia? eurypetala* | predator | nektonic | internal morphology (3,4) |
|  | 73 | Parapaleomerus sinensis |  |  |  |
|  | 74 | Pectocaris spatiosa |  | nektonic | (3) |
|  | 75 | Pisinnocaris subconigera* | carnivore | epibenthic | similar to *Fuxianhuia* |
|  | 76 | Primicaris laviformis |  | epibenthic/nektonic | (6) |
|  | 77 | Pseudoiulia cambrensis |  |  |  |
|  | 78 | Pygmaclypeatus daziensis |  |  |  |
|  | 79 | Retifacis abnormalis* | scavenger/predator | benthic | limb morphology (3) |
|  | 80 | Rhombiclavaria acantha |  |  |  |
|  | 81 | Saperion glumaceum | not carniverous | benthic | (3) |
|  | 82 | Sidneyia sinica | predator | benthic | limb morphology and gnathobases: Burgess specimens and (6) |
|  | 83 | Sinoburius lunaris | deposit-feeder | benthic | sediment-filled gut (7) |
|  | 84 | Skioldia aldna |  | benthic |  |
|  | 85 | Squamacula clypeata | deposit-feeder | benthic | sediment-filled gut (7) |
|  | 86 | Tanglangia longicaudata |  |  |  |
|  | 87 | Tsunydiscus aclis |  |  |  |
|  | 88 | Tsunyiella diandongensis |  |  |  |
|  | 89 | ?Tuzoia sinensis* |  |  |  |
|  | 90 | Urokodia aequlais |  |  |  |
|  | 91 | Waptia ovata | selective detritivore? | benthic | appendage morphology of Burgess specimens (3,8) |
|  | 92 | Wutingaspis tingi |  |  |  |
|  | 93 | Wutingella binodosa |  |  |  |
|  | 94 | Xandrella speculum | deposit-feeder | benthic | sediment in gut: (7) |
|  | 95 | Yunaannocaris megista | prob. like Canadaspis | active swimmer | overall morphology (3) |
|  | 96 | Eoreclichia intermedia | predator | benthic | limb and hypostome structure (3) |
|  | 97 | Kuanyangia pusulosa | predator | benthic | limb and hypostome structure (3) |
|  | 98 | Yunnanocephalus yunnanensis | predator? | benthic | similar to *Eoredlichia* (3) |
| Anomalocarididae | 99 | Amplectobelua symbrachiata | carniverous | nektonic | by phylogenetic conservation to *Anomalocaris* (3) |
|  | 100 | Anomalocaris saron | carniverous | nektonic | grasping appendage and mouthpart morphology (3) |
|  | 101 | Anomalocaris sp. |  |  | (9) |
|  | 102 | Cucumericrus decoratus | prob. predator | nektonic | by phylogenetic conservation to *Anomalocaris* (3) |
|  | 103 | Haikoucaris ercaiensis | carniverous | nektonic | by phylogenetic conservation to *Anomalocaris* (10) |
|  | 104 | Parapeytoia yunnanensis | carniverous | nektonic | grasping appendages (3) |
| Priapulida | 105 | Acosmia maotiania | deposit-feeder | infaunal | gut infilling (3) |
|  | 106 | Archotuba conoidalis* |  |  | assignment to priapulids uncertain |
|  | 107 | Cortnetis brevis |  |  |  |
|  | 108 | Palaeopriapulites parvus | deposit-feeder | infaunal | gut infilling (3) |
|  | 109 | Paraselkirkia jinningensis* | predator | infaunal | proboscis morphology similar to *Selkirkia* (3) |
|  | 110 | Protopriapulites haikouensis* | deposit-feeder | infaunal | no gut infilling (3) |
| Nematomorpha | 111 | Cricocosmia jinningensis | deposit-feeder | infaunal | gut infilling (3) |
|  | 112 | Maotianshania cylindrica | deposit-feeder | infaunal | gut infilling (3) |
|  | 113 | Palaeoxcolex sinensis | predator? | infaunal? | no gut infilling (3) |
| Echinodermata | 114 | Eldonia eumorpha* |  | pelagic | (3) |
|  | 115 | Rotadiscus grandis |  | pelagic? | (3) |
| Chaetognatha | 116 | Eognathacanta ercainella | predator | planktonic | phylogenetic conservation (1) |
| Chordata | 117 | Cathymyrus diadexus |  |  |  |
|  | 118 | ?Cathymyrus haiokouensis |  |  |  |
|  | 119 | Myllokunmingia fengjiaoa* |  | nektonic | (11) |
|  | 120 | Shankouclava shankouense |  | benthic | tunicate (12) |
|  | 121 | ?Zhongxiniscus intermedius |  | epi-benthic or burrower | (11) |
| Vetulicolia | 122 | Didazoon haoae |  |  |  |
|  | 123 | Pomatrum ventralis |  |  |  |
|  | 124 | Vetulicola cuneata* | deposit-feeder | nekto-benthic | possible filter-feeder, based on gill openings in pharynx (3) |
|  | 125 | Xidazoon stephanus |  |  |  |
|  | 126 | Dianchicystis jianshanensis | filter-feeder | semi-sessile |  |
| Vetulocystida | 127 | Vetulocystis catenata | filter-feeder | semi-sessile | mouthpart morphology (13) |
| problematica | 128 | Allonnia phrixothrix* |  | benthic | Chancellorid, possible sponge-like habitat (14) |
|  | 129 | Banffia confusa* | deposit-feeder | nekto-benthic | possible filter-feeder, morpho-logically similar to Vetulicola (3) |
|  | 130 | Batofascuculus ramificans |  |  |  |
|  | 131 | Cambrotentacus sanwiua* | suspension feeder? | benthic | overall morphology (15) |
|  | 132 | Dinomischus venustus | suspension feeder | benthic | sessile habitat & morphology (2,3) |
|  | 133 | Facivermis yunnanicus |  | burrower? |  |
|  | 134 | Jiucunia petalina |  | benthic | sponge? morphology; possibly colonial (3) |
|  | 135 | Maanshania crusticeps |  |  |  |
|  | 136 | Parvulonoda dubia |  | benthic | sponge or algae? |
|  | 137 | Phlogites longus* |  |  |  |
|  | 138 | Yunnanozoon lividum* | suspension feeder |  | branchial arch morphology (16,3) |
