# Supplementary material for: Compilation and Network Analyses of Cambrian Food Webs
Source: PLoS Biol. 2008 Apr 29;6(4):e102. doi: 10.1371/journal.pbio.0060102 (PMC2689700; doi:10.1371/journal.pbio.0060102)
Supplement: Table S2 — (289 KB DOC) [file pbio.0060102.st002.doc]

**Table S2.** Master taxa list for the Burgess Shale (171 taxa)

References in “Evidence for Trophic Role” column denoted by numbers in parantheses as follows:

1. Brusca RC, Brusca GJ (1990) *Invertebrates* (Sinauer Press, Massachusetts).

2. Briggs DEK, Erwin DH, Collier JF (1994) *The Fossils of the Burgess Shale* (Smithsonian Inst. Press, Washington).

3. Butterfield NJ (2002) *Paleobiology* 28:155-171.

4. Garcia-Bellido DC, Collins DH (2006) *Candain Journal of Earth Sceinces* 43:721-742.

5. Pers. com. to DHE by Nigel Hughes (2004).

6. Fortey RA, Owens RM (1999) *Paleontology* 42:429-465.

| **Group** | **#** | **Taxon** | **Trophic Role** | **Position** | **Evidence for Trophic Role** |
| --- | --- | --- | --- | --- | --- |
| misc basal | 1 | phytoplankton | photosynthetic | pelagic, epifaunal | inferential |
|  | 2 | bacterioplankton | bacterial | pelagic, epifaunal | inferential |
|  | 3 | suspended organic matter | detrital | pelagic, epifaunal | inferential |
|  | 4 | benthic detritus | detrital | benthic, infaunal | inferential |
| Algae | 5 | Marpolia spissa | photosynthetic | epifaunal | modern analogs |
|  | 6 | Morania confluens | photosynthetic | epifaunal | as above |
|  | 7 | Morania elongata | photosynthetic | epifaunal | as above |
|  | 8 | Morania fragmenta | photosynthetic | epifaunal | as above |
|  | 9 | Morania? frondosa | photosynthetic | epifaunal | as above |
|  | 10 | Morania? globosa | photosynthetic | epifaunal | as above |
|  | 11 | Morania parasitica | photosynthetic | epifaunal | as above |
|  | 12 | Morania? reticulata | photosynthetic | epifaunal | as above |
|  | 13 | Margaretia dorus | photosynthetic | epifaunal | as above |
|  | 14 | Yuknessia simplex | photosynthetic | epifaunal | as above |
|  | 15 | Bosworthia gyges | photosynthetic | epifaunal | as above |
|  | 16 | Bosworthia simulans | photosynthetic | epifaunal | as above |
|  | 17 | Dalyia nitens | photosynthetic | epifaunal | as above |
|  | 18 | Dalyia racemata | photosynthetic | epifaunal | as above |
|  | 19 | Wahpia mimica | photosynthetic | epifaunal | as above |
|  | 20 | Wahpia virgata | photosynthetic | epifaunal | as above |
|  | 21 | Waputikia ramosa | photosynthetic | epifaunal | as above |
|  | 22 | Sphaerocodium cambria | photosynthetic | epifaunal | as above |
|  | 23 | Sphaerocodium praecursor | photosynthetic | epifaunal | as above |
|  | 24 | Dictyphycus gracilis | photosynthetic | epifaunal | as above |
| zooplankton | 25 | zooplankton | microplanktivores | pelagic, epifaunal | inferential |
| Porifera | 26 | Capsospongia undulata | filter-feeders | epifaunal, sessile | phylogenetic conservation: modern sponges largely feed on bacteria (1) |
|  | 27 | Choia carteri | filter-feeders | epifaunal, sessile | as above |
|  | 28 | Choia ridleyi | filter-feeders | epifaunal, sessile | as above |
|  | 29 | Crumillospongia biporosa | filter-feeders | epifaunal, sessile | as above |
|  | 30 | Crumillospongia frondosa | filter-feeders | epifaunal, sessile | as above |
|  | 31 | Falospongia falata | filter-feeders | epifaunal, sessile | as above |
|  | 32 | Fieldospongia bellilineata | filter-feeders | epifaunal, sessile | as above |
|  | 33 | Halichondrites elissa | filter-feeders | epifaunal, sessile | as above |
|  | 34 | Hamptonia bowerbanki | filter-feeders | epifaunal, sessile | as above |
|  | 35 | Hazelia conferta | filter-feeders | epifaunal, sessile | as above |
|  | 36 | Hazelia crateria | filter-feeders | epifaunal, sessile | as above |
|  | 37 | Hazelia delicatula | filter-feeders | epifaunal, sessile | as above |
|  | 38 | Hazelia dignata | filter-feeders | epifaunal, sessile | as above |
|  | 39 | Hazelia grandis | filter-feeders | epifaunal, sessile | as above |
|  | 40 | Hazelia luteria | filter-feeders | epifaunal, sessile | as above |
|  | 41 | Hazelia nodulifera | filter-feeders | epifaunal, sessile | as above |
|  | 42 | Hazelia obscura | filter-feeders | epifaunal, sessile | as above |
|  | 43 | Hazelia palmata | filter-feeders | epifaunal, sessile | as above |
|  | 44 | Leptomitus lineatus | filter-feeders | epifaunal, sessile | as above |
|  | 45 | Moleculopina mammilata | filter-feeders | epifaunal, sessile | as above |
|  | 46 | Pirania muricata | filter-feeders | epifaunal, sessile | as above |
|  | 47 | Sentinelia draco | filter-feeders | epifaunal, sessile | as above |
|  | 48 | Takakkawia lineata | filter-feeders | epifaunal, sessile | as above |
|  | 49 | Vauxia bellula | filter-feeders | epifaunal, sessile | as above |
|  | 50 | Vauxia densa | filter-feeders | epifaunal, sessile | as above |
|  | 51 | Vauxia gracilenta | filter-feeders | epifaunal, sessile | as above |
|  | 52 | Vauxia venata | filter-feeders | epifaunal, sessile | as above |
|  | 53 | Wapkia grandis | filter-feeders | epifaunal, sessile | as above |
|  | 54 | Diagoniella hindei | filter-feeders | epifaunal, sessile | as above |
|  | 55 | Protospongia hicksi | filter-feeders | epifaunal, sessile | as above |
|  | 56 | Stephanospongia magnipora | filter-feeders | epifaunal, sessile | as above |
|  | 57 | Canistrumella alternata | filter-feeders | epifaunal, sessile | as above |
|  | 58 | Eiffelia globosa | filter-feeders | epifaunal, sessile | as above |
|  |  |  |  |  |  |
| Cnidaria (?) | 59 | Cambrorhytium fragilis | micro-carnivores | epifaunal, sessile | phylogenetic conservation: true of all extant cnidarians (1) |
|  | 60 | Cambrorhytium major | micro-carnivores | epifaunal, sessile | as above |
|  | 61 | Gelenopteron tentaculatum | micro-carnivores | epifaunal, sessile | as above |
|  | 62 | Mackenzia costalis | micro-carnivores | epifaunal, sessile | as above |
|  | 63 | Thaumaptilon walcotti | micro-carnivores | epifaunal, sessile | as above |
| Ctenophora | 64 | Fasciculus vesanus | carniverous | pelagic | phylogenetic conservation (1) |
| Hyolitha/Mollusca (?) | 65 | Haplophrentis carinatus | suspension-feeder | benthic | (2) |
|  | 66 | Scenella amii |  |  |  |
| Annelida | 67 | Burgessochaeta setigera | select. deposit feeder | burrowing | lack of sediment in gut; head morphology (2) |
|  | 68 | Canadia spinosa | carnivor/scavenger | epifaunal | head and limb morphology |
|  | 69 | Insolicorypha psygma |  | pelagic? | morphology |
|  | 70 | Peronochaeta dubia | scavenging? | infaunal | lack of sed. in gut; infaunal nature & no predatory specializations |
|  | 71 | Stephanoscolex argutus | scavenging? | epi/infaunal | no sed. in gut eliminates deposit feeding; no pred. specializations |
|  | 72 | Wiwaxia corrugata |  |  |  |
| Brachiopoda | 73 | Acrothyra gregaria | suspension-feeders | epifaunal, sessile | modern analogy: all modern brachiopods (1) |
|  | 74 | Lingulella waptaensis | suspension-feeders | epifaunal, sessile | as above |
|  | 75 | Micromitra burgessensis | suspension-feeders | epifaunal, sessile | as above |
|  | 76 | Paterina zenobia | suspension-feeders | epifaunal, sessile | as above |
|  | 77 | Diraphora bellicostata | suspension-feeders | epifaunal, sessile | as above |
|  | 78 | Nisusia burgessensis | suspension-feeders | epifaunal, sessile | as above |
| Onycophora/Lobopodia | 79 | Aysheaia pedunculata | predator | epibenthic | assoc. w/ sponges |
|  | 80 | Hallucigenia sparsa | predator | epibenthic | limb morphology; by analogy w/ *Aysheasia* |
| Arthropoda | 81 | Actaeus armatus |  |  |  |
|  | 82 | Alalcomenaeus cambricus |  |  |  |
|  | 83 | Aluta? sp. |  |  |  |
|  | 84 | Branchiocaris pretiosa | scavenger/feeding on sessile animals | epibenthic | no eyes; limb morphoology |
|  | 85 | Burgessia bella |  |  |  |
|  | 86 | Canadaspis ovalis | carnivore | epifaunal | gut morphology (3) |
|  | 87 | Canadaspis perfecta | carnivore | epifaunal | gut morphology (3) |
|  | 88 | Carnarvonia venosa |  |  |  |
|  | 89 | Emeraldella bocki | predator | epifaunal | limb morphology (2) |
|  | 90 | Habelia brevicauda | scavenging? | epifaunal | no evidence of deposit feeding; apparently unspecialized appendages |
|  | 91 | Habelia optata | scavenging? | epifaunal | as above |
|  | 92 | Helmetia expansa | filter-feeder? | nektonic | as above |
|  | 93 | Houghtonites gracilis |  |  |  |
|  | 94 | Isoxys acutangulus |  | pelagic swimmer | morphology |
|  | 95 | Isoxys longissimus |  | pelagic swimmer | morphology |
|  | 96 | Leanchoilia superlata | predator | epifaunal | gut morphology (3); limb morphology (2) |
|  | 97 | Marella splendens | suspension feeding zooplankter | epifaunal | appendage morphology (4) |
|  | 98 | Molaria spinifera |  |  |  |
|  | 99 | Mollisonia symmetrica |  |  |  |
|  | 100 | Naraoia compacta | predator | epifaunal | spines on limbs (2) |
|  | 101 | Odaraia alata | predator | pelagic swimmer | gut morphology (3) |
|  | 102 | Perspicaris dictynna | predator |  | gut morphology (3) |
|  | 103 | Perspicaris recondita | predator | pelagic | rarity and morphology; pelagic nature; gut morphology (3) |
|  | 104 | Priscansermarius barnetti |  |  |  |
|  | 105 | Sanctacaris uncata |  |  |  |
|  | 106 | Sarotrocerus oblita |  | nektonic |  |
|  | 107 | Sidneyia inexpectans | predator | epifaunal | limb and gut morphology (3,2) |
|  | 108 | Skania fragilis |  |  |  |
|  | 109 | Tegopelte gigas |  |  |  |
|  | 110 | Thelxiope palaeothallasia |  |  |  |
|  | 111 | Tuzoia burgessensis |  |  |  |
|  | 112 | Tuzoia canadensis |  |  |  |
|  | 113 | Tuzoia? parva |  |  |  |
|  | 114 | Tuzoia praemorsa |  |  |  |
|  | 115 | Tuzoia retifera |  |  |  |
|  | 116 | Waptia fieldensis |  | epifaunal | morphology |
|  | 117 | Yohoia tenuis | predator/scavenger | epifaunal | limb morphology (2) |
|  | 118 | Chancia palliseri |  |  |  |
|  | 119 | Ehmaniella burgessensis |  |  |  |
|  | 120 | Ehmaniella waptaensis |  |  |  |
|  | 121 | Elrathia permulta |  |  |  |
|  | 122 | Elrathina brevifrons |  |  |  |
|  | 123 | Elrathina crodillerae |  |  |  |
|  | 124 | Hanburia gloriosa |  |  |  |
|  | 125 | Kootenia burgessensis |  |  |  |
|  | 126 | Olenoides serratus | predator | epifaunal | hypostome structure; gnathobases and limb morphology (5,6) |
|  | 127 | Oryctocephalus burgessensis | predator/scavenger | epifaunal | as above |
|  | 128 | Oryctocephalus matthewi | predator/scavenger | epifaunal | as above |
|  | 129 | Oryctoecphalus reynoldsi | predator/scavenger | epifaunal | as above |
|  | 130 | Pagetia bootes | filter/susp. feeder | pelagic | as above |
|  | 131 | Parkaspis decamera | predator/scavenger | epifaunal | as above |
|  | 132 | Peronopsis montis | filter/susp. feeder | pelagic | as above |
|  | 133 | Ptychagnostus praecurrens | filter/susp. feeder | pelagic | as above |
|  | 134 | Spencella sp indet |  |  |  |
| Anomalocarididae | 135 | Anomalocaris canadensis | large predator | epifaunal | mouthpart and limb morphology (2) |
|  | 136 | Laggania nathorsti | large predator | epifaunal | as above |
| problematica (Arthropoda) | 137 | Amiella ornata |  |  |  |
|  | 138 | Hurdia dentata | predator? | epifaunal |  |
|  | 139 | Hurdia triangulata | predator? | epifaunal |  |
|  | 140 | Hurdia vicroria | predator? | epifuanal |  |
|  | 141 | Opabinia regalis | predator | nektonic | gut morphology; limb morphology (3) |
| Priapulida | 142 | Ancalagon minor | predator | burrowing | modern analogs, morphology (2) |
|  | 143 | Fieldia lanceolata | sediment-feeder | burrowing | gut contents; as above |
|  | 144 | Louisella pedunculata | predator |  | modern analogs, morphology (2) |
|  | 145 | Ottoia prolifica | predator | burrowing | gut contents |
|  | 146 | Selkirkia columbia | predator | burrowing | as above |
| Echinodermata (?) | 147 | Eldonia ludwigi | filter-feeders | epifaunal | ecological position and modern analogs |
|  | 148 | Echmatocrinus brachiatus | filter-feeders | epifaunal | as above |
|  | 149 | Gogia radiata | filter-feeders | epifaunal | as above |
|  | 150 | Walcottidiscus magister | filter-feeders | epifaunal | as above |
|  | 151 | Walcottidiscus typicalis | filter-feeders | epifaunal | as above |
| Hemichordata | 152 | Chaunograptus scandens | filter-feeders | epifaunal |  |
|  | 153 | "Ottoia" tenuis | filter-feeders | epifaunal |  |
| Chordata | 154 | Metaspriginna walcotti |  |  |  |
|  | 155 | Pikaia gracilens | filter-feeders | epifaunal | modern analog |
| problematica | 156 | Amiskwia sagittiformis |  |  |  |
|  | 157 | Banffia constricta |  |  |  |
|  | 158 | Chancelloria eros |  |  |  |
|  | 159 | Dinomischus isolatus |  |  |  |
|  | 160 | Nectocaris pteryx | predator | nektonic | overall morphology (2) |
|  | 161 | Odontogriphus omalus |  |  |  |
|  | 162 | Oesia disjuncta |  |  |  |
|  | 163 | Platydendron ovale |  |  |  |
|  | 164 | Plenocaris plena |  |  |  |
|  | 165 | Pollingeria grandis |  |  |  |
|  | 166 | Portalia mira |  |  |  |
|  | 167 | Proboscicaris agnosta |  |  |  |
|  | 168 | Probosicaris ingens |  |  |  |
|  | 169 | Probosicaris obtusa |  |  |  |
|  | 170 | Scolecofurca rara |  |  |  |
|  | 171 | Worthenella cambria |  |  |  |
