# Supplementary material for: Compilation and Network Analyses of Cambrian Food Webs
Source: PLoS Biol. 2008 Apr 29;6(4):e102. doi: 10.1371/journal.pbio.0060102 (PMC2689700; doi:10.1371/journal.pbio.0060102)
Supplement: Table S3 — (73 KB DOC) [file pbio.0060102.st003.doc]

**Table S3.** Synonyms for Chengjiang Shale taxa

Questionable synonyms indicated by (?).

**References**

1. Huo HL, Hu SX, Chen LZ, Zhang SS, Tao YH (1999) *Early Cambrian Chengjiang Fauna from Kunming Region, China* (Yunnan Science and Technology Press, Kunming).

2. Chen JY, Zhou GQ (1997) *Bull Nat Mus Nat Sci* 10:11-105.

3. Hou HL (1997) *Bradoriid arthropods from the Lower Cambrian of Southwest China* (Unpublished Ph.D. Dissertation, Uppsala University, Sweden).

4. Shu DG (1990) *Cambrian and Lower Ordovian Bradoriida from Zhejiang, Hunan and Shanxi Provinces* (Worthwest University Press, Xi’an). [In Chinese, with English summary]

5. Sun WG, Huo XG (1987) *Acta Palaeon Sin* 26:257-271.

6. Jannussen D, Steiner M, Zhu MY (2002) *J Paleon* 76:596-606.

7. Zhang XL, Shu DG, Li Y, Han J (2001) *J Geol Soc Lon* 158:211-218.

8. Chen JY, Huang DY, Li CW (1999) *Nature* 402:518-522.

9. Shu D, Morris SC, Zhang ZF, Liu JN, Han J, Chen L, Zhang XL, Yasui K, Li Y (2003) *Science* 299:1380-1384.

| **#** | **Taxon** | **Synonym** | **Citation** |
| --- | --- | --- | --- |
| 28 | Burithes yunanensis | Glossolites magnus (?) | (1) |
| 46 | Canadaspis laevigata | Canadaspis eucallus | (2) |
|  |  | Perspicaris? sp. | (3) |
|  |  | Yiliangocaris ellipticus (?) | (1) |
| 49 | Clypecaris pteroidea | Ercaicunia multinodosa (?) |  |
| 60 | Isoxys paradoxus | Isoxys elongatus (?) | (1) |
| 67 | Leanchoilia illecebrosa | Dianchia mirabilis | (1) |
|  |  | Leanhoilia asiatica | (1) |
|  |  | Yohoia sinensis | (1) |
|  |  | Zhongiania speciose (?) | (1) |
|  |  | Apiocephalus elegans (?) | (1) |
| 72 | Odaraia? eurypetala | Glossocaris osulatus (?) | (1) |
| 75 | Pisinnocaris subconigera | Jinshania furcatus (?) | (1) |
| 79 | Retifacis abnormalis | Retifacis longispinus (?) | (1) |
|  |  | Tuzoia sp. | (4) |
| 89 | Tuzoia sinensis | Tuzoia limba (?) | (4) |
| 106 | Archotuba conoidalis | Selkirkia elongata (?) |  |
| 109 | Paraselkirkia jinningensis | Selkirkia sinica (?) | (1) |
| 110 | Protopriapulites haikouensis | Sicyophorus rarus | (1) |
| 114 | Eldonia eumorpha | Yunnanomedusa eleganta | (5) |
| 119 | Myllokunmingia fengjiaoa | Hiakouicthus ercaicunensis | (1) |
| 124 | Vetulicola cuneata | Vetulicola rectangulata (?) | (1) |
| 128 | Allonnia phrixothrix | Allonnia junyuania | (6) |
| 129 | Banffia confusa | Heteromorphus longicaudatus (?) | (1) |
| 131 | Cambrotentacus sanwiua | Cotyledion tylodes (?) | (7) |
| 137 | Phlogites longus | Phlogites brevis | (1) |
|  |  | Calathites spinalis (?) | (1) |
| 138 | Yunnanozoon lividum | Haikouella lanceolata (?) | (8) |
|  |  | Haikouella jianshanensis | (9) |
