# Supplementary material for: Compilation and Network Analyses of Cambrian Food Webs
Source: PLoS Biol. 2008 Apr 29;6(4):e102. doi: 10.1371/journal.pbio.0060102 (PMC2689700; doi:10.1371/journal.pbio.0060102)
Supplement: Table S4 — (84 KB DOC) [file pbio.0060102.st004.doc]

**Table S4.** Chengjiang Shale taxa not included in Chengjiang food-web dataset

53 of 138 taxa are excluded from the final Chengjiang food-web dataset (Table S6) due to critically incomplete trophic information. Table S4a: 17 animal species that lack resources and are also listed as the resource for other animal taxa. These species, and the 34 links to them, are excluded. Table S4b: 32 animal species that lack resources, but are not listed as the resources for other taxa. Table S4c: 4 basal species, the four algae species, which lack consumers.

| **Table S4a. Animal taxa with no resources with consumers** | | |
| --- | --- | --- |
|  |  |  |
| **#** | **Taxon** | **# Links** |
| 30 | polychaete? | 1 |
| 43 | Acanthomeridion serratum | 1 |
| 48 | Cindarella eucalla | 1 |
| 50 | Combinivalvula chengjiangensis | 1 |
| 53 | Dongshanocaris foliiformis | 3 |
| 55 | Forfexicaris valida | 2 |
| 60 | Isoxys paradoxus | 4 |
| 68 | Liangshanella liangshanensis | 2 |
| 73 | Parapaleomerus sinensis | 4 |
| 74 | Pectocaris spatiosa | 3 |
| 76 | Primicaris laviformis | 5 |
| 77 | Pseudoiulia cambrensis | 1 |
| 84 | Skioldia aldna | 1 |
| 90 | Urokodia aequlais | 2 |
| 106 | Archotuba conoidalis | 1 |
| 107 | Cortnetis brevis | 1 |
| 113 | Palaeoxcolex sinensis | 1 |
|  |  |  |
| **Table S4b. Animal taxa with no resources and no consumers** | | |
|  |  | |
| **#** | **Taxon** | |
| 37 | Cardiodictyon catenulum | |
| 44 | Almenia spinosa | |
| 51 | Comptalulata inflata | |
| 52 | Comptalulata leshanensis | |
| 54 | Ercaia minuscula | |
| 58 | Isoxys auritus | |
| 59 | Isoxys curviostratus | |
| 62 | Jiucunella paulula | |
| 64 | Kuamaia muricata | |
| 66 | Kunyuangella cheni | |
| 71 | Occacaris oviformis | |
| 78 | Pygmaclypeatus daziensis | |
| 80 | Rhombiclavaria acantha | |
| 86 | Tanglangia longicaudata | |
| 87 | Tsunydiscus aclis | |
| 88 | Tsunyiella diandongensis | |
| 89 | ?Tuzoia sinensis | |
| 92 | Wutingaspis tingi | |
| 93 | Wutingella binodosa | |
| 101 | Anomalocaris sp. | |
| 117 | Cathymyrus diadexus | |
| 118 | ?Cathymyrus haiokouensis | |
| 119 | Myllokunmingia fengjiaoa | |
| 120 | Shankouclava shankouense | |
| 121 | ?Zhongxiniscus intermedius | |
| 122 | Didazoon haoae | |
| 123 | Pomatrum ventralis | |
| 125 | Xidazoon stephanus | |
| 128 | Allonnia phrixothrix | |
| 130 | Batofascuculus ramificans | |
| 135 | Maanshania crusticeps | |
| 137 | Phlogites longus | |
|  |  | |
| **Table S4c. Basal taxa that lack consumers** | | |
|  |  | |
| **#** | **Taxon** | |
| 5 | Megaspirella houi | |
| 6 | Sinocylindra yunnanensis | |
| 7 | Yuknessia sp. | |
| 8 | Fuxianospira gyrata | |
