# Supplementary material for: Compilation and Network Analyses of Cambrian Food Webs
Source: PLoS Biol. 2008 Apr 29;6(4):e102. doi: 10.1371/journal.pbio.0060102 (PMC2689700; doi:10.1371/journal.pbio.0060102)
Supplement: Table S5 — (59 KB DOC) [file pbio.0060102.st005.doc]

**Table S5.** Burgess Shale taxa not included in Burgess food-web dataset

29 of 171 taxa are excluded from the final Burgess food-web dataset (Table S7) due to critcially incomplete trophic information. Table 5a: 13 animal species that lack resources and are also listed as the resource for other animal species. These species, and the 86 links to them, are excluded. Table 5b: 16 animal species that lack resources, but are not listed as the resources for other taxa.

| **Table 5a. Animal taxa with no resources with consumers** | | |
| --- | --- | --- |
| **#** | **Taxon** | **# Links** |
| 69 | Insolicorypha psygma | 29 |
| 84 | Branchiocaris pretiosa | 7 |
| 88 | Carnarvonia venosa | 2 |
| 94 | Isoxys acutangulus | 4 |
| 95 | Isoxys longissimus | 4 |
| 106 | Sarotrocerus oblita | 10 |
| 109 | Tegopelte gigas | 4 |
| 124 | Hanburia gloriosa | 4 |
| 125 | Kootenia burgessensis | 4 |
| 134 | Spencella sp indet | 4 |
| 154 | Metaspriginna walcotti | 6 |
| 156 | Amiskwia sagittiformis | 6 |
| 167 | Proboscicaris agnosta | 2 |
|  |  |  |
| **Table 5b. Animal taxa with no resources and no consumers** | | |
| **#** | **Taxon** | |
| 81 | Actaeus armatus | |
| 82 | Alalcomenaeus cambricus | |
| 93 | Houghtonites gracilis | |
| 99 | Mollisonia symmetrica | |
| 108 | Skania fragilis | |
| 110 | Thelxiope palaeothallasia | |
| 137 | Amiella ornata | |
| 157 | Banffia constricta | |
| 161 | Odontogriphus omalus | |
| 163 | Platydendron ovale | |
| 164 | Plenocaris plena | |
| 165 | Pollingeria grandis | |
| 168 | Probosicaris ingens | |
| 169 | Probosicaris obtusa | |
| 170 | Scolecofurca rara | |
| 171 | Worthenella cambria | |
