# Supplementary material for: Compilation and Network Analyses of Cambrian Food Webs
Source: PLoS Biol. 2008 Apr 29;6(4):e102. doi: 10.1371/journal.pbio.0060102 (PMC2689700; doi:10.1371/journal.pbio.0060102)
Supplement: Table S6 — (805 KB DOC) [file pbio.0060102.st006.doc]

**Table S6.** Chengjiang Shale food-web data

Food-web data, with corresponding taxa names and numbers, for the Chengjiang Shale. The numbers correspond to species numbers in the master taxa list (Table S1). * indicates species with possible synonymous identifications (Table S3).

| **Con. #** | **Consumer sp.** | **Res. #** | **Resource sp.** | **Certainty** |
| --- | --- | --- | --- | --- |
| 1 | phytoplankton |  |  |  |
| 2 | bacterioplankton |  |  |  |
| 3 | suspended organic matter |  |  |  |
| 4 | benthic detritus |  |  |  |
| 9 | zooplankton | 1 | phytoplankton | 3 |
|  |  | 2 | bacterioplankton | 3 |
| 10 | Allantospongia mica | 2 | bacterioplankton | 2 |
| 11 | Choia xiaolantianensis | 2 | bacterioplankton | 2 |
| 12 | Choiaella radiata | 2 | bacterioplankton | 2 |
| 13 | Leptomitella confusa | 2 | bacterioplankton | 2 |
| 14 | Leptomitella conica | 2 | bacterioplankton | 2 |
| 15 | Leptomitus teretiusculus | 2 | bacterioplankton | 2 |
| 16 | Paraleptomitella dictyodroma | 2 | bacterioplankton | 2 |
| 17 | Paraleptomitella globosa | 2 | bacterioplankton | 2 |
| 18 | Quadrolaminella crassa | 2 | bacterioplankton | 2 |
| 19 | Sinoflabrum antiquum | 2 | bacterioplankton | 2 |
| 20 | Saetaspongia densa | 2 | bacterioplankton | 2 |
| 21 | Triticispongia diagonata | 2 | bacterioplankton | 2 |
| 22 | Priscapennamaria angusta | 9 | zooplankton | 1 |
| 23 | Xianguangia sinica | 9 | zooplankton | 1 |
| 24 | Maotianoascus octonarius | 9 | zooplankton | 2 |
|  |  | 22 | Priscapennamaria angusta | 1 |
|  |  | 23 | Xianguangia sinica | 1 |
|  |  | 24 | Maotianoascus octonarius | 1 |
|  |  | 25 | Sinoascus papillatus | 1 |
| 25 | Sinoascus papillatus | 9 | zooplankton | 2 |
|  |  | 22 | Priscapennamaria angusta | 1 |
|  |  | 23 | Xianguangia sinica | 1 |
|  |  | 24 | Maotianoascus octonarius | 1 |
|  |  | 25 | Sinoascus papillatus | 1 |
| 26 | Ambrolinevitus maximus | 3 | SOM | 1 |
|  |  | 9 | zooplankton | 1 |
| 27 | Ambrolinevitus ventricosus | 3 | SOM | 1 |
|  |  | 9 | zooplankton | 1 |
| 28 | Burithes yunanensis* | 3 | SOM | 1 |
|  |  | 9 | zooplankton | 1 |
| 29 | Linevitus optimus | 3 | SOM | 1 |
|  |  | 9 | zooplankton | 1 |
| 31 | Lotuba chengjiangensis | 2 | bacterioplankton | 2 |
|  |  | 3 | SOM | 1 |
| 32 | Heliomedusa orienta | 2 | bacterioplankton | 2 |
|  |  | 3 | SOM | 1 |
| 33 | Diandongia pista | 2 | bacterioplankton | 2 |
|  |  | 3 | SOM | 1 |
| 34 | Lingulella chengjiangensis | 2 | bacterioplankton | 2 |
|  |  | 3 | SOM | 1 |
| 35 | Lingulellotreta malongensis | 2 | bacterioplankton | 2 |
|  |  | 3 | SOM | 1 |
| 36 | Longtancunella chengiangensis | 2 | bacterioplankton | 2 |
|  |  | 3 | SOM | 1 |
| 38 | Hallucigenia fortis | 10 | Allantospongia mica | 1 |
|  |  | 11 | Choia xiaolantianensis | 1 |
|  |  | 12 | Choiaella radiata | 1 |
|  |  | 13 | Leptomitella confusa | 1 |
|  |  | 14 | Leptomitella conica | 1 |
|  |  | 15 | Leptomitus teretiusculus | 1 |
|  |  | 16 | Paraleptomitella dictyodroma | 1 |
|  |  | 17 | Paraleptomitella globosa | 1 |
|  |  | 18 | Quadrolaminella crassa | 1 |
|  |  | 19 | Sinoflabrum antiquum | 1 |
|  |  | 20 | Saetaspongia densa | 1 |
|  |  | 21 | Triticispongia diagonata | 1 |
| 39 | Luolishania longicruris | 10 | Allantospongia mica | 1 |
|  |  | 11 | Choia xiaolantianensis | 1 |
|  |  | 12 | Choiaella radiata | 1 |
|  |  | 13 | Leptomitella confusa | 1 |
|  |  | 14 | Leptomitella conica | 1 |
|  |  | 15 | Leptomitus teretiusculus | 1 |
|  |  | 16 | Paraleptomitella dictyodroma | 1 |
|  |  | 17 | Paraleptomitella globosa | 1 |
|  |  | 18 | Quadrolaminella crassa | 1 |
|  |  | 19 | Sinoflabrum antiquum | 1 |
|  |  | 20 | Saetaspongia densa | 1 |
|  |  | 21 | Triticispongia diagonata | 1 |
| 40 | Microdictyon sinicum | 10 | Allantospongia mica | 1 |
|  |  | 11 | Choia xiaolantianensis | 1 |
|  |  | 12 | Choiaella radiata | 1 |
|  |  | 13 | Leptomitella confusa | 1 |
|  |  | 14 | Leptomitella conica | 1 |
|  |  | 15 | Leptomitus teretiusculus | 1 |
|  |  | 16 | Paraleptomitella dictyodroma | 1 |
|  |  | 17 | Paraleptomitella globosa | 1 |
|  |  | 18 | Quadrolaminella crassa | 1 |
|  |  | 19 | Sinoflabrum antiquum | 1 |
|  |  | 20 | Saetaspongia densa | 1 |
|  |  | 21 | Triticispongia diagonata | 1 |
|  |  | 114 | Eldonia eumorpha* | 1 |
| 41 | Onychodictyon ferox | 10 | Allantospongia mica | 1 |
|  |  | 11 | Choia xiaolantianensis | 1 |
|  |  | 12 | Choiaella radiata | 1 |
|  |  | 13 | Leptomitella confusa | 1 |
|  |  | 14 | Leptomitella conica | 1 |
|  |  | 15 | Leptomitus teretiusculus | 1 |
|  |  | 16 | Paraleptomitella dictyodroma | 1 |
|  |  | 17 | Paraleptomitella globosa | 1 |
|  |  | 18 | Quadrolaminella crassa | 1 |
|  |  | 19 | Sinoflabrum antiquum | 1 |
|  |  | 20 | Saetaspongia densa | 1 |
|  |  | 21 | Triticispongia diagonata | 1 |
| 42 | Paucipodia inermis | 114 | Eldonia eumorpha* | 2 |
| 45 | Brachiocaris? yunnanensis | 9 | zooplankton | 2 |
| 46 | Canadaspis laevigata* | 4 | detritus | 2 |
| 47 | Chengjiangocaris longiformis | 45 | Brachiocaris? yunnanensis | 1 |
|  |  | 46 | Canadaspis laevigata* | 1 |
|  |  | 47 | Chengjiangocaris longiformis | 2 |
|  |  | 56 | Fortiforceps foliosa | 1 |
|  |  | 57 | Fuxianhuia protensa | 2 |
|  |  | 61 | Jinagfengia multisegmentalis | 1 |
|  |  | 63 | Kuamaia lata | 2 |
|  |  | 67 | Leanchoilia illecebrosa* | 2 |
|  |  | 69 | Naraoia longicaudata | 1 |
|  |  | 70 | Naraoia sponosa | 1 |
|  |  | 72 | Odaraia? eurypetala* | 1 |
|  |  | 75 | Pisinnocaris subconigera* | 1 |
|  |  | 79 | Retifacis abnormalis* | 2 |
|  |  | 81 | Saperion glumaceum | 2 |
|  |  | 82 | Sidneyia sinica | 2 |
|  |  | 91 | Waptia ovata | 1 |
|  |  | 95 | Yunaannocaris megista | 1 |
|  |  | 103 | Haikoucaris ercaiensis | 1 |
| 49 | Clypecaris pteroidea* | 4 | detritus | 1 |
| 56 | Fortiforceps foliosa | 45 | Brachiocaris? yunnanensis | 1 |
|  |  | 46 | Canadaspis laevigata* | 1 |
|  |  | 56 | Fortiforceps foliosa | 1 |
|  |  | 61 | Jinagfengia multisegmentalis | 1 |
|  |  | 65 | Kunmingella douvillei | 1 |
|  |  | 69 | Naraoia longicaudata | 1 |
|  |  | 70 | Naraoia sponosa | 1 |
|  |  | 72 | Odaraia? eurypetala* | 1 |
|  |  | 75 | Pisinnocaris subconigera* | 1 |
|  |  | 83 | Sinoburius lunaris | 1 |
|  |  | 85 | Squamacula clypeata | 1 |
|  |  | 91 | Waptia ovata | 1 |
|  |  | 95 | Yunaannocaris megista | 1 |
|  |  | 103 | Haikoucaris ercaiensis | 1 |
| 57 | Fuxianhuia protensa | 45 | Brachiocaris? yunnanensis | 1 |
|  |  | 46 | Canadaspis laevigata* | 1 |
|  |  | 47 | Chengjiangocaris longiformis | 2 |
|  |  | 56 | Fortiforceps foliosa | 1 |
|  |  | 57 | Fuxianhuia protensa | 2 |
|  |  | 61 | Jinagfengia multisegmentalis | 1 |
|  |  | 63 | Kuamaia lata | 2 |
|  |  | 67 | Leanchoilia illecebrosa* | 2 |
|  |  | 69 | Naraoia longicaudata | 1 |
|  |  | 70 | Naraoia sponosa | 1 |
|  |  | 72 | Odaraia? eurypetala* | 1 |
|  |  | 75 | Pisinnocaris subconigera* | 1 |
|  |  | 79 | Retifacis abnormalis* | 2 |
|  |  | 81 | Saperion glumaceum | 2 |
|  |  | 82 | Sidneyia sinica | 2 |
|  |  | 91 | Waptia ovata | 1 |
|  |  | 95 | Yunaannocaris megista | 1 |
|  |  | 96 | Eoredlichia intermedia | 3 |
|  |  | 97 | Kuanyangia pusulosa | 3 |
|  |  | 98 | Yunnanocephalus yunnanensis | 3 |
|  |  | 103 | Haikoucaris ercaiensis | 1 |
| 61 | Jinagfengia multisegmentalis | 45 | Brachiocaris? yunnanensis | 1 |
|  |  | 46 | Canadaspis laevigata* | 1 |
|  |  | 47 | Chengjiangocaris longiformis | 2 |
|  |  | 56 | Fortiforceps foliosa | 1 |
|  |  | 57 | Fuxianhuia protensa | 2 |
|  |  | 61 | Jinagfengia multisegmentalis | 1 |
|  |  | 63 | Kuamaia lata | 2 |
|  |  | 67 | Leanchoilia illecebrosa* | 2 |
|  |  | 69 | Naraoia longicaudata | 1 |
|  |  | 70 | Naraoia sponosa | 1 |
|  |  | 72 | Odaraia? eurypetala* | 1 |
|  |  | 75 | Pisinnocaris subconigera* | 1 |
|  |  | 79 | Retifacis abnormalis* | 2 |
|  |  | 81 | Saperion glumaceum | 2 |
|  |  | 82 | Sidneyia sinica | 2 |
|  |  | 91 | Waptia ovata | 1 |
|  |  | 95 | Yunaannocaris megista | 1 |
|  |  | 96 | Eoredlichia intermedia | 3 |
|  |  | 97 | Kuanyangia pusulosa | 3 |
|  |  | 98 | Yunnanocephalus yunnanensis | 3 |
|  |  | 103 | Haikoucaris ercaiensis | 1 |
| 63 | Kuamaia lata | 45 | Brachiocaris? yunnanensis | 1 |
|  |  | 46 | Canadaspis laevigata* | 1 |
|  |  | 47 | Chengjiangocaris longiformis | 2 |
|  |  | 56 | Fortiforceps foliosa | 1 |
|  |  | 57 | Fuxianhuia protensa | 2 |
|  |  | 61 | Jinagfengia multisegmentalis | 1 |
|  |  | 63 | Kuamaia lata | 2 |
|  |  | 67 | Leanchoilia illecebrosa* | 2 |
|  |  | 69 | Naraoia longicaudata | 1 |
|  |  | 70 | Naraoia sponosa | 1 |
|  |  | 72 | Odaraia? eurypetala* | 1 |
|  |  | 75 | Pisinnocaris subconigera* | 1 |
|  |  | 79 | Retifacis abnormalis* | 2 |
|  |  | 81 | Saperion glumaceum | 2 |
|  |  | 82 | Sidneyia sinica | 2 |
|  |  | 91 | Waptia ovata | 1 |
|  |  | 95 | Yunaannocaris megista | 1 |
|  |  | 103 | Haikoucaris ercaiensis | 1 |
| 65 | Kunmingella douvillei | 45 | Brachiocaris? yunnanensis | 1 |
|  |  | 46 | Canadaspis laevigata* | 1 |
|  |  | 47 | Chengjiangocaris longiformis | 2 |
|  |  | 56 | Fortiforceps foliosa | 1 |
|  |  | 57 | Fuxianhuia protensa | 2 |
|  |  | 61 | Jinagfengia multisegmentalis | 1 |
|  |  | 63 | Kuamaia lata | 2 |
|  |  | 67 | Leanchoilia illecebrosa* | 2 |
|  |  | 69 | Naraoia longicaudata | 1 |
|  |  | 70 | Naraoia sponosa | 1 |
|  |  | 72 | Odaraia? eurypetala* | 1 |
|  |  | 75 | Pisinnocaris subconigera* | 1 |
|  |  | 79 | Retifacis abnormalis* | 2 |
|  |  | 81 | Saperion glumaceum | 2 |
|  |  | 82 | Sidneyia sinica | 2 |
|  |  | 91 | Waptia ovata | 1 |
|  |  | 95 | Yunaannocaris megista | 1 |
|  |  | 96 | Eoredlichia intermedia | 3 |
|  |  | 97 | Kuanyangia pusulosa | 3 |
|  |  | 98 | Yunnanocephalus yunnanensis | 3 |
|  |  | 103 | Haikoucaris ercaiensis | 1 |
| 67 | Leanchoilia illecebrosa* | 45 | Brachiocaris? yunnanensis | 1 |
|  |  | 46 | Canadaspis laevigata* | 1 |
|  |  | 47 | Chengjiangocaris longiformis | 2 |
|  |  | 56 | Fortiforceps foliosa | 1 |
|  |  | 57 | Fuxianhuia protensa | 2 |
|  |  | 61 | Jinagfengia multisegmentalis | 1 |
|  |  | 63 | Kuamaia lata | 2 |
|  |  | 67 | Leanchoilia illecebrosa* | 2 |
|  |  | 69 | Naraoia longicaudata | 1 |
|  |  | 70 | Naraoia sponosa | 1 |
|  |  | 72 | Odaraia? eurypetala* | 1 |
|  |  | 75 | Pisinnocaris subconigera* | 1 |
|  |  | 79 | Retifacis abnormalis* | 2 |
|  |  | 81 | Saperion glumaceum | 2 |
|  |  | 82 | Sidneyia sinica | 2 |
|  |  | 91 | Waptia ovata | 1 |
|  |  | 95 | Yunaannocaris megista | 1 |
|  |  | 103 | Haikoucaris ercaiensis | 1 |
| 69 | Naraoia longicaudata | 45 | Brachiocaris? yunnanensis | 1 |
|  |  | 46 | Canadaspis laevigata* | 1 |
|  |  | 47 | Chengjiangocaris longiformis | 2 |
|  |  | 56 | Fortiforceps foliosa | 1 |
|  |  | 57 | Fuxianhuia protensa | 2 |
|  |  | 61 | Jinagfengia multisegmentalis | 1 |
|  |  | 63 | Kuamaia lata | 2 |
|  |  | 67 | Leanchoilia illecebrosa* | 2 |
|  |  | 69 | Naraoia longicaudata | 1 |
|  |  | 70 | Naraoia sponosa | 1 |
|  |  | 72 | Odaraia? eurypetala* | 1 |
|  |  | 75 | Pisinnocaris subconigera* | 1 |
|  |  | 79 | Retifacis abnormalis* | 2 |
|  |  | 81 | Saperion glumaceum | 2 |
|  |  | 82 | Sidneyia sinica | 2 |
|  |  | 91 | Waptia ovata | 1 |
|  |  | 95 | Yunaannocaris megista | 1 |
|  |  | 96 | Eoredlichia intermedia | 3 |
|  |  | 97 | Kuanyangia pusulosa | 3 |
|  |  | 98 | Yunnanocephalus yunnanensis | 3 |
|  |  | 103 | Haikoucaris ercaiensis | 1 |
| 70 | Naraoia sponosa | 45 | Brachiocaris? yunnanensis | 1 |
|  |  | 46 | Canadaspis laevigata* | 1 |
|  |  | 47 | Chengjiangocaris longiformis | 2 |
|  |  | 56 | Fortiforceps foliosa | 1 |
|  |  | 57 | Fuxianhuia protensa | 2 |
|  |  | 61 | Jinagfengia multisegmentalis | 1 |
|  |  | 63 | Kuamaia lata | 2 |
|  |  | 67 | Leanchoilia illecebrosa* | 2 |
|  |  | 69 | Naraoia longicaudata | 1 |
|  |  | 70 | Naraoia sponosa | 1 |
|  |  | 72 | Odaraia? eurypetala* | 1 |
|  |  | 75 | Pisinnocaris subconigera* | 1 |
|  |  | 79 | Retifacis abnormalis* | 2 |
|  |  | 81 | Saperion glumaceum | 2 |
|  |  | 82 | Sidneyia sinica | 2 |
|  |  | 91 | Waptia ovata | 1 |
|  |  | 95 | Yunaannocaris megista | 1 |
|  |  | 96 | Eoredlichia intermedia | 3 |
|  |  | 97 | Kuanyangia pusulosa | 3 |
|  |  | 98 | Yunnanocephalus yunnanensis | 3 |
|  |  | 103 | Haikoucaris ercaiensis | 1 |
| 72 | Odaraia? eurypetala* | 45 | Brachiocaris? yunnanensis | 1 |
|  |  | 46 | Canadaspis laevigata* | 1 |
|  |  | 47 | Chengjiangocaris longiformis | 2 |
|  |  | 56 | Fortiforceps foliosa | 1 |
|  |  | 57 | Fuxianhuia protensa | 2 |
|  |  | 61 | Jinagfengia multisegmentalis | 1 |
|  |  | 63 | Kuamaia lata | 2 |
|  |  | 67 | Leanchoilia illecebrosa* | 2 |
|  |  | 69 | Naraoia longicaudata | 1 |
|  |  | 70 | Naraoia sponosa | 1 |
|  |  | 72 | Odaraia? eurypetala* | 1 |
|  |  | 75 | Pisinnocaris subconigera* | 1 |
|  |  | 79 | Retifacis abnormalis* | 2 |
|  |  | 81 | Saperion glumaceum | 2 |
|  |  | 82 | Sidneyia sinica | 2 |
|  |  | 91 | Waptia ovata | 1 |
|  |  | 95 | Yunaannocaris megista | 1 |
|  |  | 96 | Eoredlichia intermedia | 3 |
|  |  | 97 | Kuanyangia pusulosa | 3 |
|  |  | 98 | Yunnanocephalus yunnanensis | 3 |
|  |  | 103 | Haikoucaris ercaiensis | 1 |
| 75 | Pisinnocaris subconigera* | 45 | Brachiocaris? yunnanensis | 1 |
|  |  | 46 | Canadaspis laevigata* | 1 |
|  |  | 47 | Chengjiangocaris longiformis | 2 |
|  |  | 56 | Fortiforceps foliosa | 1 |
|  |  | 57 | Fuxianhuia protensa | 2 |
|  |  | 61 | Jinagfengia multisegmentalis | 1 |
|  |  | 63 | Kuamaia lata | 2 |
|  |  | 67 | Leanchoilia illecebrosa* | 2 |
|  |  | 69 | Naraoia longicaudata | 1 |
|  |  | 70 | Naraoia sponosa | 1 |
|  |  | 72 | Odaraia? eurypetala* | 1 |
|  |  | 75 | Pisinnocaris subconigera* | 1 |
|  |  | 79 | Retifacis abnormalis* | 2 |
|  |  | 81 | Saperion glumaceum | 2 |
|  |  | 82 | Sidneyia sinica | 2 |
|  |  | 91 | Waptia ovata | 1 |
|  |  | 95 | Yunaannocaris megista | 1 |
|  |  | 96 | Eoredlichia intermedia | 3 |
|  |  | 97 | Kuanyangia pusulosa | 3 |
|  |  | 98 | Yunnanocephalus yunnanensis | 3 |
|  |  | 103 | Haikoucaris ercaiensis | 1 |
| 79 | Retifacis abnormalis* | 45 | Brachiocaris? yunnanensis | 1 |
|  |  | 46 | Canadaspis laevigata* | 1 |
|  |  | 47 | Chengjiangocaris longiformis | 2 |
|  |  | 56 | Fortiforceps foliosa | 1 |
|  |  | 57 | Fuxianhuia protensa | 2 |
|  |  | 61 | Jinagfengia multisegmentalis | 1 |
|  |  | 63 | Kuamaia lata | 2 |
|  |  | 67 | Leanchoilia illecebrosa* | 2 |
|  |  | 69 | Naraoia longicaudata | 1 |
|  |  | 70 | Naraoia sponosa | 1 |
|  |  | 72 | Odaraia? eurypetala* | 1 |
|  |  | 75 | Pisinnocaris subconigera* | 1 |
|  |  | 79 | Retifacis abnormalis* | 2 |
|  |  | 81 | Saperion glumaceum | 2 |
|  |  | 82 | Sidneyia sinica | 2 |
|  |  | 91 | Waptia ovata | 1 |
|  |  | 95 | Yunaannocaris megista | 1 |
|  |  | 103 | Haikoucaris ercaiensis | 1 |
| 81 | Saperion glumaceum | 45 | Brachiocaris? yunnanensis | 1 |
|  |  | 46 | Canadaspis laevigata* | 1 |
|  |  | 47 | Chengjiangocaris longiformis | 2 |
|  |  | 56 | Fortiforceps foliosa | 1 |
|  |  | 57 | Fuxianhuia protensa | 2 |
|  |  | 61 | Jinagfengia multisegmentalis | 1 |
|  |  | 63 | Kuamaia lata | 2 |
|  |  | 67 | Leanchoilia illecebrosa* | 2 |
|  |  | 69 | Naraoia longicaudata | 1 |
|  |  | 70 | Naraoia sponosa | 1 |
|  |  | 72 | Odaraia? eurypetala* | 1 |
|  |  | 75 | Pisinnocaris subconigera* | 1 |
|  |  | 79 | Retifacis abnormalis* | 2 |
|  |  | 81 | Saperion glumaceum | 2 |
|  |  | 82 | Sidneyia sinica | 2 |
|  |  | 91 | Waptia ovata | 1 |
|  |  | 95 | Yunaannocaris megista | 1 |
|  |  | 103 | Haikoucaris ercaiensis | 1 |
| 82 | Sidneyia sinica | 26 | Ambrolinevitus maximus | 2 |
|  |  | 28 | Burithes yunanensis* | 2 |
|  |  | 29 | Linevitus optimus | 2 |
|  |  | 45 | Brachiocaris? yunnanensis | 1 |
|  |  | 46 | Canadaspis laevigata* | 1 |
|  |  | 47 | Chengjiangocaris longiformis | 2 |
|  |  | 56 | Fortiforceps foliosa | 1 |
|  |  | 57 | Fuxianhuia protensa | 2 |
|  |  | 61 | Jinagfengia multisegmentalis | 1 |
|  |  | 63 | Kuamaia lata | 2 |
|  |  | 67 | Leanchoilia illecebrosa* | 2 |
|  |  | 69 | Naraoia longicaudata | 1 |
|  |  | 70 | Naraoia sponosa | 1 |
|  |  | 72 | Odaraia? eurypetala* | 1 |
|  |  | 75 | Pisinnocaris subconigera* | 1 |
|  |  | 79 | Retifacis abnormalis* | 2 |
|  |  | 81 | Saperion glumaceum | 2 |
|  |  | 82 | Sidneyia sinica | 2 |
|  |  | 91 | Waptia ovata | 1 |
|  |  | 95 | Yunaannocaris megista | 1 |
|  |  | 96 | Eoredlichia intermedia | 3 |
|  |  | 97 | Kuanyangia pusulosa | 3 |
|  |  | 98 | Yunnanocephalus yunnanensis | 3 |
|  |  | 103 | Haikoucaris ercaiensis | 1 |
| 83 | Sinoburius lunaris | 4 | detritus | 2 |
| 85 | Squamacula clypeata | 4 | detritus | 2 |
| 91 | Waptia ovata | 4 | detritus | 1 |
| 94 | Xandrella speculum | 4 | detritus | 2 |
| 95 | Yunaannocaris megista | 45 | Brachiocaris? yunnanensis | 1 |
|  |  | 46 | Canadaspis laevigata* | 1 |
|  |  | 56 | Fortiforceps foliosa | 1 |
|  |  | 61 | Jinagfengia multisegmentalis | 1 |
|  |  | 65 | Kunmingella douvillei | 1 |
|  |  | 69 | Naraoia longicaudata | 1 |
|  |  | 70 | Naraoia sponosa | 1 |
|  |  | 72 | Odaraia? eurypetala* | 1 |
|  |  | 75 | Pisinnocaris subconigera* | 1 |
|  |  | 83 | Sinoburius lunaris | 1 |
|  |  | 85 | Squamacula clypeata | 1 |
|  |  | 91 | Waptia ovata | 1 |
|  |  | 95 | Yunaannocaris megista | 1 |
|  |  | 103 | Haikoucaris ercaiensis | 1 |
| 96 | Eoredlichia intermedia | 45 | Brachiocaris? yunnanensis | 1 |
|  |  | 46 | Canadaspis laevigata* | 1 |
|  |  | 56 | Fortiforceps foliosa | 1 |
|  |  | 61 | Jinagfengia multisegmentalis | 1 |
|  |  | 65 | Kunmingella douvillei | 2 |
|  |  | 69 | Naraoia longicaudata | 1 |
|  |  | 70 | Naraoia sponosa | 1 |
|  |  | 72 | Odaraia? eurypetala* | 1 |
|  |  | 75 | Pisinnocaris subconigera* | 1 |
|  |  | 83 | Sinoburius lunaris | 1 |
|  |  | 85 | Squamacula clypeata | 1 |
|  |  | 91 | Waptia ovata | 1 |
|  |  | 95 | Yunaannocaris megista | 1 |
|  |  | 103 | Haikoucaris ercaiensis | 1 |
| 97 | Kuanyangia pusulosa | 45 | Brachiocaris? yunnanensis | 1 |
|  |  | 46 | Canadaspis laevigata* | 1 |
|  |  | 56 | Fortiforceps foliosa | 1 |
|  |  | 61 | Jinagfengia multisegmentalis | 1 |
|  |  | 65 | Kunmingella douvillei | 2 |
|  |  | 69 | Naraoia longicaudata | 1 |
|  |  | 70 | Naraoia sponosa | 1 |
|  |  | 72 | Odaraia? eurypetala* | 1 |
|  |  | 75 | Pisinnocaris subconigera* | 1 |
|  |  | 83 | Sinoburius lunaris | 1 |
|  |  | 85 | Squamacula clypeata | 1 |
|  |  | 91 | Waptia ovata | 1 |
|  |  | 95 | Yunaannocaris megista | 1 |
|  |  | 103 | Haikoucaris ercaiensis | 1 |
| 98 | Yunnanocephalus yunnanensis | 45 | Brachiocaris? yunnanensis | 1 |
|  |  | 46 | Canadaspis laevigata* | 1 |
|  |  | 56 | Fortiforceps foliosa | 1 |
|  |  | 61 | Jinagfengia multisegmentalis | 1 |
|  |  | 65 | Kunmingella douvillei | 2 |
|  |  | 69 | Naraoia longicaudata | 1 |
|  |  | 70 | Naraoia sponosa | 1 |
|  |  | 72 | Odaraia? eurypetala* | 1 |
|  |  | 75 | Pisinnocaris subconigera* | 1 |
|  |  | 83 | Sinoburius lunaris | 1 |
|  |  | 85 | Squamacula clypeata | 1 |
|  |  | 91 | Waptia ovata | 1 |
|  |  | 95 | Yunaannocaris megista | 1 |
|  |  | 103 | Haikoucaris ercaiensis | 1 |
| 99 | Amplectobelua symbrachiata | 45 | Brachiocaris? yunnanensis | 1 |
|  |  | 46 | Canadaspis laevigata* | 1 |
|  |  | 47 | Chengjiangocaris longiformis | 2 |
|  |  | 56 | Fortiforceps foliosa | 1 |
|  |  | 57 | Fuxianhuia protensa | 2 |
|  |  | 61 | Jinagfengia multisegmentalis | 1 |
|  |  | 63 | Kuamaia lata | 2 |
|  |  | 67 | Leanchoilia illecebrosa* | 2 |
|  |  | 69 | Naraoia longicaudata | 1 |
|  |  | 70 | Naraoia sponosa | 1 |
|  |  | 72 | Odaraia? eurypetala* | 1 |
|  |  | 75 | Pisinnocaris subconigera* | 1 |
|  |  | 79 | Retifacis abnormalis* | 2 |
|  |  | 81 | Saperion glumaceum | 2 |
|  |  | 82 | Sidneyia sinica | 2 |
|  |  | 91 | Waptia ovata | 1 |
|  |  | 95 | Yunaannocaris megista | 1 |
|  |  | 102 | Cucumericrus decoratus | 2 |
|  |  | 103 | Haikoucaris ercaiensis | 1 |
|  |  | 104 | Parapeytoia yunnanensis | 2 |
| 100 | Anomalocaris saron | 45 | Brachiocaris? yunnanensis | 1 |
|  |  | 46 | Canadaspis laevigata* | 1 |
|  |  | 47 | Chengjiangocaris longiformis | 2 |
|  |  | 56 | Fortiforceps foliosa | 1 |
|  |  | 57 | Fuxianhuia protensa | 2 |
|  |  | 61 | Jinagfengia multisegmentalis | 1 |
|  |  | 63 | Kuamaia lata | 2 |
|  |  | 67 | Leanchoilia illecebrosa* | 2 |
|  |  | 69 | Naraoia longicaudata | 1 |
|  |  | 70 | Naraoia sponosa | 1 |
|  |  | 72 | Odaraia? eurypetala* | 1 |
|  |  | 75 | Pisinnocaris subconigera* | 1 |
|  |  | 79 | Retifacis abnormalis* | 2 |
|  |  | 81 | Saperion glumaceum | 2 |
|  |  | 82 | Sidneyia sinica | 2 |
|  |  | 91 | Waptia ovata | 1 |
|  |  | 95 | Yunaannocaris megista | 1 |
|  |  | 102 | Cucumericrus decoratus | 2 |
|  |  | 103 | Haikoucaris ercaiensis | 1 |
|  |  | 104 | Parapeytoia yunnanensis | 2 |
| 102 | Cucumericrus decoratus | 45 | Brachiocaris? yunnanensis | 1 |
|  |  | 46 | Canadaspis laevigata* | 1 |
|  |  | 47 | Chengjiangocaris longiformis | 1 |
|  |  | 56 | Fortiforceps foliosa | 1 |
|  |  | 57 | Fuxianhuia protensa | 1 |
|  |  | 61 | Jinagfengia multisegmentalis | 1 |
|  |  | 63 | Kuamaia lata | 1 |
|  |  | 67 | Leanchoilia illecebrosa* | 1 |
|  |  | 69 | Naraoia longicaudata | 1 |
|  |  | 70 | Naraoia sponosa | 1 |
|  |  | 72 | Odaraia? eurypetala* | 1 |
|  |  | 75 | Pisinnocaris subconigera* | 1 |
|  |  | 79 | Retifacis abnormalis* | 1 |
|  |  | 81 | Saperion glumaceum | 1 |
|  |  | 82 | Sidneyia sinica | 1 |
|  |  | 91 | Waptia ovata | 1 |
|  |  | 95 | Yunaannocaris megista | 1 |
|  |  | 102 | Cucumericrus decoratus | 2 |
|  |  | 103 | Haikoucaris ercaiensis | 1 |
|  |  | 104 | Parapeytoia yunnanensis | 1 |
| 103 | Haikoucaris ercaiensis | 45 | Brachiocaris? yunnanensis | 1 |
|  |  | 46 | Canadaspis laevigata* | 1 |
|  |  | 56 | Fortiforceps foliosa | 1 |
|  |  | 61 | Jinagfengia multisegmentalis | 1 |
|  |  | 65 | Kunmingella douvillei | 1 |
|  |  | 69 | Naraoia longicaudata | 1 |
|  |  | 70 | Naraoia sponosa | 1 |
|  |  | 72 | Odaraia? eurypetala* | 1 |
|  |  | 75 | Pisinnocaris subconigera* | 1 |
|  |  | 83 | Sinoburius lunaris | 1 |
|  |  | 85 | Squamacula clypeata | 1 |
|  |  | 91 | Waptia ovata | 1 |
|  |  | 95 | Yunaannocaris megista | 1 |
|  |  | 103 | Haikoucaris ercaiensis | 1 |
| 104 | Parapeytoia yunnanensis | 45 | Brachiocaris? yunnanensis | 1 |
|  |  | 46 | Canadaspis laevigata* | 1 |
|  |  | 47 | Chengjiangocaris longiformis | 2 |
|  |  | 56 | Fortiforceps foliosa | 1 |
|  |  | 57 | Fuxianhuia protensa | 2 |
|  |  | 61 | Jinagfengia multisegmentalis | 1 |
|  |  | 63 | Kuamaia lata | 2 |
|  |  | 67 | Leanchoilia illecebrosa* | 2 |
|  |  | 69 | Naraoia longicaudata | 1 |
|  |  | 70 | Naraoia sponosa | 1 |
|  |  | 72 | Odaraia? eurypetala* | 1 |
|  |  | 75 | Pisinnocaris subconigera* | 1 |
|  |  | 79 | Retifacis abnormalis* | 2 |
|  |  | 81 | Saperion glumaceum | 2 |
|  |  | 82 | Sidneyia sinica | 2 |
|  |  | 91 | Waptia ovata | 1 |
|  |  | 95 | Yunaannocaris megista | 1 |
|  |  | 102 | Cucumericrus decoratus | 2 |
|  |  | 103 | Haikoucaris ercaiensis | 1 |
|  |  | 104 | Parapeytoia yunnanensis | 2 |
| 105 | Acosmia maotiania | 4 | detritus | 1 |
| 108 | Palaeopriapulites parvus | 4 | detritus | 1 |
| 109 | Paraselkirkia jinningensis* | 26 | Ambrolinevitus maximus | 1 |
|  |  | 27 | Ambrolinevitus ventricosus | 1 |
|  |  | 28 | Burithes yunanensis* | 1 |
|  |  | 29 | Linevitus optimus | 1 |
|  |  | 105 | Acosmia maotiania | 1 |
|  |  | 108 | Palaeopriapulites parvus | 1 |
|  |  | 109 | Paraselkirkia jinningensis* | 1 |
|  |  | 110 | Protopriapulites haikouensis* | 1 |
|  |  | 111 | Cricocosmia jinningensis | 1 |
|  |  | 112 | Maotianshania cylindrica | 1 |
| 110 | Protopriapulites haikouensis* | 4 | detritus | 1 |
| 111 | Cricocosmia jinningensis | 4 | detritus | 2 |
| 112 | Maotianshania cylindrica | 4 | detritus | 2 |
| 114 | Eldonia eumorpha* | 9 | zooplankton | 2 |
| 115 | Rotadiscus grandis | 9 | zooplankton | 2 |
| 116 | Eognathacanta ercainella | 9 | zooplankton | 2 |
| 124 | Vetulicola cuneata* | 4 | detritus | 2 |
| 126 | Dianchicystis jianshanensis | 9 | zooplankton | 1 |
| 127 | Vetulocystis catenata | 9 | zooplankton | 1 |
| 129 | Banffia confusa* | 4 | detritus | 2 |
| 131 | Coryledion tylodes* | 3 | SOM | 1 |
|  |  | 9 | zooplankton | 1 |
| 132 | Dinomischus venustus | 3 | SOM | 1 |
|  |  | 9 | zooplankton | 1 |
| 133 | Facivermis yunnanicus | 3 | SOM | 1 |
|  |  | 9 | zooplankton | 1 |
| 134 | Jiucunia petalina | 2 | bacterioplankton | 1 |
| 136 | Parvulonoda dubia | 2 | bacterioplankton | 1 |
| 138 | Yunnanozoon lividum* | 4 | detritus | 1 |
