# Supplementary material for: Compilation and Network Analyses of Cambrian Food Webs
Source: PLoS Biol. 2008 Apr 29;6(4):e102. doi: 10.1371/journal.pbio.0060102 (PMC2689700; doi:10.1371/journal.pbio.0060102)
Supplement: Table S7 — (1.07 MB DOC) [file pbio.0060102.st007.doc]

**Table S7.** Burgess Shale food-web data

Food-web data, with corresponding taxa names and numbers, for the Burgess Shale. The numbers correspond to species numbers in the master list (Table S2).

| **Con. #** | **Consumer sp** | **Res. #** | **Resource sp.** | **Certainty** |
| --- | --- | --- | --- | --- |
| 1 | phytoplankton |  |  |  |
| 2 | bacterioplankton |  |  |  |
| 3 | suspended organic matter |  |  |  |
| 4 | benthic detritus |  |  |  |
| 5 | Marpolia spissa |  |  |  |
| 6 | Morania confluens |  |  |  |
| 7 | Morania elongata |  |  |  |
| 8 | Morania fragmenta |  |  |  |
| 9 | Morania? frondosa |  |  |  |
| 10 | Morania? globosa |  |  |  |
| 11 | Morania parasitica |  |  |  |
| 12 | Morania? reticulata |  |  |  |
| 13 | Margaretia dorus |  |  |  |
| 14 | Yuknessia simplex |  |  |  |
| 15 | Bosworthia gyges |  |  |  |
| 16 | Bosworthia simulans |  |  |  |
| 17 | Dalyia nitens |  |  |  |
| 18 | Dalyia racemata |  |  |  |
| 19 | Wahpia mimica |  |  |  |
| 20 | Wahpia virgata |  |  |  |
| 21 | Waputikia ramosa |  |  |  |
| 22 | Sphaerocodium cambria |  |  |  |
| 23 | Sphaerocodium praecursor |  |  |  |
| 24 | Dictyphycus gracilis |  |  |  |
| 25 | zooplankton | 1 | Phytoplankton | 2 |
|  |  | 2 | Bacterioplankton | 2 |
|  |  | 25 | Zooplankton | 2 |
| 26 | Capsospongia undulata | 2 | Bacterioplankton | 2 |
| 27 | Choia carteri | 2 | Bacterioplankton | 2 |
| 28 | Choia ridleyi | 2 | Bacterioplankton | 2 |
| 29 | Crumillospongia biporosa | 2 | Bacterioplankton | 2 |
| 30 | Crumillospongia frondosa | 2 | Bacterioplankton | 2 |
| 31 | Falospongia falata | 2 | Bacterioplankton | 2 |
| 32 | Fieldospongia bellilineata | 2 | Bacterioplankton | 2 |
| 33 | Halichondrites elissa | 2 | Bacterioplankton | 2 |
| 34 | Hamptonia bowerbanki | 2 | Bacterioplankton | 2 |
| 35 | Hazelia conferta | 2 | Bacterioplankton | 2 |
| 36 | Hazelia crateria | 2 | Bacterioplankton | 2 |
| 37 | Hazelia delicatula | 2 | Bacterioplankton | 2 |
| 38 | Hazelia dignata | 2 | Bacterioplankton | 2 |
| 39 | Hazelia grandis | 2 | Bacterioplankton | 2 |
| 40 | Hazelia luteria | 2 | Bacterioplankton | 2 |
| 41 | Hazelia nodulifera | 2 | Bacterioplankton | 2 |
| 42 | Hazelia obscura | 2 | Bacterioplankton | 2 |
| 43 | Hazelia palmata | 2 | Bacterioplankton | 2 |
| 44 | Leptomitus lineatus | 2 | Bacterioplankton | 2 |
| 45 | Moleculopina mammilata | 2 | Bacterioplankton | 2 |
| 46 | Pirania muricata | 2 | Bacterioplankton | 2 |
| 47 | Sentinelia draco | 2 | Bacterioplankton | 2 |
| 48 | Takakkawia lineata | 2 | Bacterioplankton | 2 |
| 49 | Vauxia bellula | 2 | Bacterioplankton | 2 |
| 50 | Vauxia densa | 2 | Bacterioplankton | 2 |
| 51 | Vauxia gracilenta | 2 | Bacterioplankton | 2 |
| 52 | Vauxia venata | 2 | Bacterioplankton | 2 |
| 53 | Wapkia grandis | 2 | Bacterioplankton | 2 |
| 54 | Diagoniella hindei | 2 | Bacterioplankton | 2 |
| 55 | Protospongia hicksi | 2 | Bacterioplankton | 2 |
| 56 | Stephanospongia magnipora | 2 | Bacterioplankton | 2 |
| 57 | Canistrumella alternata | 2 | Bacterioplankton | 2 |
| 58 | Eiffelia globosa | 2 | Bacterioplankton | 2 |
| 59 | Cambrorhytium fragilis | 25 | Zooplankton | 2 |
| 60 | Cambrorhytium major | 25 | Zooplankton | 2 |
| 61 | Gelenopteron tentaculatum | 25 | Zooplankton | 2 |
| 62 | Mackenzia costalis | 25 | Zooplankton | 2 |
| 63 | Thaumaptilon walcotti | 25 | Zooplankton | 2 |
| 64 | Fasciculus vesanus | 25 | Zooplankton | 2 |
|  |  | 59 | Cambrorhytium fragilis | 2 |
|  |  | 60 | Cambrorhytium major | 2 |
|  |  | 61 | Gelenopteron tentaculatum | 2 |
|  |  | 62 | Mackenzia costalis | 2 |
|  |  | 63 | Thaumaptilon walcotti | 2 |
| 65 | Haplophrentis carinatus | 3 | suspended organic matter | 1 |
|  |  | 25 | Zooplankton | 1 |
| 66 | Scenella amii | 5 | Marpolia spissa | 1 |
|  |  | 6 | Morania confluens | 1 |
|  |  | 7 | Morania elongate | 1 |
|  |  | 8 | Morania fragmenta | 1 |
|  |  | 9 | Morania? Frondosa | 1 |
|  |  | 10 | Morania? Globosa | 1 |
|  |  | 11 | Morania parasitica | 1 |
|  |  | 12 | Morania? Reticulate | 1 |
|  |  | 13 | Margaretia dorus | 1 |
|  |  | 14 | Yuknessia simplex | 1 |
|  |  | 15 | Bosworthia gyges | 1 |
|  |  | 16 | Bosworthia simulans | 1 |
|  |  | 17 | Dalyia nitens | 1 |
|  |  | 18 | Dalyia racemata | 1 |
|  |  | 19 | Dalyia racemata | 1 |
|  |  | 20 | Wahpia virgata | 1 |
|  |  | 21 | Waputikia ramose | 1 |
|  |  | 22 | Sphaerocodium cambria | 1 |
|  |  | 23 | Sphaerocodium praecursor | 1 |
|  |  | 24 | Dictyphycus gracilis | 1 |
|  |  | 25 | Zooplankton | 2 |
| 67 | Burgessochaeta setigera | 4 | Detritus | 2 |
| 68 | Canadia spinosa | 4 | Detritus | 1 |
| 70 | Peronochaeta dubia | 4 | Detritus | 1 |
| 71 | Stephanoscolex argutus | 4 | Detritus | 1 |
| 72 | Wiwaxia corrugata | 2 | Bacterioplankton | 1 |
|  |  | 4 | Detritus | 1 |
|  |  | 7 | Morania elongate | 1 |
|  |  | 8 | Morania fragmenta | 1 |
|  |  | 9 | Morania? Frondosa | 1 |
|  |  | 10 | Morania? Globosa | 1 |
|  |  | 11 | Morania parasitica | 1 |
|  |  | 12 | Morania? Reticulate | 1 |
|  |  | 13 | Margaretia dorus | 1 |
|  |  | 14 | Yuknessia simplex | 1 |
|  |  | 15 | Bosworthia gyges | 1 |
|  |  | 16 | Bosworthia simulans | 1 |
|  |  | 17 | Dalyia nitens | 1 |
|  |  | 19 | Wahpia mimica | 1 |
|  |  | 20 | Wahpia virgata | 1 |
|  |  | 21 | Waputikia ramose | 1 |
| 73 | Acrothyra gregaria | 25 | Zooplankton | 2 |
| 74 | Lingulella waptaensis | 25 | Zooplankton | 2 |
| 75 | Micromitra burgessensis | 25 | Zooplankton | 2 |
| 76 | Paterina zenobia | 25 | Zooplankton | 2 |
| 77 | Diraphora bellicostata | 25 | Zooplankton | 2 |
| 78 | Nisusia burgessensis | 25 | Zooplankton | 2 |
| 79 | Aysheaia pedunculata | 26 | Capsospongia undulata | 1 |
|  |  | 27 | Choia carteri | 1 |
|  |  | 28 | Choia ridleyi | 1 |
|  |  | 29 | Crumillospongia biporosa | 1 |
|  |  | 30 | Crumillospongia frondosa | 1 |
|  |  | 31 | Falospongia falata | 1 |
|  |  | 32 | Fieldospongia bellilineata | 1 |
|  |  | 33 | Halichondrites elissa | 1 |
|  |  | 34 | Hamptonia bowerbanki | 1 |
|  |  | 35 | Hazelia conferta | 1 |
|  |  | 36 | Hazelia criteria | 1 |
|  |  | 37 | Hazelia delicatula | 1 |
|  |  | 38 | Hazelia dignata | 1 |
|  |  | 39 | Hazelia grandis | 1 |
|  |  | 40 | Hazelia luteria | 1 |
|  |  | 41 | Hazelia nodulifera | 1 |
|  |  | 42 | Hazelia obscura | 1 |
|  |  | 43 | Hazelia palmate | 1 |
|  |  | 44 | Leptomitus lineatus | 1 |
|  |  | 45 | Moleculopina mammilata | 1 |
|  |  | 46 | Pirania muricata | 1 |
|  |  | 47 | Sentinelia draco | 1 |
|  |  | 48 | Takakkawia lineate | 1 |
|  |  | 49 | Vauxia bellula | 1 |
|  |  | 50 | Vauxia densa | 1 |
|  |  | 51 | Vauxia gracilenta | 1 |
|  |  | 52 | Vauxia venata | 1 |
|  |  | 53 | Wapkia grandis | 1 |
|  |  | 54 | Diagoniella hindei | 1 |
|  |  | 55 | Protospongia hicksi | 1 |
|  |  | 56 | Stephanospongia magnipora | 1 |
|  |  | 57 | Canistrumella alternata | 1 |
|  |  | 58 | Eiffelia globosa | 1 |
| 80 | Hallucigenia sparsa | 26 | Capsospongia undulata | 1 |
|  |  | 27 | Choia carteri | 1 |
|  |  | 28 | Choia ridleyi | 1 |
|  |  | 29 | Crumillospongia biporosa | 1 |
|  |  | 30 | Crumillospongia frondosa | 1 |
|  |  | 31 | Falospongia falata | 1 |
|  |  | 32 | Fieldospongia bellilineata | 1 |
|  |  | 33 | Halichondrites elissa | 1 |
|  |  | 34 | Hamptonia bowerbanki | 1 |
|  |  | 35 | Hazelia conferta | 1 |
|  |  | 36 | Hazelia criteria | 1 |
|  |  | 37 | Hazelia delicatula | 1 |
|  |  | 38 | Hazelia dignata | 1 |
|  |  | 39 | Hazelia grandis | 1 |
|  |  | 40 | Hazelia luteria | 1 |
|  |  | 41 | Hazelia nodulifera | 1 |
|  |  | 42 | Hazelia obscura | 1 |
|  |  | 43 | Hazelia palmate | 1 |
|  |  | 44 | Leptomitus lineatus | 1 |
|  |  | 45 | Moleculopina mammilata | 1 |
|  |  | 46 | Pirania muricata | 1 |
|  |  | 47 | Sentinelia draco | 1 |
|  |  | 48 | Takakkawia lineate | 1 |
|  |  | 49 | Vauxia bellula | 1 |
|  |  | 50 | Vauxia densa | 1 |
|  |  | 51 | Vauxia gracilenta | 1 |
|  |  | 52 | Vauxia venata | 1 |
|  |  | 53 | Wapkia grandis | 1 |
|  |  | 54 | Diagoniella hindei | 1 |
|  |  | 55 | Protospongia hicksi | 1 |
|  |  | 56 | Stephanospongia magnipora | 1 |
|  |  | 57 | Canistrumella alternata | 1 |
|  |  | 58 | Eiffelia globosa | 1 |
| 83 | Aluta? sp. | 25 | Zooplankton | 2 |
| 85 | Burgessia bella | 4 | Detritus | 1 |
| 86 | Canadaspis ovalis | 4 | Detritus | 1 |
|  |  | 65 | Haplophrentis carinatus | 1 |
|  |  | 66 | Scenella amii | 1 |
|  |  | 67 | Burgessochaeta setigera | 1 |
|  |  | 68 | Canadia spinosa | 1 |
|  |  | 70 | Peronochaeta dubia | 1 |
|  |  | 71 | Stephanoscolex argutus | 1 |
|  |  | 83 | Aluta? sp. | 1 |
|  |  | 142 | Ancalagon minor | 1 |
|  |  | 143 | Fieldia lanceolata | 1 |
|  |  | 146 | Selkirkia columbia | 1 |
|  |  | 153 | "Ottoia" tenuis | 1 |
|  |  | 162 | Oesia disjuncta | 1 |
|  |  | 166 | Portalia mira | 1 |
| 87 | Canadaspis perfecta | 4 | detritus | 1 |
|  |  | 65 | Haplophrentis carinatus | 1 |
|  |  | 66 | Scenella amii | 1 |
|  |  | 67 | Burgessochaeta setigera | 1 |
|  |  | 68 | Canadia spinosa | 1 |
|  |  | 70 | Peronochaeta dubia | 1 |
|  |  | 71 | Stephanoscolex argutus | 1 |
|  |  | 83 | Aluta? sp. | 1 |
|  |  | 142 | Ancalagon minor | 1 |
|  |  | 143 | Fieldia lanceolata | 1 |
|  |  | 146 | Selkirkia columbia | 1 |
|  |  | 153 | "Ottoia" tenuis | 1 |
|  |  | 162 | Oesia disjuncta | 1 |
|  |  | 166 | Portalia mira | 1 |
| 89 | Emeraldella bocki | 65 | Haplophrentis carinatus | 1 |
|  |  | 66 | Scenella amii | 1 |
|  |  | 67 | Burgessochaeta setigera | 1 |
|  |  | 68 | Canadia spinosa | 1 |
|  |  | 70 | Peronochaeta dubia | 1 |
|  |  | 71 | Stephanoscolex argutus | 1 |
|  |  | 83 | Aluta? sp. | 1 |
|  |  | 142 | Ancalagon minor | 1 |
|  |  | 143 | Fieldia lanceolata | 1 |
|  |  | 146 | Selkirkia columbia | 1 |
|  |  | 153 | "Ottoia" tenuis | 1 |
|  |  | 162 | Oesia disjuncta | 1 |
|  |  | 166 | Portalia mira | 1 |
| 90 | Habelia brevicauda | 4 | detritus | 2 |
| 91 | Habelia optata | 4 | detritus | 2 |
| 92 | Helmetia expansa | 25 | zooplankton | 2 |
| 96 | Leanchoilia superlata | 65 | Haplophrentis carinatus | 2 |
|  |  | 66 | Scenella amii | 2 |
|  |  | 67 | Burgessochaeta setigera | 2 |
|  |  | 68 | Canadia spinosa | 2 |
|  |  | 70 | Peronochaeta dubia | 2 |
|  |  | 71 | Stephanoscolex argutus | 2 |
|  |  | 83 | Aluta? sp. | 2 |
|  |  | 142 | Ancalagon minor | 2 |
|  |  | 143 | Fieldia lanceolata | 2 |
|  |  | 146 | Selkirkia columbia | 2 |
|  |  | 153 | "Ottoia" tenuis | 2 |
|  |  | 162 | Oesia disjuncta | 2 |
|  |  | 166 | Portalia mira | 2 |
| 97 | Marella splendens | 1 | phytoplankton | 1 |
|  |  | 4 | detritus | 1 |
|  |  | 25 | zooplankton | 1 |
| 98 | Molaria spinifera | 4 | detritus | 2 |
| 100 | Naraoia compacta | 65 | Haplophrentis carinatus | 2 |
|  |  | 67 | Burgessochaeta setigera | 2 |
|  |  | 68 | Canadia spinosa | 2 |
|  |  | 70 | Peronochaeta dubia | 2 |
|  |  | 71 | Stephanoscolex argutus | 2 |
|  |  | 79 | Aysheaia pedunculata | 2 |
|  |  | 80 | Hallucigenia sparsa | 2 |
|  |  | 83 | Aluta? sp. | 2 |
|  |  | 98 | Molaria spinifera | 2 |
|  |  | 143 | Fieldia lanceolata | 2 |
|  |  | 144 | Louisella pedunculata | 2 |
|  |  | 145 | Ottoia prolifica | 2 |
|  |  | 146 | Selkirkia columbia | 2 |
|  |  | 162 | Oesia disjuncta | 2 |
|  |  | 166 | Portalia mira | 2 |
| 101 | Odaraia alata | 65 | Haplophrentis carinatus | 1 |
|  |  | 66 | Scenella amii | 1 |
|  |  | 67 | Burgessochaeta setigera | 1 |
|  |  | 68 | Canadia spinosa | 1 |
|  |  | 70 | Peronochaeta dubia | 1 |
|  |  | 71 | Stephanoscolex argutus | 1 |
|  |  | 83 | Aluta sp. (?) | 1 |
|  |  | 142 | Ancalagon minor | 1 |
|  |  | 143 | Fieldia lanceolata | 1 |
|  |  | 146 | Selkirkia columbia | 1 |
|  |  | 153 | "Ottoia" tenuis | 1 |
|  |  | 162 | Oesia disjuncta | 1 |
|  |  | 166 | Portalia mira | 1 |
| 102 | Perspicaris dictynna | 65 | Haplophrentis carinatus | 2 |
|  |  | 66 | Scenella amii | 2 |
|  |  | 67 | Burgessochaeta setigera | 2 |
|  |  | 68 | Canadia spinosa | 2 |
|  |  | 70 | Peronochaeta dubia | 2 |
|  |  | 71 | Stephanoscolex argutus | 2 |
|  |  | 83 | Aluta? sp. | 2 |
|  |  | 142 | Ancalagon minor | 2 |
|  |  | 143 | Fieldia lanceolata | 2 |
|  |  | 146 | Selkirkia columbia | 2 |
|  |  | 153 | "Ottoia" tenuis | 2 |
|  |  | 162 | Oesia disjuncta | 2 |
|  |  | 166 | Portalia mira | 2 |
| 103 | Perspicaris recondita | 65 | Haplophrentis carinatus | 2 |
|  |  | 66 | Scenella amii | 2 |
|  |  | 67 | Burgessochaeta setigera | 2 |
|  |  | 68 | Canadia spinosa | 2 |
|  |  | 70 | Peronochaeta dubia | 2 |
|  |  | 71 | Stephanoscolex argutus | 2 |
|  |  | 83 | Aluta? sp. | 2 |
|  |  | 142 | Ancalagon minor | 2 |
|  |  | 143 | Fieldia lanceolata | 2 |
|  |  | 146 | Selkirkia columbia | 2 |
|  |  | 153 | "Ottoia" tenuis | 2 |
|  |  | 162 | Oesia disjuncta | 2 |
|  |  | 166 | Portalia mira | 2 |
| 104 | Priscansermarius barnetti | 25 | zooplankton | 2 |
| 105 | Sanctacaris uncata | 65 | Haplophrentis carinatus | 2 |
|  |  | 66 | Scenella amii | 2 |
|  |  | 67 | Burgessochaeta setigera | 2 |
|  |  | 68 | Canadia spinosa | 2 |
|  |  | 70 | Peronochaeta dubia | 2 |
|  |  | 71 | Stephanoscolex argutus | 2 |
|  |  | 83 | Aluta? sp. | 2 |
|  |  | 85 | Burgessia bella | 2 |
|  |  | 86 | Canadaspis ovalis | 2 |
|  |  | 87 | Canadaspis perfecta | 2 |
|  |  | 89 | Emeraldella bocki | 2 |
|  |  | 90 | Habelia brevicauda | 2 |
|  |  | 91 | Habelia optata | 2 |
|  |  | 92 | Helmetia expansa | 2 |
|  |  | 96 | Leanchoilia superlata | 2 |
|  |  | 98 | Molaria spinifera | 2 |
|  |  | 100 | Naraoia compacta | 2 |
|  |  | 101 | Odaraia alata | 2 |
|  |  | 102 | Perspicaris dictynna | 2 |
|  |  | 103 | Perspicaris recondita | 2 |
|  |  | 105 | Sanctacaris uncata | 2 |
|  |  | 107 | Sidneyia inexpectans | 2 |
|  |  | 111 | Tuzoia burgessensis | 2 |
|  |  | 112 | Tuzoia canadensis | 2 |
|  |  | 113 | Tuzoia? parva | 2 |
|  |  | 114 | Tuzoia praemorsa | 2 |
|  |  | 115 | Tuzoia retifera | 2 |
|  |  | 116 | Waptia fieldensis | 2 |
|  |  | 117 | Yohoia tenuis | 2 |
|  |  | 118 | Chancia palliseri | 2 |
|  |  | 119 | Ehmaniella burgessensis | 2 |
|  |  | 120 | Ehmaniella waptaensis | 2 |
|  |  | 121 | Elrathia permulta | 2 |
|  |  | 122 | Elrathina brevifrons | 2 |
|  |  | 123 | Elrathina crodillerae | 2 |
|  |  | 126 | Olenoides serratus | 2 |
|  |  | 127 | Oryctocephalus burgessensis | 2 |
|  |  | 128 | Oryctocephalus matthewi | 2 |
|  |  | 129 | Oryctoecphalus reynoldsi | 2 |
|  |  | 130 | Pagetia bootes | 2 |
|  |  | 131 | Parkaspis decamera | 2 |
|  |  | 132 | Peronopsis montis | 2 |
|  |  | 133 | Ptychagnostus praecurrens | 2 |
|  |  | 138 | Hurdia dentata | 2 |
|  |  | 139 | Hurdia triangulata | 2 |
|  |  | 142 | Ancalagon minor | 2 |
|  |  | 143 | Fieldia lanceolata | 2 |
|  |  | 146 | Selkirkia columbia | 2 |
|  |  | 147 | Eldonia ludwigi | 2 |
|  |  | 148 | Echmatocrinus brachiatus | 2 |
|  |  | 149 | Gogia radiata | 2 |
|  |  | 150 | Walcottidiscus magister | 2 |
|  |  | 151 | Walcottidiscus typicalis | 2 |
|  |  | 153 | "Ottoia" tenuis | 2 |
|  |  | 162 | Oesia disjuncta | 2 |
|  |  | 166 | Portalia mira | 2 |
| 107 | Sidneyia inexpectans | 65 | Haplophrentis carinatus | 2 |
|  |  | 66 | Scenella amii | 2 |
|  |  | 67 | Burgessochaeta setigera | 2 |
|  |  | 68 | Canadia spinosa | 2 |
|  |  | 70 | Peronochaeta dubia | 2 |
|  |  | 71 | Stephanoscolex argutus | 2 |
|  |  | 83 | Aluta? sp. | 2 |
|  |  | 85 | Burgessia bella | 2 |
|  |  | 86 | Canadaspis ovalis | 2 |
|  |  | 87 | Canadaspis perfecta | 2 |
|  |  | 89 | Emeraldella bocki | 2 |
|  |  | 90 | Habelia brevicauda | 2 |
|  |  | 91 | Habelia optata | 2 |
|  |  | 92 | Helmetia expansa | 2 |
|  |  | 96 | Leanchoilia superlata | 2 |
|  |  | 98 | Molaria spinifera | 2 |
|  |  | 100 | Naraoia compacta | 2 |
|  |  | 101 | Odaraia alata | 2 |
|  |  | 102 | Perspicaris dictynna | 2 |
|  |  | 103 | Perspicaris recondita | 2 |
|  |  | 105 | Sanctacaris uncata | 2 |
|  |  | 107 | Sidneyia inexpectans | 2 |
|  |  | 111 | Tuzoia burgessensis | 2 |
|  |  | 112 | Tuzoia canadensis | 2 |
|  |  | 113 | Tuzoia? parva | 2 |
|  |  | 114 | Tuzoia praemorsa | 2 |
|  |  | 115 | Tuzoia retifera | 2 |
|  |  | 116 | Waptia fieldensis | 2 |
|  |  | 117 | Yohoia tenuis | 2 |
|  |  | 118 | Chancia palliseri | 2 |
|  |  | 119 | Ehmaniella burgessensis | 2 |
|  |  | 120 | Ehmaniella waptaensis | 2 |
|  |  | 121 | Elrathia permulta | 2 |
|  |  | 122 | Elrathina brevifrons | 2 |
|  |  | 123 | Elrathina crodillerae | 2 |
|  |  | 126 | Olenoides serratus | 2 |
|  |  | 127 | Oryctocephalus burgessensis | 2 |
|  |  | 128 | Oryctocephalus matthewi | 2 |
|  |  | 129 | Oryctoecphalus reynoldsi | 2 |
|  |  | 130 | Pagetia bootes | 2 |
|  |  | 131 | Parkaspis decamera | 2 |
|  |  | 132 | Peronopsis montis | 2 |
|  |  | 133 | Ptychagnostus praecurrens | 2 |
|  |  | 138 | Hurdia dentata | 2 |
|  |  | 139 | Hurdia triangulata | 2 |
|  |  | 142 | Ancalagon minor | 2 |
|  |  | 143 | Fieldia lanceolata | 2 |
|  |  | 146 | Selkirkia columbia | 2 |
|  |  | 147 | Eldonia ludwigi | 2 |
|  |  | 148 | Echmatocrinus brachiatus | 2 |
|  |  | 149 | Gogia radiata | 2 |
|  |  | 150 | Walcottidiscus magister | 2 |
|  |  | 151 | Walcottidiscus typicalis | 2 |
| 111 | Tuzoia burgessensis | 65 | Haplophrentis carinatus | 1 |
|  |  | 66 | Scenella amii | 1 |
|  |  | 67 | Burgessochaeta setigera | 1 |
|  |  | 68 | Canadia spinosa | 1 |
|  |  | 70 | Peronochaeta dubia | 1 |
|  |  | 71 | Stephanoscolex argutus | 1 |
|  |  | 83 | Aluta? sp. | 1 |
|  |  | 142 | Ancalagon minor | 1 |
|  |  | 143 | Fieldia lanceolata | 1 |
|  |  | 146 | Selkirkia columbia | 1 |
|  |  | 153 | "Ottoia" tenuis | 1 |
|  |  | 162 | Oesia disjuncta | 1 |
|  |  | 166 | Portalia mira | 1 |
| 112 | Tuzoia canadensis | 65 | Haplophrentis carinatus | 1 |
|  |  | 66 | Scenella amii | 1 |
|  |  | 67 | Burgessochaeta setigera | 1 |
|  |  | 68 | Canadia spinosa | 1 |
|  |  | 70 | Peronochaeta dubia | 1 |
|  |  | 71 | Stephanoscolex argutus | 1 |
|  |  | 83 | Aluta? sp. | 1 |
|  |  | 142 | Ancalagon minor | 1 |
|  |  | 143 | Fieldia lanceolata | 1 |
|  |  | 146 | Selkirkia columbia | 1 |
|  |  | 153 | "Ottoia" tenuis | 1 |
|  |  | 162 | Oesia disjuncta | 1 |
|  |  | 166 | Portalia mira | 1 |
| 113 | Tuzoia? parva | 65 | Haplophrentis carinatus | 1 |
|  |  | 66 | Scenella amii | 1 |
|  |  | 67 | Burgessochaeta setigera | 1 |
|  |  | 68 | Canadia spinosa | 1 |
|  |  | 70 | Peronochaeta dubia | 1 |
|  |  | 71 | Stephanoscolex argutus | 1 |
|  |  | 83 | Aluta? sp. | 1 |
|  |  | 142 | Ancalagon minor | 1 |
|  |  | 143 | Fieldia lanceolata | 1 |
|  |  | 146 | Selkirkia columbia | 1 |
|  |  | 153 | "Ottoia" tenuis | 1 |
|  |  | 162 | Oesia disjuncta | 1 |
|  |  | 166 | Portalia mira | 1 |
| 114 | Tuzoia praemorsa | 65 | Haplophrentis carinatus | 1 |
|  |  | 66 | Scenella amii | 1 |
|  |  | 67 | Burgessochaeta setigera | 1 |
|  |  | 68 | Canadia spinosa | 1 |
|  |  | 70 | Peronochaeta dubia | 1 |
|  |  | 71 | Stephanoscolex argutus | 1 |
|  |  | 83 | Aluta? sp. | 1 |
|  |  | 142 | Ancalagon minor | 1 |
|  |  | 143 | Fieldia lanceolata | 1 |
|  |  | 146 | Selkirkia columbia | 1 |
|  |  | 153 | "Ottoia" tenuis | 1 |
|  |  | 162 | Oesia disjuncta | 1 |
|  |  | 166 | Portalia mira | 1 |
| 115 | Tuzoia retifera | 65 | Haplophrentis carinatus | 1 |
|  |  | 66 | Scenella amii | 1 |
|  |  | 67 | Burgessochaeta setigera | 1 |
|  |  | 68 | Canadia spinosa | 1 |
|  |  | 70 | Peronochaeta dubia | 1 |
|  |  | 71 | Stephanoscolex argutus | 1 |
|  |  | 83 | Aluta? sp. | 1 |
|  |  | 142 | Ancalagon minor | 1 |
|  |  | 143 | Fieldia lanceolata | 1 |
|  |  | 146 | Selkirkia columbia | 1 |
|  |  | 153 | "Ottoia" tenuis | 1 |
|  |  | 162 | Oesia disjuncta | 1 |
|  |  | 166 | Portalia mira | 1 |
| 116 | Waptia fieldensis | 65 | Haplophrentis carinatus | 1 |
|  |  | 66 | Scenella amii | 1 |
|  |  | 67 | Burgessochaeta setigera | 1 |
|  |  | 68 | Canadia spinosa | 1 |
|  |  | 70 | Peronochaeta dubia | 1 |
|  |  | 71 | Stephanoscolex argutus | 1 |
|  |  | 83 | Aluta? sp. | 1 |
|  |  | 142 | Ancalagon minor | 1 |
|  |  | 143 | Fieldia lanceolata | 1 |
|  |  | 146 | Selkirkia columbia | 1 |
|  |  | 153 | "Ottoia" tenuis | 1 |
|  |  | 162 | Oesia disjuncta | 1 |
|  |  | 166 | Portalia mira | 1 |
| 117 | Yohoia tenuis | 65 | Haplophrentis carinatus | 2 |
|  |  | 66 | Scenella amii | 2 |
|  |  | 67 | Burgessochaeta setigera | 2 |
|  |  | 68 | Canadia spinosa | 2 |
|  |  | 70 | Peronochaeta dubia | 2 |
|  |  | 71 | Stephanoscolex argutus | 2 |
|  |  | 83 | Aluta? sp. | 2 |
|  |  | 142 | Ancalagon minor | 2 |
|  |  | 143 | Fieldia lanceolata | 2 |
|  |  | 146 | Selkirkia columbia | 2 |
|  |  | 153 | "Ottoia" tenuis | 2 |
|  |  | 162 | Oesia disjuncta | 2 |
|  |  | 166 | Portalia mira | 2 |
| 118 | Chancia palliseri | 4 | detritus | 1 |
| 119 | Ehmaniella burgessensis | 4 | detritus | 1 |
| 120 | Ehmaniella waptaensis | 4 | detritus | 1 |
| 121 | Elrathia permulta | 4 | detritus | 1 |
| 122 | Elrathina brevifrons | 4 | detritus | 1 |
| 123 | Elrathina crodillerae | 4 | detritus | 1 |
| 126 | Olenoides serratus | 67 | Burgessochaeta setigera | 2 |
|  |  | 68 | Canadia spinosa | 2 |
|  |  | 70 | Peronochaeta dubia | 2 |
|  |  | 71 | Stephanoscolex argutus | 2 |
|  |  | 79 | Aysheaia pedunculata | 2 |
|  |  | 80 | Hallucigenia sparsa | 2 |
|  |  | 83 | Aluta? sp. | 2 |
|  |  | 98 | Molaria spinifera | 2 |
|  |  | 143 | Fieldia lanceolata | 2 |
|  |  | 144 | Louisella pedunculata | 2 |
|  |  | 145 | Ottoia prolifica | 2 |
|  |  | 146 | Selkirkia columbia | 2 |
|  |  | 162 | Oesia disjuncta | 2 |
|  |  | 166 | Portalia mira | 2 |
| 127 | Oryctocephalus burgessensis | 67 | Burgessochaeta setigera | 1 |
|  |  | 68 | Canadia spinosa | 1 |
|  |  | 70 | Peronochaeta dubia | 1 |
|  |  | 71 | Stephanoscolex argutus | 1 |
|  |  | 79 | Aysheaia pedunculata | 1 |
|  |  | 80 | Hallucigenia sparsa | 1 |
|  |  | 83 | Aluta? sp. | 1 |
|  |  | 98 | Molaria spinifera | 1 |
|  |  | 143 | Fieldia lanceolata | 1 |
|  |  | 144 | Louisella pedunculata | 1 |
|  |  | 145 | Ottoia prolifica | 1 |
|  |  | 146 | Selkirkia columbia | 1 |
|  |  | 162 | Oesia disjuncta | 1 |
|  |  | 166 | Portalia mira | 1 |
| 128 | Oryctocephalus matthewi | 67 | Burgessochaeta setigera | 1 |
|  |  | 68 | Canadia spinosa | 1 |
|  |  | 70 | Peronochaeta dubia | 1 |
|  |  | 71 | Stephanoscolex argutus | 1 |
|  |  | 79 | Aysheaia pedunculata | 1 |
|  |  | 80 | Hallucigenia sparsa | 1 |
|  |  | 83 | Aluta? sp. | 1 |
|  |  | 98 | Molaria spinifera | 1 |
|  |  | 143 | Fieldia lanceolata | 1 |
|  |  | 144 | Louisella pedunculata | 1 |
|  |  | 145 | Ottoia prolifica | 1 |
|  |  | 146 | Selkirkia columbia | 1 |
|  |  | 162 | Oesia disjuncta | 1 |
|  |  | 166 | Portalia mira | 1 |
| 129 | Oryctoecphalus reynoldsi | 67 | Burgessochaeta setigera | 1 |
|  |  | 68 | Canadia spinosa | 1 |
|  |  | 70 | Peronochaeta dubia | 1 |
|  |  | 71 | Stephanoscolex argutus | 1 |
|  |  | 79 | Aysheaia pedunculata | 1 |
|  |  | 80 | Hallucigenia sparsa | 1 |
|  |  | 83 | Aluta? sp. | 1 |
|  |  | 98 | Molaria spinifera | 1 |
|  |  | 143 | Fieldia lanceolata | 1 |
|  |  | 144 | Louisella pedunculata | 1 |
|  |  | 145 | Ottoia prolifica | 1 |
|  |  | 146 | Selkirkia columbia | 1 |
|  |  | 162 | Oesia disjuncta | 1 |
|  |  | 166 | Portalia mira | 1 |
| 130 | Pagetia bootes | 1 | phytoplankton | 2 |
|  |  | 3 | suspended organic matter | 2 |
|  |  | 25 | zooplankton | 2 |
| 131 | Parkaspis decamera | 68 | Canadia spinosa | 1 |
|  |  | 70 | Peronochaeta dubia | 1 |
|  |  | 71 | Stephanoscolex argutus | 1 |
|  |  | 79 | Aysheaia pedunculata | 1 |
|  |  | 80 | Hallucigenia sparsa | 1 |
|  |  | 83 | Aluta? sp. | 1 |
|  |  | 98 | Molaria spinifera | 1 |
|  |  | 143 | Fieldia lanceolata | 1 |
|  |  | 144 | Louisella pedunculata | 1 |
|  |  | 145 | Ottoia prolifica | 1 |
|  |  | 146 | Selkirkia columbia | 1 |
|  |  | 162 | Oesia disjuncta | 1 |
|  |  | 166 | Portalia mira | 1 |
| 132 | Peronopsis montis | 1 | phytoplankton | 2 |
|  |  | 3 | suspended organic matter | 2 |
|  |  | 25 | zooplankton | 2 |
| 133 | Ptychagnostus praecurrens | 1 | phytoplankton | 2 |
|  |  | 3 | suspended organic matter | 2 |
|  |  | 25 | zooplankton | 2 |
| 135 | Anomalocaris canadensis | 85 | Burgessia bella | 2 |
|  |  | 86 | Canadaspis ovalis | 2 |
|  |  | 87 | Canadaspis perfecta | 2 |
|  |  | 89 | Emeraldella bocki | 2 |
|  |  | 90 | Habelia brevicauda | 2 |
|  |  | 91 | Habelia optata | 2 |
|  |  | 92 | Helmetia expansa | 2 |
|  |  | 96 | Leanchoilia superlata | 2 |
|  |  | 98 | Molaria spinifera | 2 |
|  |  | 100 | Naraoia compacta | 2 |
|  |  | 101 | Odaraia alata | 2 |
|  |  | 102 | Perspicaris dictynna | 2 |
|  |  | 103 | Perspicaris recondita | 2 |
|  |  | 105 | Sanctacaris uncata | 2 |
|  |  | 107 | Sidneyia inexpectans | 2 |
|  |  | 111 | Tuzoia burgessensis | 2 |
|  |  | 112 | Tuzoia canadensis | 2 |
|  |  | 113 | Tuzoia? parva | 2 |
|  |  | 114 | Tuzoia praemorsa | 2 |
|  |  | 115 | Tuzoia retifera | 2 |
|  |  | 116 | Waptia fieldensis | 2 |
|  |  | 117 | Yohoia tenuis | 2 |
|  |  | 118 | Chancia palliseri | 2 |
|  |  | 119 | Ehmaniella burgessensis | 2 |
|  |  | 120 | Ehmaniella waptaensis | 2 |
|  |  | 121 | Elrathia permulta | 2 |
|  |  | 122 | Elrathina brevifrons | 2 |
|  |  | 123 | Elrathina crodillerae | 2 |
|  |  | 126 | Olenoides serratus | 2 |
|  |  | 127 | Oryctocephalus burgessensis | 2 |
|  |  | 128 | Oryctocephalus matthewi | 2 |
|  |  | 129 | Oryctoecphalus reynoldsi | 2 |
|  |  | 130 | Pagetia bootes | 2 |
|  |  | 131 | Parkaspis decamera | 2 |
|  |  | 132 | Peronopsis montis | 2 |
|  |  | 133 | Ptychagnostus praecurrens | 2 |
|  |  | 138 | Hurdia dentata | 2 |
|  |  | 139 | Hurdia triangulata | 2 |
|  |  | 140 | Hurdia vicroria | 2 |
| 136 | Laggania nathorsti | 85 | Burgessia bella | 2 |
|  |  | 86 | Canadaspis ovalis | 2 |
|  |  | 87 | Canadaspis perfecta | 2 |
|  |  | 89 | Emeraldella bocki | 2 |
|  |  | 90 | Habelia brevicauda | 2 |
|  |  | 91 | Habelia optata | 2 |
|  |  | 92 | Helmetia expansa | 2 |
|  |  | 96 | Leanchoilia superlata | 2 |
|  |  | 98 | Molaria spinifera | 2 |
|  |  | 100 | Naraoia compacta | 2 |
|  |  | 101 | Odaraia alata | 2 |
|  |  | 102 | Perspicaris dictynna | 2 |
|  |  | 103 | Perspicaris recondita | 2 |
|  |  | 105 | Sanctacaris uncata | 2 |
|  |  | 107 | Sidneyia inexpectans | 2 |
|  |  | 111 | Tuzoia burgessensis | 2 |
|  |  | 112 | Tuzoia canadensis | 2 |
|  |  | 113 | Tuzoia? parva | 2 |
|  |  | 114 | Tuzoia praemorsa | 2 |
|  |  | 115 | Tuzoia retifera | 2 |
|  |  | 116 | Waptia fieldensis | 2 |
|  |  | 117 | Yohoia tenuis | 2 |
|  |  | 118 | Chancia palliseri | 2 |
|  |  | 119 | Ehmaniella burgessensis | 2 |
|  |  | 120 | Ehmaniella waptaensis | 2 |
|  |  | 121 | Elrathia permulta | 2 |
|  |  | 122 | Elrathina brevifrons | 2 |
|  |  | 123 | Elrathina crodillerae | 2 |
|  |  | 126 | Olenoides serratus | 2 |
|  |  | 127 | Oryctocephalus burgessensis | 2 |
|  |  | 128 | Oryctocephalus matthewi | 2 |
|  |  | 129 | Oryctoecphalus reynoldsi | 2 |
|  |  | 130 | Pagetia bootes | 2 |
|  |  | 131 | Parkaspis decamera | 2 |
|  |  | 132 | Peronopsis montis | 2 |
|  |  | 133 | Ptychagnostus praecurrens | 2 |
|  |  | 138 | Hurdia dentata | 2 |
|  |  | 139 | Hurdia triangulata | 2 |
|  |  | 140 | Hurdia vicroria | 2 |
| 138 | Hurdia dentata | 25 | zooplankton | 1 |
|  |  | 85 | Burgessia bella | 2 |
|  |  | 86 | Canadaspis ovalis | 2 |
|  |  | 87 | Canadaspis perfecta | 2 |
|  |  | 97 | Marella splendens | 2 |
|  |  | 100 | Naraoia compacta | 2 |
|  |  | 116 | Waptia fieldensis | 2 |
|  |  | 117 | Yohoia tenuis | 2 |
|  |  | 118 | Chancia palliseri | 2 |
|  |  | 127 | Oryctocephalus burgessensis | 2 |
|  |  | 128 | Oryctocephalus matthewi | 2 |
|  |  | 129 | Oryctoecphalus reynoldsi | 2 |
| 139 | Hurdia triangulata | 25 | zooplankton | 1 |
|  |  | 85 | Burgessia bella | 2 |
|  |  | 86 | Canadaspis ovalis | 2 |
|  |  | 87 | Canadaspis perfecta | 2 |
|  |  | 97 | Marella splendens | 2 |
|  |  | 100 | Naraoia compacta | 2 |
|  |  | 116 | Waptia fieldensis | 2 |
|  |  | 117 | Yohoia tenuis | 2 |
|  |  | 118 | Chancia palliseri | 2 |
|  |  | 127 | Oryctocephalus burgessensis | 2 |
|  |  | 128 | Oryctocephalus matthewi | 2 |
|  |  | 129 | Oryctoecphalus reynoldsi | 2 |
| 140 | Hurdia vicroria | 25 | zooplankton | 1 |
|  |  | 85 | Burgessia bella | 2 |
|  |  | 86 | Canadaspis ovalis | 2 |
|  |  | 87 | Canadaspis perfecta | 2 |
|  |  | 97 | Marella splendens | 2 |
|  |  | 100 | Naraoia compacta | 2 |
|  |  | 116 | Waptia fieldensis | 2 |
|  |  | 117 | Yohoia tenuis | 2 |
|  |  | 118 | Chancia palliseri | 2 |
|  |  | 127 | Oryctocephalus burgessensis | 2 |
|  |  | 128 | Oryctocephalus matthewi | 2 |
|  |  | 129 | Oryctoecphalus reynoldsi | 2 |
| 141 | Opabinia regalis | 65 | Haplophrentis carinatus | 2 |
|  |  | 67 | Burgessochaeta setigera | 2 |
|  |  | 68 | Canadia spinosa | 2 |
|  |  | 70 | Peronochaeta dubia | 2 |
|  |  | 71 | Stephanoscolex argutus | 2 |
|  |  | 83 | Aluta? Sp. | 2 |
|  |  | 142 | Ancalagon minor | 2 |
|  |  | 143 | Fieldia lanceolata | 2 |
|  |  | 144 | Louisella pedunculata | 2 |
|  |  | 145 | Ottoia prolifica | 2 |
|  |  | 146 | Selkirkia columbia | 2 |
|  |  | 153 | "Ottoia" tenuis | 2 |
|  |  | 162 | Oesia disjuncta | 2 |
|  |  | 166 | Portalia mira | 2 |
| 142 | Ancalagon minor | 65 | Haplophrentis carinatus | 2 |
|  |  | 66 | Scenella amii | 2 |
|  |  | 67 | Burgessochaeta setigera | 2 |
|  |  | 68 | Canadia spinosa | 2 |
|  |  | 70 | Peronochaeta dubia | 2 |
|  |  | 71 | Stephanoscolex argutus | 2 |
|  |  | 83 | Aluta? sp. | 1 |
|  |  | 142 | Ancalagon minor | 2 |
|  |  | 143 | Fieldia lanceolata | 2 |
|  |  | 146 | Selkirkia columbia | 2 |
|  |  | 153 | "Ottoia" tenuis | 1 |
|  |  | 162 | Oesia disjuncta | 1 |
|  |  | 166 | Portalia mira | 1 |
| 143 | Fieldia lanceolata | 3 | suspended organic matter | 1 |
| 144 | Louisella pedunculata | 65 | Haplophrentis carinatus | 1 |
|  |  | 66 | Scenella amii | 1 |
|  |  | 67 | Burgessochaeta setigera | 1 |
|  |  | 68 | Canadia spinosa | 1 |
|  |  | 70 | Peronochaeta dubia | 1 |
|  |  | 71 | Stephanoscolex argutus | 1 |
|  |  | 83 | Aluta? sp. | 1 |
|  |  | 142 | Ancalagon minor | 1 |
|  |  | 143 | Fieldia lanceolata | 1 |
|  |  | 146 | Selkirkia columbia | 1 |
|  |  | 153 | "Ottoia" tenuis | 1 |
|  |  | 162 | Oesia disjuncta | 1 |
|  |  | 166 | Portalia mira | 1 |
| 145 | Ottoia prolifica | 65 | Haplophrentis carinatus | 3 |
|  |  | 66 | Scenella amii | 1 |
|  |  | 67 | Burgessochaeta setigera | 1 |
|  |  | 68 | Canadia spinosa | 1 |
|  |  | 70 | Peronochaeta dubia | 1 |
|  |  | 71 | Stephanoscolex argutus | 1 |
|  |  | 83 | Aluta? sp. | 1 |
|  |  | 142 | Ancalagon minor | 1 |
|  |  | 143 | Fieldia lanceolata | 1 |
|  |  | 146 | Selkirkia columbia | 1 |
|  |  | 153 | "Ottoia" tenuis | 1 |
|  |  | 162 | Oesia disjuncta | 1 |
|  |  | 166 | Portalia mira | 1 |
| 146 | Selkirkia columbia | 65 | Haplophrentis carinatus | 1 |
|  |  | 66 | Scenella amii | 1 |
|  |  | 67 | Burgessochaeta setigera | 1 |
|  |  | 68 | Canadia spinosa | 1 |
|  |  | 70 | Peronochaeta dubia | 1 |
|  |  | 71 | Stephanoscolex argutus | 1 |
|  |  | 83 | Aluta? sp. | 1 |
|  |  | 142 | Ancalagon minor | 1 |
|  |  | 143 | Fieldia lanceolata | 1 |
|  |  | 146 | Selkirkia columbia | 1 |
|  |  | 153 | "Ottoia" tenuis | 1 |
|  |  | 162 | Oesia disjuncta | 1 |
|  |  | 166 | Portalia mira | 1 |
| 147 | Eldonia ludwigi | 25 | zooplankton | 1 |
| 148 | Echmatocrinus brachiatus | 25 | zooplankton | 1 |
| 149 | Gogia radiata | 25 | zooplankton | 1 |
| 150 | Walcottidiscus magister | 25 | zooplankton | 1 |
| 151 | Walcottidiscus typicalis | 25 | zooplankton | 1 |
| 152 | Chaunograptus scandens | 25 | zooplankton | 1 |
| 153 | "Ottoia" tenuis | 25 | zooplankton | 1 |
| 155 | Pikaia gracilens | 25 | zooplankton | 2 |
| 158 | Chancelloria eros | 1 | phytoplankton | 2 |
|  |  | 25 | zooplankton | 2 |
| 159 | Dinomischus isolatus | 3 | suspended organic matter | 1 |
|  |  | 25 | zooplankton | 1 |
| 160 | Nectocaris pteryx | 65 | Haplophrentis carinatus | 2 |
|  |  | 67 | Burgessochaeta setigera | 2 |
|  |  | 68 | Canadia spinosa | 2 |
|  |  | 70 | Peronochaeta dubia | 2 |
|  |  | 71 | Stephanoscolex argutus | 2 |
|  |  | 83 | Aluta? sp. | 2 |
|  |  | 142 | Ancalagon minor | 2 |
|  |  | 143 | Fieldia lanceolata | 2 |
|  |  | 144 | Louisella pedunculata | 2 |
|  |  | 153 | "Ottoia" tenuis | 2 |
|  |  | 162 | Oesia disjuncta | 2 |
|  |  | 166 | Portalia mira | 2 |
| 162 | Oesia disjuncta | 25 | zooplankton | 2 |
| 166 | Portalia mira | 25 | zooplankton | 1 |
