# Supplementary material for: Compilation and Network Analyses of Cambrian Food Webs
Source: PLoS Biol. 2008 Apr 29;6(4):e102. doi: 10.1371/journal.pbio.0060102 (PMC2689700; doi:10.1371/journal.pbio.0060102)
Supplement: Table S8 — (124 KB DOC) [file pbio.0060102.st008.doc]

**Table S8.** Chengjiang Shale trophic species groupings

Species that share the same set of consumers and resources are grouped into “trophic species.” 85 Chengjiang Shale food-web taxa (Table S6) fall into 33 “trophic species,” as indicated by .tro # and delineated by horizontal lines. The 5 original aggregated groups (phytoplankton, bacterioplankton, suspended organic matter, benthic detritus, and zooplankton) remain as separate trophic species. 9 of the original species remain as separate trophic species. The remaining 71 species group into 19 additional trophic species. The number for each taxon within each trophic species is also given (.web #—see Tables S1, S6), as well as the corresponding sequential renumbering of the food-web species (seq. #), eliminating numbering gaps due to species not included in the final food web (Table S4).

| **.tro #** | **Taxon** | **.web #** | **seq. #** |
| --- | --- | --- | --- |
| 1 | phytoplankton | 1 | 1 |
| 2 | bacterioplankton | 2 | 2 |
| 3 | suspended organic matter | 3 | 3 |
| 4 | benthic detritus | 4 | 4 |
| 5 | zooplankton | 9 | 5 |
| 6 | Allantospongia mica | 10 | 6 |
|  | Choia xiaolantianensis | 11 | 7 |
|  | Choiaella radiata | 12 | 8 |
|  | Leptomitella confusa | 13 | 9 |
|  | Leptomitella conica | 14 | 10 |
|  | Leptomitus teretiusculus | 15 | 11 |
|  | Paraleptomitella dictyodroma | 16 | 12 |
|  | Paraleptomitella globosa | 17 | 13 |
|  | Quadrolaminella crassa | 18 | 14 |
|  | Sinoflabrum antiquum | 19 | 15 |
|  | Saetaspongia densa | 20 | 16 |
|  | Triticispongia diagonata | 21 | 17 |
| 7 | Priscapennamaria angusta | 22 | 18 |
|  | Xianguangia sinica | 23 | 19 |
| 8 | Maotianoascus octonarius | 24 | 20 |
|  | Sinoascus papillatus | 25 | 21 |
| 9 | Ambrolinevitus maximus | 26 | 22 |
|  | Burithes yunanensis* | 28 | 24 |
|  | Linevitus optimus | 29 | 25 |
| 10 | Ambrolinevitus ventricosus | 27 | 23 |
| 11 | Lotuba chengjiangensis | 31 | 26 |
|  | Heliomedusa orienta | 32 | 27 |
|  | Diandongia pista | 33 | 28 |
|  | Lingulella chengjiangensis | 34 | 29 |
|  | Lingulellotreta malongensis | 35 | 30 |
|  | Longtancunella chengiangensis | 36 | 31 |
| 12 | Hallucigenia fortis | 38 | 32 |
|  | Luolishania longicruris | 39 | 33 |
|  | Onychodictyon ferox | 41 | 35 |
| 13 | Microdictyon sinicum | 40 | 34 |
| 14 | Paucipodia inermis | 42 | 36 |
| 15 | Brachiocaris? yunnanensis | 45 | 37 |
| 16 | Canadaspis laevigata* | 46 | 38 |
|  | Waptia ovata | 91 | 56 |
| 17 | Chengjiangocaris longiformis | 47 | 39 |
|  | Kuamaia lata | 63 | 44 |
|  | Leanchoilia illecebrosa* | 67 | 46 |
|  | Retifacis abnormalis* | 79 | 51 |
|  | Saperion glumaceum | 81 | 52 |
| 18 | Clypecaris pteroidea* | 49 | 40 |
|  | Xandrella speculum | 94 | 57 |
|  | Vetulicola cuneata* | 124 | 76 |
|  | Banffia confusa* | 129 | 79 |
| 19 | Fortiforceps foliosa | 56 | 41 |
|  | Yunaannocaris megista | 95 | 58 |
|  | Haikoucaris ercaiensis | 103 | 65 |
| 20 | Fuxianhuia protensa | 57 | 42 |
| 21 | Jinagfengia multisegmentalis | 61 | 43 |
|  | Naraoia longicaudata | 69 | 47 |
|  | Naraoia sponosa | 70 | 48 |
|  | Odaraia? eurypetala* | 72 | 49 |
|  | Pisinnocaris subconigera* | 75 | 50 |
|  | Yunnanozoon lividum* | 138 | 85 |
| 22 | Kunmingella douvillei | 65 | 45 |
| 23 | Sidneyia sinica | 82 | 53 |
| 24 | Sinoburius lunaris | 83 | 54 |
|  | Squamacula clypeata | 85 | 55 |
| 25 | Eoreclichia intermedia | 96 | 59 |
|  | Kuanyangia pusulosa | 97 | 60 |
|  | Yunnanocephalus yunnanensis | 98 | 61 |
| 26 | Amplectobelua symbrachiata | 99 | 62 |
|  | Anomalocaris saron | 100 | 63 |
| 27 | Cucumericrus decoratus | 102 | 64 |
|  | Parapeytoia yunnanensis | 104 | 66 |
| 28 | Acosmia maotiania | 105 | 67 |
|  | Palaeopriapulites parvus | 108 | 68 |
|  | Protopriapulites haikouensis* | 110 | 70 |
|  | Cricocosmia jinningensis | 111 | 71 |
|  | Maotianshania cylindrica | 112 | 72 |
| 29 | Paraselkirkia jinningensis* | 109 | 69 |
| 30 | Eldonia eumorpha* | 114 | 73 |
| 31 | Rotadiscus grandis | 115 | 74 |
|  | Eognathacanta ercainella | 116 | 75 |
|  | Dianchicystis jianshanensis | 126 | 77 |
|  | Vetulocystis catenata | 127 | 78 |
| 32 | Coryledion tylodes* | 131 | 80 |
|  | Dinomischus venustus | 132 | 81 |
|  | Facivermis yunnanicus | 133 | 82 |
| 33 | Jiucunia petalina | 134 | 83 |
|  | Parvulonoda dubia | 136 | 84 |
