# Supplementary material for: Compilation and Network Analyses of Cambrian Food Webs
Source: PLoS Biol. 2008 Apr 29;6(4):e102. doi: 10.1371/journal.pbio.0060102 (PMC2689700; doi:10.1371/journal.pbio.0060102)
Supplement: Table S9 — (194 KB DOC) [file pbio.0060102.st009.doc]

**Table S9.** Burgess Shale trophic species groupings

Species that share the same set of consumers and resources are grouped into “trophic species.” 142 Burgess Shale food-web taxa fall into 48 “trophic species,” as indicated by .tro # and delineated by horizontal lines. The 5 original aggregated groups (phytoplankton, bacterioplankton, suspended organic matter, benthic detritus, and zooplankton) remain as separate trophic species. 25 of the original species remain as separate trophic species. The remaining 112 species group into 18 additional trophic species. The number for each taxon within each trophic species is also given (.web #—see Tables S2, S7), as well as the corresponding sequential renumbering of the food-web species (seq. #), eliminating numbering gaps due to species not included in the final food web (Table S5).

| **.tro #** | **Taxon** | **.web #** | **seq. #** |
| --- | --- | --- | --- |
| 1 | phytoplankton | 1 | 1 |
| 2 | bacterioplankton | 2 | 2 |
| 3 | suspended organic matter | 3 | 3 |
| 4 | benthic detritus | 4 | 4 |
| 5 | Marpolia spissa | 5 | 5 |
|  | Morania confluens | 6 | 6 |
|  | Dalyia racemata | 18 | 18 |
|  | Sphaerocodium cambria | 22 | 22 |
|  | Sphaerocodium praecursor | 23 | 23 |
|  | Dictyphycus gracilis | 24 | 24 |
| 6 | Morania elongata | 7 | 7 |
|  | Morania fragmenta | 8 | 8 |
|  | Morania? frondosa | 9 | 9 |
|  | Morania? globosa | 10 | 10 |
|  | Morania parasitica | 11 | 11 |
|  | Morania? reticulata | 12 | 12 |
|  | Margaretia dorus | 13 | 13 |
|  | Yuknessia simplex | 14 | 14 |
|  | Bosworthia gyges | 15 | 15 |
|  | Bosworthia simulans | 16 | 16 |
|  | Dalyia nitens | 17 | 17 |
|  | Wahpia mimica | 19 | 19 |
|  | Wahpia virgata | 20 | 20 |
|  | Waputikia ramosa | 21 | 21 |
| 7 | zooplankton | 25 | 25 |
| 8 | Capsospongia undulata | 26 | 26 |
|  | Choia carteri | 27 | 27 |
|  | Choia ridleyi | 28 | 28 |
|  | Crumillospongia biporosa | 29 | 29 |
|  | Crumillospongia frondosa | 30 | 30 |
|  | Falospongia falata | 31 | 31 |
|  | Fieldospongia bellilineata | 32 | 32 |
|  | Halichondrites elissa | 33 | 33 |
|  | Hamptonia bowerbanki | 34 | 34 |
|  | Hazelia conferta | 35 | 35 |
|  | Hazelia crateria | 36 | 36 |
|  | Hazelia delicatula | 37 | 37 |
|  | Hazelia dignata | 38 | 38 |
|  | Hazelia grandis | 39 | 39 |
|  | Hazelia luteria | 40 | 40 |
|  | Hazelia nodulifera | 41 | 41 |
|  | Hazelia obscura | 42 | 42 |
|  | Hazelia palmata | 43 | 43 |
|  | Leptomitus lineatus | 44 | 44 |
|  | Moleculopina mammilata | 45 | 45 |
|  | Pirania muricata | 46 | 46 |
|  | Sentinelia draco | 47 | 47 |
|  | Takakkawia lineata | 48 | 48 |
|  | Vauxia bellula | 49 | 49 |
|  | Vauxia densa | 50 | 50 |
|  | Vauxia gracilenta | 51 | 51 |
|  | Vauxia venata | 52 | 52 |
|  | Wapkia grandis | 53 | 53 |
|  | Diagoniella hindei | 54 | 54 |
|  | Protospongia hicksi | 55 | 55 |
|  | Stephanospongia magnipora | 56 | 56 |
|  | Canistrumella alternata | 57 | 57 |
|  | Eiffelia globosa | 58 | 58 |
| 9 | Cambrorhytium fragilis | 59 | 59 |
|  | Cambrorhytium major | 60 | 60 |
|  | Gelenopteron tentaculatum | 61 | 61 |
|  | Mackenzia costalis | 62 | 62 |
|  | Thaumaptilon walcotti | 63 | 63 |
| 10 | Fasciculus vesanus | 64 | 64 |
| 11 | Haplophrentis carinatus | 65 | 65 |
| 12 | Scenella amii | 66 | 66 |
| 13 | Burgessochaeta setigera | 67 | 67 |
| 14 | Canadia spinosa | 68 | 68 |
|  | Peronochaeta dubia | 70 | 69 |
|  | Stephanoscolex argutus | 71 | 70 |
| 15 | Wiwaxia corrugata | 72 | 71 |
| 16 | Acrothyra gregaria | 73 | 72 |
|  | Lingulella waptaensis | 74 | 73 |
|  | Micromitra burgessensis | 75 | 74 |
|  | Paterina zenobia | 76 | 75 |
|  | Diraphora bellicostata | 77 | 76 |
|  | Nisusia burgessensis | 78 | 77 |
|  | Priscansermarius barnetti | 104 | 95 |
|  | Chaunograptus scandens | 152 | 135 |
|  | Pikaia gracilens | 155 | 137 |
| 17 | Aysheaia pedunculata | 79 | 78 |
|  | Hallucigenia sparsa | 80 | 79 |
| 18 | Aluta sp. (?) | 83 | 80 |
| 19 | Burgessia bella | 85 | 81 |
|  | Chancia palliseri | 118 | 105 |
| 20 | Canadaspis ovalis | 86 | 82 |
|  | Canadaspis perfecta | 87 | 83 |
| 21 | Emeraldella bocki | 89 | 84 |
|  | Leanchoilia superlata | 96 | 88 |
|  | Odaraia alata | 101 | 92 |
|  | Perspicaris dictynna | 102 | 93 |
|  | Perspicaris recondita | 103 | 94 |
|  | Tuzoia burgessensis | 111 | 98 |
|  | Tuzoia canadensis | 112 | 99 |
|  | Tuzoia? parva | 113 | 100 |
|  | Tuzoia praemorsa | 114 | 101 |
|  | Tuzoia retifera | 115 | 102 |
| 22 | Habelia brevicauda | 90 | 85 |
|  | Habelia optata | 91 | 86 |
|  | Ehmaniella burgessensis | 119 | 106 |
|  | Ehmaniella waptaensis | 120 | 107 |
|  | Elrathia permulta | 121 | 108 |
|  | Elrathina brevifrons | 122 | 109 |
|  | Elrathina crodillerae | 123 | 110 |
| 23 | Helmetia expansa | 92 | 87 |
| 24 | Marella splendens | 97 | 89 |
| 25 | Molaria spinifera | 98 | 90 |
| 26 | Naraoia compacta | 100 | 91 |
| 27 | Sanctacaris uncata | 105 | 96 |
| 28 | Sidneyia inexpectans | 107 | 97 |
| 29 | Waptia fieldensis | 116 | 103 |
|  | Yohoia tenuis | 117 | 104 |
| 30 | Olenoides serratus | 126 | 111 |
| 31 | Oryctocephalus burgessensis | 127 | 112 |
|  | Oryctocephalus matthewi | 128 | 113 |
|  | Oryctoecphalus reynoldsi | 129 | 114 |
| 32 | Pagetia bootes | 130 | 115 |
|  | Peronopsis montis | 132 | 117 |
|  | Ptychagnostus praecurrens | 133 | 118 |
| 33 | Parkaspis decamera | 131 | 116 |
| 34 | Anomalocaris canadensis | 135 | 119 |
|  | Laggania nathorsti | 136 | 120 |
| 35 | Hurdia dentata | 138 | 121 |
|  | Hurdia triangulata | 139 | 122 |
| 36 | Hurdia vicroria | 140 | 123 |
| 37 | Opabinia regalis | 141 | 124 |
| 38 | Ancalagon minor | 142 | 125 |
| 39 | Fieldia lanceolata | 143 | 126 |
| 40 | Louisella pedunculata | 144 | 127 |
| 41 | Ottoia prolifica | 145 | 128 |
| 42 | Selkirkia columbia | 146 | 129 |
| 43 | Eldonia ludwigi | 147 | 130 |
|  | Echmatocrinus brachiatus | 148 | 131 |
|  | Gogia radiata | 149 | 132 |
|  | Walcottidiscus magister | 150 | 133 |
|  | Walcottidiscus typicalis | 151 | 134 |
| 44 | "Ottoia" tenuis | 153 | 136 |
| 45 | Chancelloria eros | 158 | 138 |
| 46 | Dinomischus isolatus | 159 | 139 |
| 47 | Nectocaris pteryx | 160 | 140 |
| 48 | Oesia disjuncta | 162 | 141 |
|  | Portalia mira | 166 | 142 |
