# Supplementary material for: Compilation and Network Analyses of Cambrian Food Webs
Source: PLoS Biol. 2008 Apr 29;6(4):e102. doi: 10.1371/journal.pbio.0060102 (PMC2689700; doi:10.1371/journal.pbio.0060102)
Supplement: Table S10 — (201 KB DOC) [file pbio.0060102.st010.doc]

**Table S10.** Trophic species food-web data for the Chengjiang and Burgess Shales

The numbers that identify consumers (“cons”) and resources (“res”) correspond to the sequential numbers (seq. #) from Table S8 (Chengjiang Shale) and Table S9 (Burgess Shale), not to the original numbering of taxa (Tables S1, S2). “cert” refers to certainty of each link, which is an average of the certainty of the original links that overlap once taxa are aggregated into trophic species. *S* = trophic species, *L* = links.

**Table S10a.** Chengijaing Shale (*S* = 33, *L* = 99)

| **cons** | **res** | **cert** | **cons** | **res** | **cert** | **cons** | **res** | **cert** | **cons** | **res** | **cert** |
| --- | --- | --- | --- | --- | --- | --- | --- | --- | --- | --- | --- |
| **5** | **1** | **3** | **17** | **23** | **2** | **22** | **16** | **1** | **26** | **17** | **2** |
| **5** | **2** | **3** | **18** | **4** | **1.44** | **22** | **17** | **2** | **26** | **19** | **1** |
| **6** | **2** | **2** | **19** | **15** | **1** | **22** | **19** | **1** | **26** | **20** | **2** |
| **7** | **5** | **1** | **19** | **16** | **1** | **22** | **20** | **2** | **26** | **21** | **1** |
| **8** | **5** | **2** | **19** | **19** | **1** | **22** | **21** | **1** | **26** | **23** | **2** |
| **8** | **7** | **1** | **19** | **21** | **1** | **22** | **23** | **2** | **26** | **27** | **2** |
| **8** | **8** | **1** | **19** | **22** | **1** | **22** | **25** | **3** | **27** | **15** | **1** |
| **9** | **3** | **1** | **19** | **24** | **1** | **23** | **9** | **2** | **27** | **16** | **1** |
| **9** | **5** | **1** | **20** | **15** | **1** | **23** | **15** | **1** | **27** | **17** | **1.5** |
| **10** | **3** | **1** | **20** | **16** | **1** | **23** | **16** | **1** | **27** | **19** | **1** |
| **10** | **5** | **1** | **20** | **17** | **2** | **23** | **17** | **2** | **27** | **20** | **1.5** |
| **11** | **2** | **2** | **20** | **19** | **1** | **23** | **19** | **1** | **27** | **21** | **1** |
| **11** | **3** | **1** | **20** | **20** | **2** | **23** | **20** | **2** | **27** | **23** | **1.5** |
| **12** | **6** | **1** | **20** | **21** | **1** | **23** | **21** | **1** | **27** | **27** | **1.75** |
| **13** | **6** | **1** | **20** | **23** | **2** | **23** | **23** | **2** | **28** | **4** | **1.75** |
| **13** | **30** | **1** | **20** | **25** | **3** | **23** | **25** | **3** | **29** | **9** | **1** |
| **14** | **30** | **2** | **21** | **15** | **1** | **24** | **4** | **2** | **29** | **10** | **1** |
| **15** | **5** | **2** | **21** | **16** | **1** | **25** | **15** | **1** | **29** | **28** | **1** |
| **16** | **4** | **1.5** | **21** | **17** | **2** | **25** | **16** | **1** | **29** | **29** | **1** |
| **17** | **15** | **1** | **21** | **19** | **1** | **25** | **19** | **1** | **30** | **5** | **2** |
| **17** | **16** | **1** | **21** | **20** | **2** | **25** | **21** | **1** | **31** | **5** | **1.25** |
| **17** | **17** | **2** | **21** | **21** | **1** | **25** | **22** | **2** | **32** | **3** | **1** |
| **17** | **19** | **1** | **21** | **23** | **2** | **25** | **24** | **1** | **32** | **5** | **1** |
| **17** | **20** | **2** | **21** | **25** | **3** | **26** | **15** | **1** | **33** | **2** | **1** |
| **17** | **21** | **1** | **22** | **15** | **1** | **26** | **16** | **1** |  |  |  |

**Table S10b.** Burgess Shale (*S* = 48, *L* = 249)

| **cons** | **res** | **cert** | **cons** | **res** | **cert** | **cons** | **res** | **cert** | **cons** | **res** | **cert** | **cons** | **res** | **cert** |
| --- | --- | --- | --- | --- | --- | --- | --- | --- | --- | --- | --- | --- | --- | --- |
| **7** | **1** | **2** | **26** | **14** | **2** | **28** | **30** | **2** | **33** | **48** | **1** | **38** | **44** | **1** |
| **7** | **2** | **2** | **26** | **17** | **2** | **28** | **31** | **2** | **34** | **19** | **2** | **38** | **48** | **1** |
| **7** | **7** | **2** | **26** | **18** | **2** | **28** | **32** | **2** | **34** | **20** | **2** | **39** | **3** | **1** |
| **8** | **2** | **2** | **26** | **25** | **2** | **28** | **33** | **2** | **34** | **21** | **2** | **40** | **11** | **1** |
| **9** | **7** | **2** | **26** | **39** | **2** | **28** | **35** | **2** | **34** | **22** | **2** | **40** | **12** | **1** |
| **10** | **7** | **2** | **26** | **40** | **2** | **28** | **38** | **2** | **34** | **23** | **2** | **40** | **13** | **1** |
| **10** | **9** | **2** | **26** | **41** | **2** | **28** | **39** | **2** | **34** | **25** | **2** | **40** | **14** | **1** |
| **11** | **3** | **1** | **26** | **42** | **2** | **28** | **42** | **2** | **34** | **26** | **2** | **40** | **18** | **1** |
| **11** | **7** | **1** | **26** | **48** | **2** | **28** | **43** | **2** | **34** | **27** | **2** | **40** | **38** | **1** |
| **12** | **5** | **1** | **27** | **11** | **2** | **29** | **11** | **1.5** | **34** | **28** | **2** | **40** | **39** | **1** |
| **12** | **6** | **1** | **27** | **12** | **2** | **29** | **12** | **1.5** | **34** | **29** | **2** | **40** | **42** | **1** |
| **12** | **7** | **2** | **27** | **13** | **2** | **29** | **13** | **1.5** | **34** | **30** | **2** | **40** | **44** | **1** |
| **13** | **4** | **2** | **27** | **14** | **2** | **29** | **14** | **1.5** | **34** | **31** | **2** | **40** | **48** | **1** |
| **14** | **4** | **1** | **27** | **18** | **2** | **29** | **18** | **1.5** | **34** | **32** | **2** | **41** | **11** | **3** |
| **15** | **2** | **1** | **27** | **19** | **2** | **29** | **38** | **1.5** | **34** | **33** | **2** | **41** | **12** | **1** |
| **15** | **4** | **1** | **27** | **20** | **2** | **29** | **39** | **1.5** | **34** | **35** | **2** | **41** | **13** | **1** |
| **15** | **6** | **1** | **27** | **21** | **2** | **29** | **42** | **1.5** | **34** | **36** | **2** | **41** | **14** | **1** |
| **16** | **7** | **1.75** | **27** | **22** | **2** | **29** | **44** | **1.5** | **35** | **7** | **1** | **41** | **18** | **1** |
| **17** | **8** | **1** | **27** | **23** | **2** | **29** | **48** | **1.5** | **35** | **19** | **2** | **41** | **38** | **1** |
| **18** | **7** | **2** | **27** | **25** | **2** | **30** | **13** | **2** | **35** | **20** | **2** | **41** | **39** | **1** |
| **19** | **4** | **1** | **27** | **26** | **2** | **30** | **14** | **2** | **35** | **24** | **2** | **41** | **42** | **1** |
| **20** | **4** | **1** | **27** | **27** | **2** | **30** | **17** | **2** | **35** | **26** | **2** | **41** | **44** | **1** |
| **20** | **11** | **1** | **27** | **28** | **2** | **30** | **18** | **2** | **35** | **29** | **2** | **41** | **48** | **1** |
| **20** | **12** | **1** | **27** | **29** | **2** | **30** | **25** | **2** | **35** | **31** | **2** | **42** | **11** | **1** |
| **20** | **13** | **1** | **27** | **30** | **2** | **30** | **39** | **2** | **36** | **7** | **1** | **42** | **12** | **1** |
| **20** | **14** | **1** | **27** | **31** | **2** | **30** | **40** | **2** | **36** | **19** | **2** | **42** | **13** | **1** |
| **20** | **18** | **1** | **27** | **32** | **2** | **30** | **41** | **2** | **36** | **20** | **2** | **42** | **14** | **1** |
| **20** | **38** | **1** | **27** | **33** | **2** | **30** | **42** | **2** | **36** | **24** | **2** | **42** | **18** | **1** |
| **20** | **39** | **1** | **27** | **35** | **2** | **30** | **48** | **2** | **36** | **26** | **2** | **42** | **38** | **1** |
| **20** | **42** | **1** | **27** | **38** | **2** | **31** | **13** | **1** | **36** | **29** | **2** | **42** | **39** | **1** |
| **20** | **44** | **1** | **27** | **39** | **2** | **31** | **14** | **1** | **36** | **31** | **2** | **42** | **42** | **1** |
| **20** | **48** | **1** | **27** | **42** | **2** | **31** | **17** | **1** | **37** | **11** | **2** | **42** | **44** | **1** |
| **21** | **11** | **1.03** | **27** | **43** | **2** | **31** | **18** | **1** | **37** | **13** | **2** | **42** | **48** | **1** |
| **21** | **12** | **1.03** | **27** | **44** | **2** | **31** | **25** | **1** | **37** | **14** | **2** | **43** | **7** | **1** |
| **21** | **13** | **1.03** | **27** | **48** | **2** | **31** | **39** | **1** | **37** | **18** | **2** | **44** | **7** | **1** |
| **21** | **14** | **1.03** | **28** | **11** | **2** | **31** | **40** | **1** | **37** | **38** | **2** | **45** | **1** | **2** |
| **21** | **18** | **1.03** | **28** | **12** | **2** | **31** | **41** | **1** | **37** | **39** | **2** | **45** | **7** | **2** |
| **21** | **38** | **1.03** | **28** | **13** | **2** | **31** | **42** | **1** | **37** | **40** | **2** | **46** | **3** | **1** |
| **21** | **39** | **1.03** | **28** | **14** | **2** | **31** | **48** | **1** | **37** | **41** | **2** | **46** | **7** | **1** |
| **21** | **42** | **1.03** | **28** | **18** | **2** | **32** | **1** | **2** | **37** | **42** | **2** | **47** | **11** | **2** |
| **21** | **44** | **1.03** | **28** | **19** | **2** | **32** | **3** | **2** | **37** | **44** | **2** | **47** | **13** | **2** |
| **21** | **48** | **1.03** | **28** | **20** | **2** | **32** | **7** | **2** | **37** | **48** | **2** | **47** | **14** | **2** |
| **22** | **4** | **1.03** | **28** | **21** | **2** | **33** | **14** | **1** | **38** | **11** | **2** | **47** | **18** | **2** |
| **23** | **7** | **2** | **28** | **22** | **2** | **33** | **17** | **1** | **38** | **12** | **2** | **47** | **38** | **2** |
| **24** | **1** | **1** | **28** | **23** | **2** | **33** | **18** | **1** | **38** | **13** | **2** | **47** | **39** | **2** |
| **24** | **4** | **1** | **28** | **25** | **2** | **33** | **25** | **1** | **38** | **14** | **2** | **47** | **40** | **2** |
| **24** | **7** | **1** | **28** | **26** | **2** | **33** | **39** | **1** | **38** | **18** | **1** | **47** | **44** | **2** |
| **25** | **4** | **2** | **28** | **27** | **2** | **33** | **40** | **1** | **38** | **38** | **2** | **47** | **48** | **2** |
| **26** | **11** | **2** | **28** | **28** | **2** | **33** | **41** | **1** | **38** | **39** | **2** | **48** | **7** | **1.5** |
| **26** | **13** | **2** | **28** | **29** | **2** | **33** | **42** | **1** | **38** | **42** | **2** |  |  |  |
