# Supplementary material for: Compilation and Network Analyses of Cambrian Food Webs
Source: PLoS Biol. 2008 Apr 29;6(4):e102. doi: 10.1371/journal.pbio.0060102 (PMC2689700; doi:10.1371/journal.pbio.0060102)
Supplement: Table S11 — (91 KB DOC) [file pbio.0060102.st011.doc]

**Table S11.** Amount of low-certainty links at different degree levels in two Cambrian food webs

**Table S11a. Chengjiang**

| # links | *S* | % low cert. |  | # cons. | *S* | % low cert. |  | # res. | *S* | % low cert. |
| --- | --- | --- | --- | --- | --- | --- | --- | --- | --- | --- |
| 1 | 6 | 66.7 |  | 1 | 6 | 83.3 |  | 1 | 12 | 41.7 |
| 2 | 5 | 80.0 |  | 2 | 6 | 58.3 |  | 2 | 6 | 75.0 |
| 3 | 4 | 66.7 |  | 4 | 4 | 37.5 |  | 3 | 1 | 66.7 |
| 4 | 5 | 60.0 |  | 7 | 3 | **0.0** |  | 4 | 1 | 100.0 |
| 5 | 1 | 100.0 |  | 8 | 1 | **62.5** |  | 6 | 2 | 91.7 |
| 8 | 1 | 50.0 |  | 9 | 4 | **100.0** |  | 7 | 1 | 57.1 |
| 10 | 6 | ***61.7*** |  |  |  |  |  | 8 | 5 | *50.0* |
| 14 | 1 | ***28.6*** |  |  |  |  |  | 9 | 1 | *44.4* |
| 15 | 2 | ***63.3*** |  |  |  |  |  |  |  |  |
| 16 | 1 | *25.0* |  |  |  |  |  |  |  |  |
| 17 | 1 | 76.5 |  |  |  |  |  |  |  |  |

**Table S11b. Burgess**

| # links | *S* | % low cert. |  | # cons. | *S* | % low cert. |  | # res. | *S* | % low cert. |
| --- | --- | --- | --- | --- | --- | --- | --- | --- | --- | --- |
| 1 | 2 | 50.0 |  | 1 | 4 | 50.0 |  | 1 | 15 | 46.7 |
| 2 | 6 | 41.7 |  | 2 | 3 | 33.3 |  | 2 | 4 | 50.0 |
| 3 | 3 | ***55.6*** |  | 3 | 10 | 3.3 |  | 3 | 5 | 53.3 |
| 4 | 4 | ***31.3*** |  | 4 | 3 | 50.0 |  | 7 | 2 | 14.3 |
| 5 | 2 | ***60.0*** |  | 5 | 6 | 6.7 |  | 9 | 2 | **50.0** |
| 6 | 2 | ***8.3*** |  | 6 | 1 | 33.3 |  | 10 | 8 | **65.0** |
| 8 | 3 | 37.5 |  | 7 | 1 | 28.6 |  | 11 | 3 | 33.3 |
| 9 | 1 | 0.0 |  | 8 | 1 | 75.0 |  | 16 | 1 | 0.0 |
| 10 | 1 | 10.0 |  | 9 | 1 | 55.6 |  | 24 | 1 | 0.0 |
| 11 | 2 | 31.8 |  | 10 | 1 | 60.0 |  | 26 | 1 | 0.0 |
| 12 | 2 | 66.7 |  | 11 | 1 | **45.5** |  |  |  |  |
| 13 | 2 | **38.5** |  | 12 | 1 | **33.3** |  |  |  |  |
| 14 | 1 | **42.9** |  | 14 | 3 | **50.0** |  |  |  |  |
| 15 | 5 | **46.7** |  | 15 | 3 | **48.9** |  |  |  |  |
| 16 | 7 | **42.0** |  | 17 | 1 | **41.2** |  |  |  |  |
| 20 | 1 | 35.0 |  |  |  |  |  |  |  |  |
| 21 | 1 | 38.1 |  |  |  |  |  |  |  |  |
| 24 | 1 | 70.8 |  |  |  |  |  |  |  |  |
| 27 | 1 | 0.0 |  |  |  |  |  |  |  |  |
| 29 | 1 | 0.0 |  |  |  |  |  |  |  |  |

**Table S11 Footnotes.** Degree levels indicated by **# links** (total links), **# cons.** (vulnerability—number of links from consumers), and **# res.** (generality—number of links to resources). ***S***: number of trophic species at a particular degree. **% low cert.**: percentage of links associated with a particular degree that are low certainty (certainty <1.5). Italicized numbers indicate % of links that are low certainty associated with points in the empirical normalized degree distributions that fall outside the variability seen across modern webs (Figure 4). Bold numbers indicate % of links that are low certainty associated with points in the empirical Cambrian degree distributions that fall outside the 95% confidence intervals for comparable niche model simulations (Figure 7).
