# Supplementary material for: Compilation and Network Analyses of Cambrian Food Webs
Source: PLoS Biol. 2008 Apr 29;6(4):e102. doi: 10.1371/journal.pbio.0060102 (PMC2689700; doi:10.1371/journal.pbio.0060102)
Supplement: Table S12 — (62 KB DOC) [file pbio.0060102.st012.doc]

**Table S12.** Niche model errors for ten modern webs

|  | Bridge Brook | Skipwith | Benguela | Coachella | Chesapeake | St. Martin | St. Marks | Reef | Ythan | Broom |
| --- | --- | --- | --- | --- | --- | --- | --- | --- | --- | --- |
| Top | 1.00 | -0.33 | 1.00 | 0.00 | **-1.50** | -0.83 | -1.00 | 1.00 | **-3.12** | **-5.25** |
| Int | 0.40 | -0.50 | -1.00 | 0.00 | 0.40 | 0.43 | 0.25 | -1.00 | **1.27** | **1.90** |
| Bas | **-1.50** | 1.00 | 0.67 | 0.00 | **1.33** | 0.50 | 0.75 | 0.50 | **2.20** | **4.40** |
| Herb | **-1.67** | **-7.00** | -0.50 | -1.00 | 0.00 | **-2.00** | -0.75 | -0.50 | **-2.71** | -0.71 |
| Can | 0.50 | 0.75 | 0.40 | **-1.50** | 0.50 | **1.67** | 0.67 | **-1.40** | 0.25 | -0.67 |
| Omn | **1.50** | **1.25** | 0.25 | 0.25 | -0.75 | 0.33 | -0.80 | -0.33 | 0.67 | **1.22** |
| Loop | 0.00 | 1.00 | 0.33 | -0.50 | 0.00 | 1.00 | 1.00 | **-1.44** | 0.00 | 0.00 |
| ChLen | **1.15** | 0.71 | 0.62 | 0.87 | 0.23 | 0.81 | -0.02 | 0.25 | 0.92 | -0.02 |
| ChSD | **1.56** | 0.51 | 0.56 | 0.83 | 0.36 | 0.89 | 0.11 | -0.43 | **1.21** | 0.83 |
| ChNum | 0.59 | 0.78 | 0.87 | 0.58 | -0.01 | 0.55 | -0.43 | 0.25 | 0.67 | -0.25 |
| TL | **1.15** | 0.80 | -0.08 | 0.64 | -0.42 | 0.45 | 0.04 | 0.65 | -0.35 | **-2.62** |
| MaxSim | **-1.29** | 0.31 | 0.77 | 0.61 | -0.07 | **1.24** | **2.66** | **3.57** | **1.90** | -0.02 |
| VulSD | -0.33 | -0.77 | 0.18 | 0.58 | 1.55 | 0.33 | **1.69** | 0.22 | 0.29 | **1.39** |
| GenSD | 0.01 | -0.22 | **-1.06** | -0.68 | **-1.84** | -0.86 | **-1.52** | -0.45 | **-4.70** | **-6.86** |
| LinkSD | -0.02 | 0.63 | 0.04 | -0.02 | 0.09 | 0.10 | 0.20 | -0.83 | **-2.98** | **-3.81** |
| Path | -0.85 | 0.52 | -0.40 | -0.44 | -0.58 | 0.48 | 0.01 | -0.01 | **1.01** | -0.64 |
| Clust | **1.14** | **1.48** | 0.74 | -0.09 | 0.41 | 0.86 | 0.55 | -0.56 | **-1.09** | **-1.40** |

**Table S12 Footnotes**: MEs that fall within ±1 are considered to show a good fit of the model to the data. MEs that fall outside ±1 are shown in bold.
