# Supplementary material for: Compilation and Network Analyses of Cambrian Food Webs
Source: PLoS Biol. 2008 Apr 29;6(4):e102. doi: 10.1371/journal.pbio.0060102 (PMC2689700; doi:10.1371/journal.pbio.0060102)
Supplement: Table S13 — (52 KB DOC) [file pbio.0060102.st013.doc]

**Table S13.** Random-beta model analysis results for two Cambrian food webs

|  | Chengjiang | | | Burgess | | |
| --- | --- | --- | --- | --- | --- | --- |
| Property | obs. | random-beta | ME | obs. | random-beta | ME |
| Top | 0.039 | 0.273 | **-2.67** | 0.019 | 0.167 | **-2.00** |
| Int | 0.800 | 0.606 | **1.40** | 0.879 | 0.708 | **2.00** |
| Bas | 0.160 | 0.121 | 0.33 | 0.102 | 0.125 | -0.33 |
| Herb | 0.037 | 0.242 | **-3.50** | 0.012 | 0.167 | **-4.00** |
| Can | 0.086 | 0.242 | **-1.67** | 0.105 | 0.104 | 0.00 |
| Omn | 0.678 | 0.455 | **1.75** | 0.805 | 0.542 | **2.60** |
| Loop | 0.721 | 0.212 | **1.89** | 0.858 | 0.083 | **5.43** |
| ChLen | 7.838 | 4.787 | **1.79** | 10.340 | 6.197 | **2.77** |
| ChSD | 2.060 | 1.336 | **1.77** | 2.124 | 1.286 | **2.90** |
| ChNum | 3.426 | 2.427 | **1.60** | 5.256 | 3.858 | **1.83** |
| TL | 11.450 | 2.841 | 0.85 | 10.430 | 2.721 | **1.26** |
| MaxSim | 0.479 | 0.554 | **-1.22** | 0.535 | 0.670 | **-2.49** |
| VulSD | 0.530 | 1.057 | **-2.56** | 0.462 | 0.947 | **-2.85** |
| GenSD | 0.778 | 0.981 | **-1.24** | 0.708 | 1.152 | **-3.06** |
| LinkSD | 0.474 | 0.838 | **-3.76** | 0.430 | 0.683 | **-3.03** |
| Path | 2.285 | 2.909 | **-3.33** | 2.030 | 2.164 | **-1.26** |
| Clust | 0.116 | 0.234 | **-1.50** | 0.136 | 0.234 | **-1.79** |

**Table S13 Footnotes. obs.**: the empirical value of the property for that web. **random-beta**: the mean random-beta model value across 1000 model webs. **ME**: model error. MEs that fall within ±1 are considered to show a good fit of the model to the data. MEs that fall outside of ±1 are shown in bold.
