# Supplementary material for: Compilation and Network Analyses of Cambrian Food Webs
Source: PLoS Biol. 2008 Apr 29;6(4):e102. doi: 10.1371/journal.pbio.0060102 (PMC2689700; doi:10.1371/journal.pbio.0060102)
Supplement: Table S14 — (59 KB DOC) [file pbio.0060102.st014.doc]

**Table S14.** Variability of niche model mean values and model errors across six runs of the niche model for two Cambrian food webs

|  | Chengjiang | | | | | Burgess | | | | |
| --- | --- | --- | --- | --- | --- | --- | --- | --- | --- | --- |
| Property | CV | low | high | low ME | high ME | CV | low | high | low ME | high ME |
| Top | 2.2 | 0.100 | 0.106 | -1.20 | -1.20 | 3.5 | 0.069 | 0.076 | -0.71 | -0.83 |
| Int | 0.4 | 0.647 | 0.653 | 0.20 | 0.33 | 0.3 | 0.745 | 0.751 | 0.25 | 0.29 |
| Bas | 0.4 | 0.246 | 0.248 | 1.00 | 1.33 | 1.1 | 0.177 | 0.183 | 0.60 | 0.75 |
| Herb | 1.1 | 0.140 | 0.144 | -0.75 | -1.00 | 0.8 | 0.088 | 0.090 | -1.00 | -1.00 |
| Can | 1.8 | 0.084 | 0.089 | -1.67 | -2.50 | 0.9 | 0.112 | 0.114 | 0.00 | 0.00 |
| Omn | 0.5 | 0.513 | 0.519 | 0.33 | 0.40 | 0.2 | 0.648 | 0.653 | 0.83 | 0.83 |
| Loop | 3.4 | 0.024 | 0.026 | -1.75 | -2.33 | 3.5 | 0.058 | 0.063 | -0.33 | -0.40 |
| ChLen | 0.3 | 4.963 | 5.007 | 0.03 | 0.11 | 0.6 | 7.029 | 7.149 | 0.41 | 0.47 |
| ChSD | 0.5 | 1.453 | 1.473 | 0.24 | 0.28 | 0.5 | 1.717 | 1.739 | 0.98 | 1.12 |
| ChNum | 0.2 | 2.788 | 2.804 | 0.78 | 0.86 | 0.4 | 4.161 | 4.201 | 0.39 | 0.46 |
| TL | 0.4 | 2.325 | 2.346 | -0.87 | -0.98 | 0.5 | 2.620 | 2.662 | -0.15 | -0.22 |
| MaxSim | 0.2 | 0.551 | 0.555 | -0.07 | 0.01 | 0.1 | 0.624 | 0.626 | -0.85 | -0.98 |
| VulSD | 0.4 | 0.656 | 0.663 | -1.67 | -1.86 | 0.5 | 0.611 | 0.620 | -1.62 | -1.89 |
| GenSD | 0.5 | 1.144 | 1.134 | 0.53 | 0.59 | 0.4 | 1.098 | 1.104 | -0.20 | -0.30 |
| LinkSD | 0.4 | 0.555 | 0.560 | -2.05 | -2.20 | 0.3 | 0.528 | 0.532 | -1.40 | -1.51 |
| Path | 0.2 | 2.232 | 2.242 | -2.82 | -3.19 | 0.1 | 2.008 | 2.011 | -1.19 | -1.26 |
| Clust | 0.7 | 0.158 | 0.161 | -0.86 | -0.95 | 0.4 | 0.186 | 0.188 | -0.74 | -0.82 |

**Table S14 Footnotes. CV**: coefficient of variation for niche model mean values (standard deviation/mean*100; n = 6). **low**: low mean model value out of 6 runs. **high**: high mean model value out of 6 runs. **low ME**: low ME out of 6 runs. **high ME**: high ME out of 6 runs. The 6 runs include the original run (Table 2) and 5 additional runs. MEs that fall within ±1 are considered to show a good fit of the model to the data. In only one case did MEs straddle |1|: for Burgess ChSD, two runs returned MEs of 0.98 and 0.99 and four runs returned MEs of 1.01 to 1.12. In no case did a change in ME alter the comparative status of a Cambrian structural property with reference to the 95% modern web confidence intervals for that property (Figure 6).
